# Toward autonomous artificial intelligence agents in sports science: a modular framework for development, validation, and implementation

**DOI:** 10.5114/biolsport.2026.161708

**Published:** 2026-05-12

**Authors:** Ismail Dergaa, Sabri Barbaria, Wissem Dhahbi, Piotr Zmijewski, Karim Chamari, Helmi Ben Saad

**Affiliations:** 1 High Institute of Sport and Physical Education of Ksar Said, University of Manouba, Manouba, Tunisia; 2 Research Unit Physical Activity, Sport, and Health, UR18JS01, National Observatory of Sport, Tunis 1003, Tunisia; 3 High Institute of Sport and Physical Education of Kef, University of Jendouba, El Kef, Tunisia; 4 Laboratory of Biophysics and Medical Technologies, LR13ES07 (BTM), Higher Institute of Medical Technologies of Tunis (ISTMT), University of Tunis El Manar, Tunis 1080, Tunisia; 5 Training Department, Police College, Qatar Police Academy, Doha, Qatar; 6 Jozef Pilsudski University of Physical Education in Warsaw, Warsaw, Poland; 7 Research Office, Naufar Center, Doha, Qatar; 8 Faculty of Medicine of Sousse, University of Sousse, Farhat Hached University Hospital, Laboratory of Physiology, Sousse, Tunisia; 9 University of Sousse, Faculty of Medicine of Sousse, Farhat Hached University Hospital, Laboratory research LR12SP09 “Heart failure” Sousse, Tunisia; 10 University Central Group, UPSAT-Sousse, CRCI, Tunis, Tunisia

**Keywords:** AI agents, Algorithmic bias, Athletic performance, Agentic frameworks, ChatGPT, Computer vision, Exercise prescription, Human oversight, Implementation science, Injury prevention, Large Language Models, Multi-agent coordination, Multi-agent systems, Sports science, Training Load Management, Wearables

## Abstract

Despite widespread artificial intelligence (AI) adoption in sports science for predictive analytics, current systems operate as passive tools requiring continuous human monitoring and intervention at every decision point. Autonomous AI agent systems capable of 24/7 monitoring, independent reasoning, and proactive action execution remain academically unexplored in sports science contexts. Unlike passive analytics that await human analysis or conversational interfaces requiring explicit prompting, autonomous agents operate continuously, detecting patterns and implementing interventions without human initiation. Our review distinguished autonomous agents from existing AI applications, proposes modular implementation frameworks, develops theoretical application workflows across eight priority domains, and establishes empirical validation pathways. Current (i.e., April 23, 2026) literature lacks peer-reviewed research on autonomous agent systems in sports science. This review connects computer science with exercise physiology. We integrate modern agent architectures with established sports science concepts. The outcome is a practical, multi-domain implementation roadmap. Our three-phase framework progresses from specialized single-domain agents through coordinated multi-agent systems to fully integrated platforms. *Phase 1* develops autonomous agents for training load management, exercise prescription, biomechanical analysis, nutrition optimization, sleep monitoring, injury prevention, mental skills training, and rehabilitation, each operating independently within defined safety boundaries. *Phase 2* stablishes coordination protocols enabling information exchange across domains while maintaining modular independence. *Phase 3* integrates fully autonomous agent systems across all domains into a unified platform with comprehensive cross-domain reasoning. This framework aimed to advance autonomous agent research from conceptual proposal to a structured implementation and validation pathway in evidence-based athlete management.

## ABBREVIATIONS LIST

**ACL:** Anterior Cruciate Ligament

**ACWR:** Acute–Chronic Workload Ratio

**AI:** Artificial Intelligence

**API:** Application Programming Interface

**bpm:** Beats per Minute

**FITT:** Frequency, Intensity, Time, Type

**GPS:** Global Positioning System

**GPT:** Generative Pre-trained Transformer

**HR:** Heart Rate

**HRmax:** Maximal Heart Rate

**RHR:** Resting Heart Rate

**HRV:** Heart Rate Variability

**IT:** Information Technology

**LLM:** Large Language Model

**ML:** Machine Learning

**PSQI:** Pittsburgh Sleep Quality Index

**RCT:** Randomized Controlled Trial

**REM:** Rapid Eye Movement

**RHR:** Resting Heart Rate

**RPE:** Rating of Perceived Exertion

**sRPE:** Session-Rating of Perceived Exertion

**T2DM:** Type 2 Diabetes Mellitus

**TLMA:** Training Load Management Agent

$\dot{V}O_{2max}$: Maximal Oxygen Uptake

**1RM:** One-Repetition Maximum

## 1 INTRODUCTION

Sports science technology has evolved considerably over the past two decades (i.e., 2005–2025), progressing from basic performance metrics to sophisticated analytical ecosystems [1]. Professional organizations have deployed extensive sensor networks, cloud platforms, and machine learning (ML) systems [2, 3]. The global artificial intelligence (AI) in sports market expanded from $8.93 billion in 2024 with projections reaching $60.78 billion by 2034 [4]. Elite athletes routinely wear multiple sensors generating vast physiological and biomechanical datasets [5]. Sports scientists allocate substantial time to reviewing dashboards, interpreting analytical outputs, and synthesizing information from independent systems [6]. However, this technological investment conceals a fundamental limitation. Current (i.e., April 2026) AI applications operate as passive tools, requiring active human-initiated monitoring at each stage of the analytical workflow [7, 8]. For instance, an athlete completes training while equipped with monitoring devices, and the data are subsequently uploaded to analytical platforms [9, 10]. The algorithms then generate outputs, such as fatigue scores, injury risk predictions, or recovery recommendations, which are displayed on dashboards [9]. Sports scientists are required to log in, review the results, interpret the findings within individual contexts, make decisions regarding actions, communicate recommendations through separate channels, and oversee the implementation on the field [11]. The entire workflow is contingent on human intervention at every transition point [8]. Under current sports science deployments, these systems do not independently perceive emerging problems, reason about appropriate responses, or autonomously implement interventions without explicit human direction [8]. The aforementioned limitation is critical when managing athletic populations. A professional team might monitor 25–30 athletes daily across training load, recovery, nutrition, sleep, and injury risks [8]. Each domain generates independent data streams that are processed using separate systems [11]. Comprehensive athlete management requires the manual integration of information from multiple platforms, reconciliation of potentially conflicting recommendations, prioritization of interventions given limited resources, and documentation for accountability [12]. The associated cognitive burden on the staff is, to say the least, substantial [13]. Important patterns may remain unrecognized when data from independent platforms are not integrated in a timely manner interventions [14]. Real-time adjustment opportunities during training sessions are unrecognized because practitioners cannot continuously monitor data while managing other responsibilities [8].

Technology exists to address these limitations through AI agent systems that have emerged as fundamentally different computational approaches [15, 16]. These systems operate autonomously, rather than awaiting human queries [15, 16]. They continuously monitor environments, detect patterns warranting attention, reason about appropriate responses, implement interventions through available tools, and learn from outcomes to improve future decisions [15, 16]. Healthcare has begun to deploy agents for patient monitoring and clinical support [17–19]. Manufacturing uses multi-agent systems for supply chain optimization [20]. Financial services employ autonomous agents for fraud detection [21]. To date (i.e., April 23, 2026), peer-reviewed research explicitly examining fully autonomous, continuously operating AI agent systems in sports science contexts remains absent from the literature, representing a substantive knowledge gap [22, 23].

Contemporary literature applies multiple overlapping terms to these systems. Computer science defines an agent as any computational entity that perceives its environment and takes actions to achieve goals [15], encompassing architectures from simple reflex systems to sophisticated autonomous frameworks. Control theory, robotics, and software engineering favor “autonomous agents”, “intelligent agents”, and “agent-based systems”, respectively [16], whereas industry publications increasingly use “agentic AI” for systems combining language-model reasoning with tool execution [22]. For this review, “AI agents” is the standard term, aligning with established computer science nomenclature [15]. “AI agent systems” denotes deployment architectures encompassing multiple agents and supporting infrastructure. Where operational independence requires emphasis, ‘autonomous AI agents’ distinguishes continuous, proactive systems from passive analytics or reactive conversational interfaces.

The distinction between current sports science AI tools and AI agents requires careful examination because the terminology has become confusing. At the time of this review, (i.e., April 23, 2026), many use “AI” as an umbrella term covering everything from basic statistics to advanced autonomous systems [24]. The proliferation of conversational AI tools, such as Chat Generative Pre-trained Transformer (ChatGPT), has further complicated this misunderstanding. Athletes and coaches are increasingly experimenting with these tools for training advice and nutritional guidance [8]. However, these conversational interfaces differ fundamentally from AI agents [22]. A significant barrier to understanding this shift is the confusion between ‘Generative AI’ tools (e.g., ChatGPT) and ‘Autonomous AI Agents’. While conversational interfaces are useful for drafting text or answering queries, they remain fundamentally reactive; they wait for a prompt. In contrast, an autonomous AI agent connects directly to the data environment to perceive and act without explicit human initiation. This distinction is architecturally critical yet often misunderstood [25, 26]. Future large language model (LLM) iterations [26, 27] may provide the reasoning infrastructure for these agents, but the autonomous loop requires connections to external tools that current conversational interfaces typically lack. Research has documented substantial deficiencies in applying conversational AI to specialized sports science tasks. Dergaa et al. [8] evaluated ChatGPT for exercise prescription and reported that generic recommendations lacked individualization, failed to adequately account for medical contraindications, included inappropriate exercise progressions, and were inconsistent across repeated queries [8]. Similar problems have emerged in mental health assessment, nutrition counselling, resistance training prescription [28–30], and sports medicine statistical methodology, where ChatGPT failed in 3 of 4 sample-size calculations [31]. These limitations stem from the design characteristics. Conversational AI serves as a general-purpose interface optimized for plausible-sounding responses rather than medically appropriate, individually tailored, evidence-based recommendations that account for complex contraindications. Moreover, research suggests that excessive reliance on AI-generated recommendations may impair human cognitive development [32]. Dergaa et al. [32] documented how dependence on AI outputs can diminish the critical thinking abilities essential for expert practice. This concern is particularly salient because AI systems, regardless of their sophistication, fundamentally lack clinical judgment. Clinical reasoning requires the integration of subtle contextual cues, recognition of atypical presentations, application of ethical judgment in ambiguous situations, and flexible adaptation to changing circumstances [33, 34]. These capabilities emerge from extensive deliberate practice, embodied experience, and supervised mentorship, and they are not currently replicable by AI systems irrespective of computational scale [33, 34].

AI agents represent something categorically different from both traditional analytics and conversational interfaces. An AI agent operates as an autonomous system with capabilities that current sports science tools lack. First, continuous environmental monitoring replaces episodic analysis [22]. The agent constantly observes data streams as information arrives rather than waiting for practitioner dashboard checks [22]. Second, independent reasoning allows for situation evaluation and response determination without explicit human instructions for every scenario [22]. Third, action execution through tool access enables the implementation of autonomous decisions [22]. The agent may modify training schedules, send stakeholder notifications, schedule assessments, and escalate complex situations for human oversight. Fourth, multi-agent communication permits coordination across specialized domains [23]. A trainingload management-agent (TLMA) may share information with nutrition and recovery agents to synchronize the recommendations. Fifth, outcome-based learning allows for performance improvement over time as the agent observes which interventions succeed or fail [35, 36]. The following theoretical scenario illustrates how such an agent could operate in principle: An athlete wears a smartwatch that continuously tracks heart rate (HR), HR variability (HRV), sleep quality, and daily activity. A TLMA monitored this data stream in real time. During a scheduled high-intensity session, the agent observed blunted HR responses. The athlete’s HR fails to reach the target zones despite work rates typically producing higher cardiovascular stress. This pattern may indicate insufficient recovery, accumulating fatigue, and/or illness onset. Traditional analytics may flag this as an anomaly in an evening report. The sports scientist sees the flag the next morning when checking the dashboards. After review and consultation, the practitioner may decide to modify subsequent training. This process would traditionally take 12–24 hours from the time of pattern occurrence until intervention implementation. An AI agent operates differently. It detects blunted responses immediately during the session. It accesses the athlete’s recent training history and wellness scores for contextualization. In this hypothetical scenario, the agent would identify that blunted responses combined with recent high training loads warrant an immediate intervention. It would automatically reduce the intensity prescription for the remaining intervals. Simultaneously, it would send a notification to the supervising coach explaining the concern and modification required. It would schedule a follow-up wellness check for the following morning. If the athlete’s condition were to worsen overnight based on sleep quality and HRV data, the agent would escalate to the medical staff, recommending rest rather than modified training.

The notification capability deserves particular attention because it may transform passive monitoring into active athlete management. Current systems require someone to actively check dashboards to discover problems. In a fully implemented system, agents would initiate communication when monitoring thresholds are crossed. These notifications take various forms depending on the urgency and context. Email notifications might inform coaches that an athlete has failed to reach the prescribed training intensity during previous sessions. Mobile push notifications can alert immediately when smartwatch data indicates training load limit exceedances. Concerns that are more serious may trigger direct calls to the medical staff. The proposed sophistication of such systems would lie in their capacity to analyze multiple data streams simultaneously, recognize complex patterns rather than simple threshold violations, prioritize alerts based on clinical significance rather than statistical deviation alone, and learn from response patterns to calibrate future notification decisions [37].

This technological capability intersects with crucial practical considerations regarding access to expert supervision. Two distinct implementation scenarios exist, each with different implications. The optimal scenario involves agents supporting expert practitioners who retain ultimate authority. The agent continuously monitors athlete data, detects patterns warranting attention, generates preliminary recommendations based on evidence-based algorithms, and presents suggestions to qualified sports scientists or coaches for review. Human experts evaluate agent recommendations using clinical judgment, contextual knowledge, and an understanding of individual circumstances that algorithms cannot fully replicate [38, 39]. This model represents the best practice because AI systems, regardless of their sophistication, lack the fundamental aspects of human cognition that are essential for optimal decision-making in complex domains [8].

A suboptimal but pragmatically necessary scenario involves athletes using agents without expert supervision. Geographic isolation, financial constraints, and limited specialist availability create situations in which automated guidance exceeds unstructured support. An individual in a rural area lacking access to sports scientists might benefit from an exercise prescription agent despite the lack of professional oversight. Individuals managing type 2 diabetes mellitus (T2DM) through exercise might improve their health outcomes using automated programming, despite lacking access to clinical exercise physiologists. However, this approach introduces risks. Agents may generate inappropriate recommendations for complex medical presentations. Subtle warning signs indicating serious problems may go unrecognized. Motivation and adherence challenges remain unaddressed in the absence of human support. However, pragmatic health equity considerations suggest that access to evidence-based automated guidance may, albeit speculatively, produce better outcomes than the complete absence of structured programming for underserved populations, though empirical validation in sports contexts is lacking [40, 41].

Regardless of the implementation scenario, qualified practitioners must retain final authority over decision-making. The agent serves as a sophisticated tool that augments human judgment rather than replacing it. This principle stems from the fundamental limitations of AI, which persist despite remarkable recent advances. AI systems excel in pattern recognition within training distributions [42]. The path forward requires strategic thinking about implementation rather than attempting to integrate comprehensively. Technology adoption research demonstrates that successful implementations consistently begin with focused, high-value applications before expanding toward broader integration [43, 44]. Consumer electronics provide instructive precedents. *Apple* introduced the *iPod* as a specialized music device in 2001, establishing the technical infrastructure and user confidence that later enabled the *iPhone* in 2007, each phase delivering standalone value independent of subsequent developments [45]. The success of the *iPod* created the foundation for introduction of the *iPhone* in 2007, integrating telephony, mobile computing, and media playback [46]. Each phase provided distinct functional benefits and incremental system maturity. The development of sports science AI agents should follow similar principles. Rather than attempting to develop comprehensive systems that handle all aspects of athlete management immediately, the field should prioritize specialized single-domain agents that effectively address specific challenges. Training load management represents an ideal starting point, given the abundant wearable device data, clear outcome metrics through injury rates and performance measures, and well-established evidence-based guidelines for load-response relationships [47, 48]. An autonomous TLMA that monitors workload patterns and adjusts prescriptions within preset parameters could provide immediate practical value. This focused application would allow systematic agent performance validation, provide learning opportunities regarding human-agent interaction patterns, establish technical infrastructure supporting subsequent agent development, and build confidence in autonomous systems among practitioners and athletes.

Exercise prescription is another high-priority domain. Current approaches often rely on standardized programs with limited personalization, particularly outside elite contexts. An autonomous exercise prescription agent can expand access to individualized programming for athletic, clinical, and general populations. The agent assesses individual characteristics, generates evidence-based programs aligned with specific goals, automatically progresses difficulty based on performance improvements, and provides detailed instructions through video demonstrations. Such agents extend benefits beyond elite athletics to clinical populations managing non-communicable diseases. Structured exercise interventions benefit patients with cardiovascular diseases, T2DM, chronic respiratory conditions, and musculoskeletal disorders [49, 50]. However, many patients lack access to specialized exercise professionals. An exercise prescription agent could partially address this gap for individuals who have obtained appropriate medical clearance, while supplementing rather than replacing qualified clinical oversight.

Having (i) established the conceptual foundation distinguishing AI agents from traditional AI applications and conversational tools, (ii) identifie the lack of sports science research in this domain, and (iii) outlined strategic implementation considerations, our review aimed to examine theoretical applications, technical architectures, and research priorities on the topic. For this review, an “AI agent” is defined as a system exhibiting the following architectural features: (1) continuous environmental monitoring, (2) autonomous reasoning capabilities enabled by large language models, (3) direct action execution through tool access, (4) outcome-based learning, and (5) coordination among multiple agents. This definition differentiates these agents from semi-autonomous, rule-based, or episodic recommendation systems currently prevalent in sports science. While AI applications in sports science are well-established, the deployment and study of such fully autonomous, multi-agent architectures remain limited. To address this gap, we synthesized foundational principles from computer science and analogous healthcare applications where similar architectures have been implemented, thereby constructing a theoretically grounded framework tailored for sports science. The subsequent sections proposed comprehensive frameworks for developing, validating, and implementing AI agent systems in sports science contexts. This framework prioritized modular development over premature integration. We examined training load management, exercise prescription, biomechanical analysis, nutrition optimization, sleep monitoring, injury prevention, mental skills training, and rehabilitation as priority domains for initial agent development. Multi-agent coordination protocols have received attention as essential infrastructure for subsequent integration. Throughout this analysis, we maintained a focus on human oversight requirements, ethical considerations, and validation methodologies necessary for responsible field advancement.

## 2 MATERIALS AND METHODS

### 2.1 Review Design and Rationale

Our literature search targeted two distinct areas. Core algorithmic concepts came from contemporary AI agent studies. Theoretical application workflows relied on established sports science research. Examples include training load management, nutrition, and sleep hygiene. This dual approach justifies the extensive reference list.

We employed a critical analytical approach to examine emerging technological applications. Direct empirical literature remains absent in this area. The following subsections describe the review design, search strategy, inclusion and exclusion criteria, analytical framework, and limitations in sequence. Traditional systematic review methodologies struggle with nascent, conceptually diffuse domains. PRISMA (for preferred reporting items for systematic reviews and meta-analyses) guidelines and meta-analytic synthesis require a corpus of comparable studies. That corpus does not yet exist for this topic.

The lack of specific peer-reviewed research on sports science AI agents required a comprehensive synthesis. We incorporated relevant concepts from multiple disciplines. Our narrative review methodology allowed a flexible combination of theoretical frameworks and technological specifications. We also integrated analogous applications from adjacent fields. We maintained analytical rigor through transparent documentation. This included explicit search terms, Boolean strategies, and inclusion criteria. We detail the iterative thematic framework in Section 2.4.

We adopted an interpretive stance focusing on conceptual innovation rather than exhaustive documentation. A simple summarization of existing AI applications in sports would provide limited value given the extensive prior coverage. Our analytical framework prioritized the transition from passive analytical tools to autonomous systems. We examined technological limits and possibilities relevant to sports science. We then synthesized implementation frameworks from successful technology adoption patterns. Finally, we proposed research agendas grounded in the identified knowledge gaps.

### 2.2 Literature Search Strategy

#### 2.2.1 Database Selection and Search Execution

We conducted searches across four electronic databases from August to October 2025. *PubMed/Medline* provides comprehensive biomedical and health sciences literature relevant to sports medicine, exercise physiology, and clinical applications. *Scopus* offers broader multidisciplinary indexing, including engineering and computer science publications. *Web of Science Core Collection* enables citation network analysis and interdisciplinary discovery. *Google Scholar* supplements academic databases with grey literature, conference proceedings, technical reports, and industry publications.

Each database serves a distinct purpose. *PubMed* contains clinical and physiological research on training adaptation, injury mechanisms, exercise prescription, and chronic disease management. *Scopus* identified computer science literature on AI agent architecture, multi-agent systems, autonomous decision-making frameworks, and LLM applications. *Web of Science* facilitated citation tracking of seminal papers on AI applications in sports. *Google Scholar* locates industry white papers, technical documentation, and emerging commercial applications.

#### 2.2.2 Search Terms and Boolean Strategies

Our search strategy evolved through several iterations. The initial exploratory searches employed the following: (artificial intelligence OR machine learning OR AI) AND (agent OR agents OR autonomous OR agentic) AND (sport OR athlete OR exercise OR training OR performance). These searches yielded minimal results specific to agent-based systems, confirming the hypothesized gaps in the literature.

We expanded our approaches to capture traditional AI applications by providing a contextual background: (artificial intelligence OR machine learning OR deep learning) AND (sport OR athlete OR exercise) AND (performance OR training OR injury OR prevention OR prediction). This broader strategy identified 9,745 articles across the databases. Additional targeted searches were conducted to examine specific domains such as [(“training load” OR periodization) AND (monitoring OR optimization) AND (AI OR “artificial intelligence” OR automated)], [(“exercise prescription” OR rehabilitation) AND (personalized OR individualized) AND (AI OR algorithm OR automated)], and [(“injury prevention” OR “injury prediction”) AND (AI OR “machine learning”)].

We searched the AI agent literature in computer science and healthcare to identify analogous applications using the following combinations: [(AI agent OR “multi-agent system” OR “autonomous agent”) AND (healthcare OR medical OR clinical)], [(“large language model” OR LLM OR GPT) AND (agent OR autonomous OR tool-use)].

Grey literature searches employed simplified terms as follows: “AI agents”, “sports science”, “agentic AI”, “athletic performance”, “autonomous AI”, and “training optimization”.

**FIG. 1:**
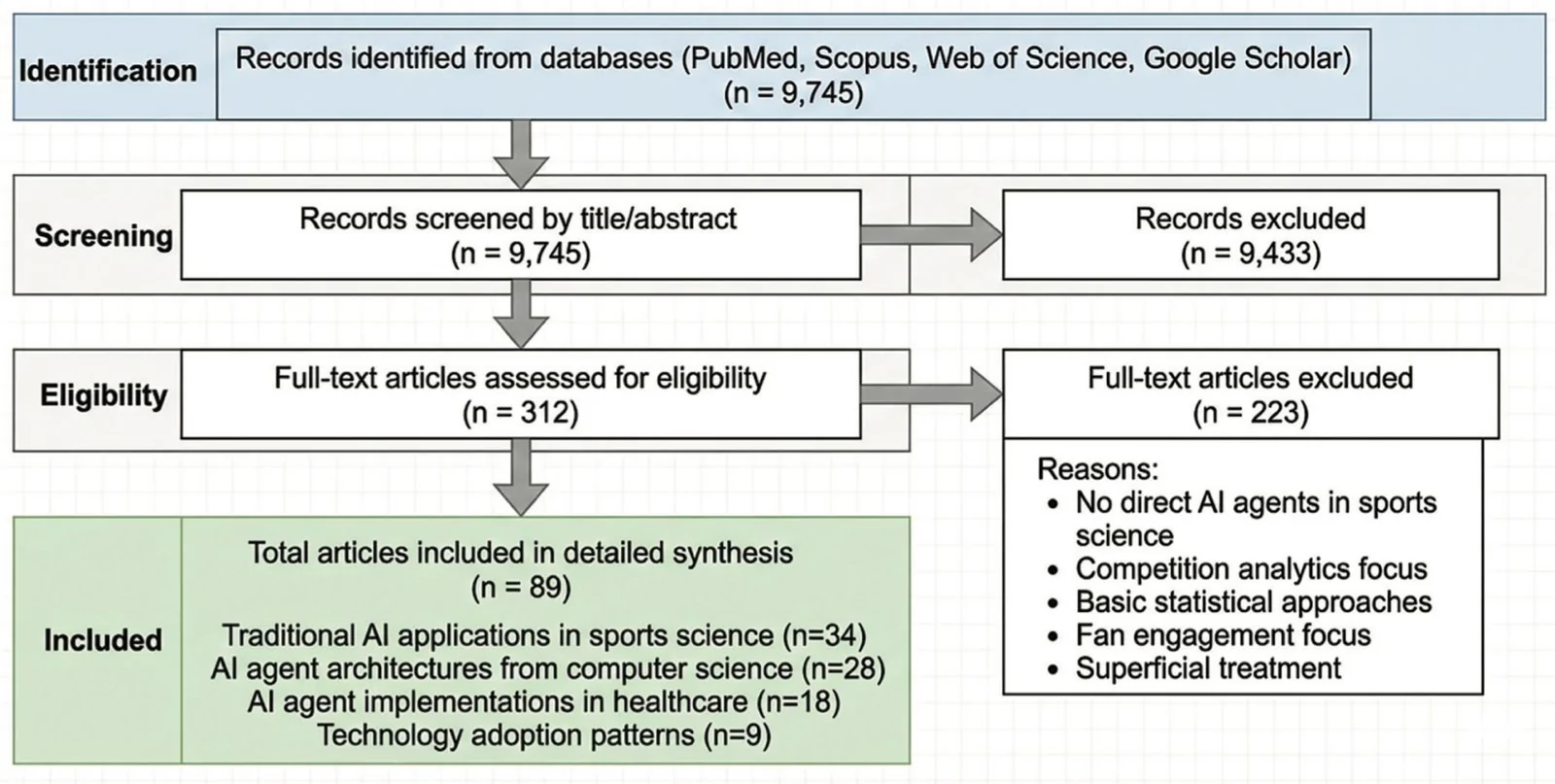
Literature Selection Flow Diagram: Artificial intelligence (AI) Agents in Sports science Review.

Database searches across *PubMed, Scopus, Web of Science*, and *Google Scholar* identified 9,745 records. After removing duplicates and applying inclusion criteria (Table 1), 89 articles were retained for synthesis. Specifically, the 9,745 records were first deduplicated across databases and screened by title and abstract (n = 312 advanced to full-text review); 223 records were subsequently excluded based on the criteria in Table 1, yielding 89 articles for synthesis, as detailed in Figure 1. Two independent reviewers (*ID* and *SB* in the authors’ list) conducted screening, with a third reviewer (*HBS* in the authors’ list) resolving disagreements.

**TABLE 1:** Inclusion and exclusion criteria.

Criterion category	Inclusion criteria	Exclusion criteria
**Study type**	Peer-reviewed articles; high-quality conference proceedings; technical reports with substantive content	Purely theoretical research without application pathways; commercial marketing materials; news articles; blog posts
**Subject matter**	Artificial intelligence (AI) applications in sports science; AI agent architectures from computer science; AI agent implementations in healthcare; technology adoption case studies; foundational AI concepts; autonomous systems design	Sports analytics without athlete performance focus; fan engagement applications; fantasy sports; competition outcome prediction without practical application; studies examining only basic statistical approaches
**Research design**	Any design examining AI applications, agent architectures, or technology adoption	Case studies lacking technical substance; opinion pieces without evidence base; purely speculative discussions
**Population**	Athletes of any age or level; sports science and strength and conditioning practitioners; healthcare practitioners included as secondary analogical sources only	Non-athlete populations unless providing direct analogical evidence for autonomous agent applications in health-related contexts (except healthcare providers as secondary sources)
**Outcome focus**	Training optimization; injury prevention/prediction; performance enhancement; recovery optimization; clinical applications with sports relevance	Competition analytics; fan engagement; commercial applications unrelated to athlete health/performance
**Language**	English-language publications	Non-English publications
**Publication date**	Primarily past ten years (2016–2025); seminal historical papers establishing foundational concepts included	Publications before 2005, except seminal foundational works, except seminal foundational works (except seminal foundational works)
**Literature source**	*PubMed/MedlinE*; *Scopus*; *Web of Science*; *Google Scholar*; expert recommendations; citation tracking	Conference proceedings without peer review (limited inclusion); grey literature without substantive technical content

#### 2.2.3 Citation Tracking

Beyond database searches, we employed citation tracking of key papers. Forward citation searches identified up-to-date studies citing foundational AI in sports publications. Backward citation analysis was used to examine the reference lists of relevant reviews. We consulted experts in sports science, AI research, and sports technology development to identify unpublished works and industry applications. Unpublished works and industry applications identified through expert consultation were used solely to map deployment contexts and technology landscapes, not as evidence of effectiveness.

### 2.3 Inclusion and Exclusion Criteria

Our inclusion criteria evolved iteratively as follows. Initially seeking research specifically examining fully autonomous, continuously operating AI agent systems in sports science and finding no eligible studies, we broadened the criteria to include traditional AI applications in sports, providing contextual background, computer science literature on AI agent architectures, healthcare AI agent applications offering analogous use cases, and technology adoption case studies illustrating successful modular implementation. We prioritized publications from the past ten years (i.e., 2016–2026), given the rapid advancement of AI, including seminal historical papers establishing foundational concepts. Language restrictions limited the inclusion of non-English publications. Peer-reviewed articles were prioritized, although we included high-quality conference proceedings and technical reports that provided essential unavailable information.

The exclusion criteria removed purely theoretical AI research without clear application pathways, commercial marketing materials lacking technical substance, news articles without peer review, and studies focusing exclusively on sports analytics or fan engagement rather than athlete performance and health.

The initial screening examined the titles and abstracts. Two reviewers (*ID* and *SB* in the authors’ list) independently assessed all records and resolved disagreements through discussion. Any persistent discrepancies were resolved by a third reviewer (*HBS* in the authors’ list). This resulted in 312 articles warranting full-text review. A detailed examination revealed that while no studies directly addressed AI agents in sports science, substantial literature existed on traditional AI applications, foundational agent concepts, and analogous implementations. We explicitly documented this absence as a primary finding.

The 312 articles were further categorized. We retained 89 articles for detailed synthesis after excluding studies focusing purely on competition analytics, examining only basic statistical approaches, addressing fan engagement rather than athlete performance, and providing superficial treatment without technical depth. The final inclusion resulted in 89 articles: 34 examined traditional AI applications in sports science, 28 described AI agent architectures from computer science, 18 documented AI agent implementations in healthcare, and nine analyzed technology adoption patterns.

Table 1 outlines the systematic standards applied to screen and select studies for the literature review on autonomous AI agents in sports science. The table is organized into eight criterion categories, each specifying what types of studies were included versus excluded from the analysis.

### 2.4 Analytical Framework

Our analytical framework rests on three premises that require explicit statements. First, technological capabilities demonstrated in one domain can be transferred to adjacent domains sharing similar characteristics when appropriate adaptations occur. Second, successful technology adoption follows predictable patterns, prioritizing focused applications before broader integration. Third, human expertise remains essential as the system “orchestrator”. While AI agents automate the linking of stakeholders (e.g., coaches, athletes, patients) and inform decision-making through continuous monitoring, the human practitioner must retain ultimate authority over complex, value-laden choices. We examined AI agent capabilities documented in computer science, assessed the alignment between these capabilities and sports science challenges, identified potential barriers and enablers for transferring agent technologies to athletic contexts, and developed implementation frameworks consistent with successful adoption patterns.

Theme development occurred iteratively through a repeated data review. Initial themes were deductively derived from the research questions. Subsequent inductive analysis identified additional themes, including critical distinctions between agents and conversational AI, human oversight as a non-negotiable requirement, notification systems as key differentiators, modular development as a strategic imperative, and equity considerations regarding supervised versus unsupervised access.

## 3 RESULTS

### 3.1 Literature Search Results

Table 2 presents the characteristics of the included literature, demonstrating the interdisciplinary synthesis required given the absence of direct sports science research on autonomous agents.

**TABLE 2:** Characteristics of included literature by domain and publication year.

Domain category	Number of articles, publication year range	Primary geographic origin	Key focus areas
**Traditional AI in sports science**	N = 34 2018–2025: Of all 34 traditional AI papers, 76% (n = 26) were published since 2020, 41% (n = 14) appearing in 2023–2025	North America: 41% Europe: 29% Asia: 21% Other: 9%	Injury prediction models using ML Performance analytics and outcome prediction Training load monitoring systems Biomechanical analysis through computer vision Tactical pattern recognition

**Computer science AI agents**	N = 28 2022–2025: 82% since 2023	North America: 39% Asia: 32% Europe: 25% Other: 4%	Agent architectures and reasoning mechanisms LLM-based autonomous systems Tool-use frameworks and function calling Multi-agent coordination protocols Explainable AI for agent systems

**Healthcare AI agent applications**	N = 18 2020–2025	Europe: 50% North America: 33% Asia: 17%	Patient monitoring and early warning systems Clinical decision support agents Chronic disease management automation Medication adherence tracking Care coordination systems Early clinical deterioration prediction using ensemble ML (Area Under the Receiver Operating Characteristic curve ^3^0.98)

**Technology adoption patterns**	N = 9 2015–2024	North America: 56% Europe: 33% Asia: 11%	Modular implementation strategies User acceptance factors and resistance patterns Integration challenges in complex systems Scaling approaches from pilot to production

AI: artificial intelligence. LLM: large language model. ML: machine learning.

The temporal distribution revealed important trends. Traditional AI in sports publications has clustered heavily in recent years (mainly after 2020), with 41% appearing in 2023–2025, reflecting the maturation of ML applications. The computer 2012 science AI agent literature showed an even more pronounced recent concentration, with 82% published since 2023, reflecting rapid growth in publications following the emergence of LLMs in 2023–2024. Healthcare implementations remained sparse, with only 18 studies identified despite extensive searching.

The geographical distribution showed a concentration in North America and Europe for traditional AI in sports (70%), reflecting the research infrastructure in these regions. Computer science agent research has demonstrated broader international representation, including substantial Asian contributions. Healthcare implementations appeared most frequently in European contexts, which may reflect greater integration of electronic health record infrastructure, though this interpretation requires cautious treatment given the aggregate nature of the geographic data.

### 3.2 Fundamental Distinctions: Traditional AI, Conversational AI, and Autonomous Agents

Traditional AI applications have achieved considerable success in sports science domains over the past decade (i.e., 2016–2026). ML models predict injury risk by analyzing training load patterns, biomechanical asymmetries, and wellness data [51, 52]. Computer vision systems automatically track player movements during competitions [53]. Predictive algorithms optimize the training periodization [54]. Wearable analytics processes physiological data to estimate recovery status [55]. These applications share a common operational architecture, fundamentally limiting their utility despite their technical sophistication.

Our synthesis identifies a critical architectural divergence. Traditional AI operates on a Batch Processing model where data is collected, stored, and processed at discrete intervals (e.g., post-session analysis). The system’s state remains static until a human queries it. Conversely, AI Agents operate on a Continuous Event-Loop architecture [22]. The agent maintains a persistent awareness of the athlete’s state, cycling through perception, reasoning, and action loops in real-time (milliseconds to seconds). This architectural shift moves the ‘bottleneck’ of the workflow from the human practitioner to the computational edge.

This passive architecture creates significant problems. Cognitive burden increases when staff members monitor multiple platforms, generating separate recommendations across domains. Time delays occur between data collection, analysis, and intervention implementation phases. Critical periods may span 24–48 hours from data generation until intervention occurs. Real-time intervention opportunities during training sessions are missed because the systems cannot process and respond instantaneously. The reactive nature of these approaches means that problems are identified after manifestation rather than through prevention via continuous monitoring and proactive adjustment. Information fragmentation across non-communicating systems forces the manual integration of training load, nutrition, recovery, psychology, and biomechanics insights [56].

The following theoretical scenario illustrates how autonomous agents could operate in principle. Elite endurance athletes complete intensive training to prepare for competitions. Daily sessions generate Global Positioning System (GPS) data that quantify distance and intensity [57]. HR monitors capture the cardiovascular responses and recovery patterns. Sleep tracking measures the duration and quality of sleep. Wellness questionnaires assess mood and perceived recovery status. Performance testing evaluates the physiological adaptations. Each data stream feeds into separate analytics platforms. The training load platform calculates acute-to-chronic workload ratios (ACWR), flagging elevated injury risk. Sleep analytics report poorquality scores. Wellness questionnaires revealed elevated fatigue ratings. Performance testing showed declining power outputs, suggesting inadequate adaptation.

A comprehensive assessment would recognize these converging signals as indicators of accumulated fatigue, requiring training reduction or recovery emphasis. However, these platforms operate independently. Unless a sports scientist manually reviews all systems, integrates information, recognizes the pattern, and implements changes, the athlete continues the prescribed training, potentially progressing to non-functional overreaching, illness, or injury [47]. Even when conscientious practitioners identify problems, implementation requires multiple steps: communicating with coaches, convincing them to modify their plans, ensuring athlete understanding and acceptance, and monitoring compliance. This manual process consumes time, during which conditions may deteriorate [58].

AI agents can transform this situation through autonomous operations and continuous adaptation. An agent monitoring the training load does not wait for scheduled analysis intervals. The agent continuously processes incoming data as devices transmit information. When patterns indicating an elevated risk emerge, the agent immediately detects them through persistent monitoring. Rather than generating passive dashboard alerts, the agent reasons about appropriate responses given the specific athlete context, training schedule, and concern severity. The agent accesses tools for implementing decisions, including modifying training prescriptions on connected platforms, sending stakeholder notifications through email or messaging, scheduling follow-up assessments, and documenting decisions with rationales [58, 59].

Crucially, agents learn from outcomes using feedback loops. When interventions successfully prevent injuries, the agent strengthens their confidence in similar responses for comparable situations. When injuries occur despite interventions, the agent analyzes which additional warning signs it might have detected earlier and adjusts future decision-making accordingly. When coaches override agent recommendations, the agent observes subsequent outcomes and recalibrates its understanding of acceptable versus unacceptable risk levels. Such learning could, in principle, occur without requiring explicit human reprogramming for each scenario, though the feasibility and reliability of autonomous learning in the noisy, high-variance environment of sports practice remain unvalidated and represent a primary target for future empirical research [60].

The technical architecture underlying this operational difference merits a detailed examination. Traditional AI systems employ taskspecific ML models trained on historical datasets. A random forest model might predict hamstring injury based on features including acute-chronic workload ratio, previous injury history, age, and sprint volume [61, 62]. This model exists as a static mathematical function. It receives inputs, applies the learned patterns, and produces outputs. The model cannot perceive environments beyond explicit inputs, formulate plans, execute actions through external interfaces, or learn from deployment outcomes without undergoing complete retraining.

Autonomous agents require persistent memory to track athlete adaptations over entire seasons. Current language models face strict context window limits. They cannot process months of raw multivariate data in a single reasoning step. The architecture must incorporate Retrieval-Augmented Generation. The system stores historical athlete data in vector databases. The agent retrieves only the most relevant historical context when making a new decision. This method prevents the reasoning engine from dropping critical temporal details.

Modern AI agents employ LLMs as reasoning engines, which offer broader cross-domain reasoning generalizability compared to taskspecific machine learning models [63, 64]. These foundation models, trained on vast text corpora, develop a broad understanding of language, concepts, and reasoning patterns. The agent uses language models to process natural language instructions, understand complex contexts involving multiple interacting factors, reason through multi-step problems requiring intermediate conclusions, generate contextually appropriate responses considering situational nuances, and adapt to novel situations that are not explicitly programmed.

The agent accesses specialized tools through function-calling mechanisms, transforming it from a passive analytical system into an active participant capable of perceiving situations, deciding on actions, and implementing interventions [65]. Function calling allows agents to retrieve information from databases or Application Programming Interfaces (APIs), send communications through email or messaging platforms, schedule appointments or assessments in calendar systems, modify training plans in connected programming platforms, and communicate with other agents using standardized protocols. Language models operate probabilistically rather than deterministically. This creates a technical execution risk. An agent might hallucinate a numerical parameter and attempt to adjust a training load by an unsafe magnitude. Tool execution cannot rely entirely on the language model’s raw output. The architecture requires a deterministic middleware layer. This software layer sits between the agent’s reasoning engine and the actual tool API. It sanitizes all function calls. It automatically blocks any action violating hard-coded physiological safety limits before execution occurs.

Multi-agent systems introduce additional sophistication through inter-agent communication, enabling coordination across specialized domains [16]. Individual agents focus on specific athlete management aspects, such as training load monitoring, nutrition optimization, sleep tracking, and injury risk assessment. These specialized agents share information through standardized protocols, coordinate recommendations to avoid conflicts, collectively solve problems that exceed the scope of a single agent, and negotiate priorities when multiple agents suggest competing interventions.

Coordination scenarios are considered. A TLMA identifies upcoming intensive training that requires enhanced recovery support. The TLMA would communicate this information to the sleep agent responsible for optimizing rest. The sleep agent would adjust recommendations, suggesting an earlier bedtime, cooler room temperature, and reduced screen exposure. It notifies a nutrition agent that increased training demands require adjusted meal timing to support overnight recovery. The nutrition agent would modify recommendations by increasing carbohydrate allocation at dinner and suggesting pre-bedtime snacks to support muscle protein synthesis during sleep.

This coordination occurs automatically through inter-agent communications. No human practitioners manually connect these insights. Agents recognize domain interactions and proactively share relevant information with other agents. However, suppose nutrition agent recommendations conflict with weight management goals. The athlete aims to reduce body fat, while the TLMA identifies the need for increased energy intake to support intensive training. These competing objectives require negotiations.

Agents may negotiate autonomously if conflicts fall within preset parameters. The nutrition agent proposed a compromise: increase energy intake by 10% rather than 20%, emphasizing nutrient timing around training to maximize performance support while minimizing fat gain. If a conflict exceeds the autonomous resolution parameters, the system escalates to human oversight. A sports scientist reviews the situation, considers additional contextual factors that agents cannot access, and makes final decisions by balancing competing objectives.

This architectural distinction has profound implications. Traditional AI provides decision support by presenting information for human interpretation. AI agents enable decision-making automation within defined parameters. Agents handle routine decisions independently, freeing practitioners to focus on complex cases that require human judgment. Ambiguous situations or those exceeding preset authority parameters escalate to human oversight rather than autonomous handling. This shift promises substantial benefits, including efficiency gains through automated routine monitoring and adjustment, true personalization through continuous adaptation to individual responses, reduced delays between problem detection and intervention, feasibility of 24/7 monitoring without corresponding staffing, and liberation of practitioner cognitive resources for complex cases [66].

However, automation introduces new risk. Agents may be susceptible to generating inappropriate recommendations when encountering situations outside their training distributions or involving atypical presentations. A lack of transparency in reasoning processes could obscure why specific recommendations were made. Over-reliance might erode human expertise if practitioners lose opportunities to develop clinical judgment. Malfunctioning agents can cause harm through incorrect interventions before humans [67].

### 3.3 Conversational AI Versus Autonomous Agents: Critical Distinctions

The proliferation of conversational AI tools has created substantial confusion regarding what constitutes an AI agent. Athletes, coaches, and sports scientists are increasingly experimenting with widely used LLM-based conversational interfaces for training advice and nutritional guidance [22, 24]. These tools generate impressively fluent responses to prompts. However, current versions differ fundamentally from AI agents, and this distinction has important implications.

ChatGPT and analogous conversational interfaces function primarily as interactive text-generation systems. While most-updated versions have introduced limited context-retention features, these remain fundamentally passive. The operational sequence relies on user input: The user provides data, and the system generates a response. Unlike an autonomous agent, such a system cannot independently verify whether an athlete followed a suggested program, cannot autonomously adjust recommendations based on real-time wearable data, and cannot coordinate proactively with other systems without explicit user prompting [68].

This fundamental lack of persistence creates significant limitations. For example, an athlete who queries a conversational interface on Monday and reports excessive fatigue on Friday initiates two independent interactions. The system retains no record of Monday’s recommendations, cannot observe how the athlete responded on Tuesday and Wednesday, and must rely entirely on the athlete accurately describing all relevant information in each query. This places an enormous burden on athletes to synthesize information, recognize patterns, and communicate all relevant contexts. Most athletes lack the expertise to do this effectively.

Research has documented substantial deficiencies in applying general-purpose conversational AI to specialized sports science tasks. Regarding exercise prescription, Dergaa et al. [8] identified that ChatGPT provided generic recommendations lacking individualization, failed to adequately account for medical contraindications, provided inappropriate progressions potentially leading to injury, and generated inconsistent advice when asked identical questions multiple times. For mental health assessment, ChatGPT demonstrated concerning limitations, including the inability to recognize serious psychiatric symptoms requiring immediate professional attention and the provision of generic coping strategies without appropriate tailoring [29]. Similar problems have emerged with nutrition counselling and resistance training prescriptions, including inadequate personalization, failure to consider medical dietary restrictions, and inconsistent recommendations [28, 30].

These limitations largely stem from fundamental design priorities rather than fixable technical deficiencies. Conversational AI serves as a general-purpose interface trained for helpfulness across countless topics, rather than as a specialized tool designed for athletic performance optimization. Systems are optimized to generate plausible-sounding responses that users find satisfactory. They do not optimize the provision of medically appropriate, individually tailored, evidence-based recommendations that account for complex contraindications and contextual factors.

Moreover, excessive reliance on AI-generated recommendations may impair cognitive development and clinical reasoning skills. Dergaa et al. [32] documented this concern by analyzing how practitioners interact with AI outputs. When experts routinely defer to AI recommendations rather than engaging in independent reasoning, their clinical judgment skills may deteriorate. This is particularly concerning for developing practitioners who need extensive deliberate practice to build expertise. If novice sports scientists habitually consult conversational AI rather than working through problems independently with mentorship, they may fail to develop the deep understanding necessary for expert practice. Habitual reliance on AI-generated outputs may attenuate the development of independent clinical reasoning, reducing rather than augmenting professional competence [32].

This concern assumes particular significance given the fundamental AI limitations persisting regardless of technical sophistication. AI lacks genuine clinical judgment, which involves the integration of subtle contextual cues that are often difficult to articulate explicitly, recognizing atypical presentations deviating from standard patterns, applying ethical reasoning to ambiguous situations with competing considerations, and adapting flexibly to rapidly changing circumstances based on tacit knowledge [33, 34]. These capabilities emerge from extensive deliberate practice, embodied experience working with athletes, supervised learning through mentorship, and reflective analysis. No amount of text-training data can replicate this developmental process.

**FIG. 2:**
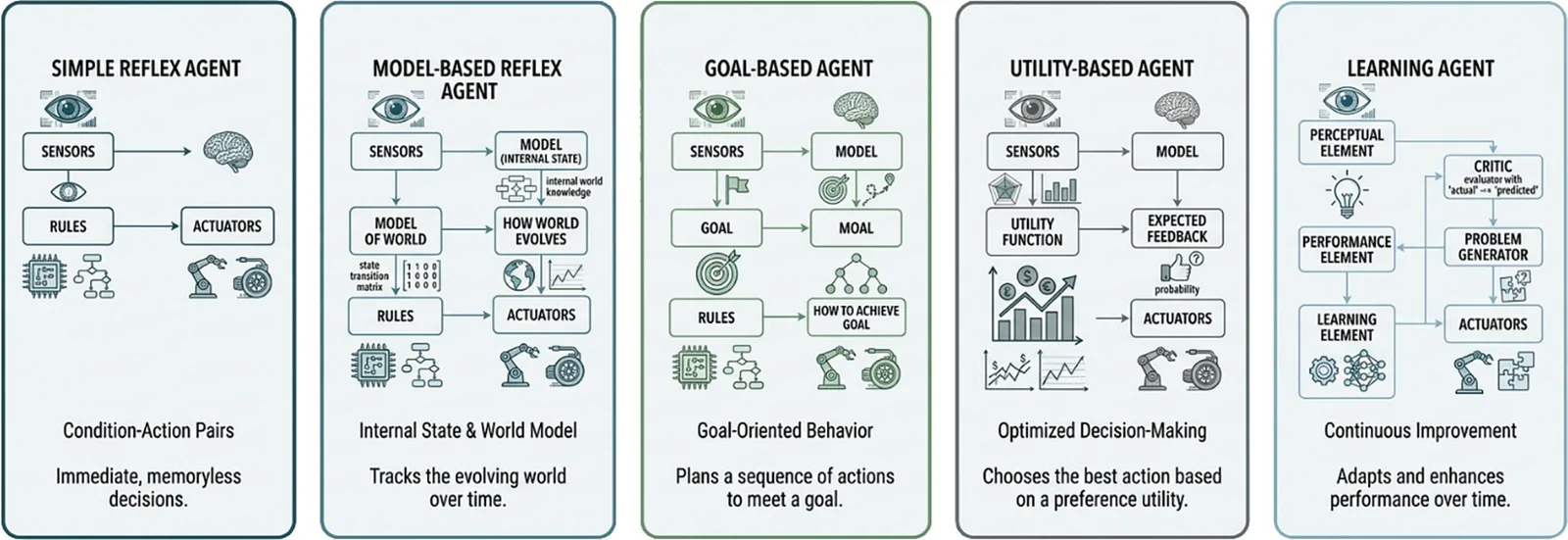
Taxonomy of Artificial Intelligence Agent Architectures.

Following the taxonomy of Russell and Norvig [15], Figure 2 presents five principal AI agent architectures, organized by increasing complexity and their capacity to interpret the environment, pursue goals, and adapt through learning. A *Simple Reflex Agent* responds only to the current percept using predefined condition–action rules and operates without memory. A *Model-Based Reflex Agent* introduces an internal state and a world model, enabling it to track how the environment evolves. A *Goal-Based Agent* adds explicit goals and uses planning or search strategies to determine the actions needed to reach its objectives. A *Utility-Based Agent* goes further by applying a utility function to evaluate possible future outcomes, selecting actions that maximize expected utility while balancing goals and risks. Finally, a *Learning Agent* incorporates learning mechanisms and a critic, allowing it to update and refine its performance element over time based on feedback.

Table 3 summarizes the key distinctions between conversational AI tools and autonomous AI agents, clarifying the fundamental architectural and operational differences.

**TABLE 3:** Key distinctions between conversational artificial intelligence (AI) tools and autonomous AI agents.

Characteristic	Conversational AI (e.g., Current Chat Generative Pre-trained Transformer (GPT), Claude)	Autonomous AI agents (Theoretical sports science applications)
**Memory & continuity**	No memory between conversations; each interaction independent	Continuous awareness of athlete status over time; persistent memory of interactions and outcomes
**Environmental perception**	Relies entirely on user-provided text descriptions in each conversation	Would access wearable devices, training logs, and assessment results through Application Programming Interface connections
**Action capability**	Generates only text responses requiring manual user implementation	Is proposed to modify training plans, send notifications, schedule assessments, and coordinate with systems
**Response pattern**	Reactive: responds only when user initiates queries	Proposed as proactive: would initiate communication when monitoring thresholds are crossed
**Goal-directed behavior**	Aims to satisfy individual queries without longterm objectives	Pursues defined objectives (e.g.; optimize performance while minimizing injury) across extended timeframes
**Learning mechanism**	Static: same responses regardless of previous outcomes	Proposed as dynamic: would observe intervention results and adjust future decision-making
**Personalization depth**	Surface-level: based only on information in current conversation	Deep: based on comprehensive longitudinal data about individual responses, preferences, constraints
**Coordination capability**	Isolated: cannot communicate with other systems	Integrated: coordinates with specialized agents managing different domains through standardized protocols
**Monitoring scope**	None: cannot observe anything between conversations	Continuous: 24/7 monitoring of relevant data streams from multiple sources
**Intervention timing**	Delayed: user must recognize need, formulate query, implement suggestions manually	Immediate: automatic detection and response within minutes or seconds of pattern recognition
**Safety mechanisms**	Limited: relies on user judgment identifying inappropriate advice	Robust: built-in constraints, escalation protocols, human oversight triggers, comprehensive audit trails
**Validation status**	Research shows limitations for sports science tasks	No validation studies exist; all applications theoretical requiring empirical testing
**Use case suitability**	General question-answering, information retrieval, idea generation, educational purposes	Specialized athlete management requiring continuous monitoring, autonomous decisions, coordinated interventions
**Development pathway**	Current technology already available and widely accessible	Future versions of foundation models (e.g.; successors to GPT-4) may provide infrastructure enabling agent development through external tool connections

Future LLM versions may provide the infrastructure for building AI agents. Anticipated successors to GPT-4 and similar next-generation foundation models could serve as reasoning engines that developers connect to external tools, databases, and monitoring systems [26, 27]. The language model provides intelligence and reasoning capabilities. External connections provide perception and action capabilities. Together, these components enable autonomous operations. A developer could take a future language model’s reasoning capability and build an autonomous TLMA by connecting it to wearable device APIs for data ingestion, training management platforms for prescription modification, communication systems for notifications, and logging databases for outcome tracking. The language model reasons about which actions to take. External connections enable environmental perception and decision execution.

Current (i.e., April 2026) versions of conversational AI lack these external connections. They functioned purely as conversational interfaces. Users cannot i) connect the current ChatGPT to athletes’ wearable devices for continuous training load monitoring, ii) grant ChatGPT permission to modify training schedules automatically; and iii) enable ChatGPT to send emails to coaches when concerning patterns emerge. These capabilities require agent architectures with tool access, not just conversational interfaces.

The sports science community should clearly understand this distinction. Experimenting with ChatGPT or similar tools for occasional training questions poses a minimal risk and may provide helpful general information. However, relying on conversational AI as a primary athlete management tool creates substantial safety concerns and is likely to produce suboptimal outcomes. Athletes lack expertise in providing a comprehensive context, recognizing subtle warning signs, and synthesizing information across domains. Conversational AI lacks the perception, memory, and action capabilities required for effective athlete management.

The path forward involves using conversational AI appropriately for actual capabilities while developing truly autonomous agents for applications that require continuous monitoring and proactive intervention. These technologies serve complementary purposes rather than overlapping purposes. Understanding these distinctions prevents both the overestimation of current conversational AI capabilities and the underestimation of what properly designed autonomous agents could achieve.

**FIG. 3:**
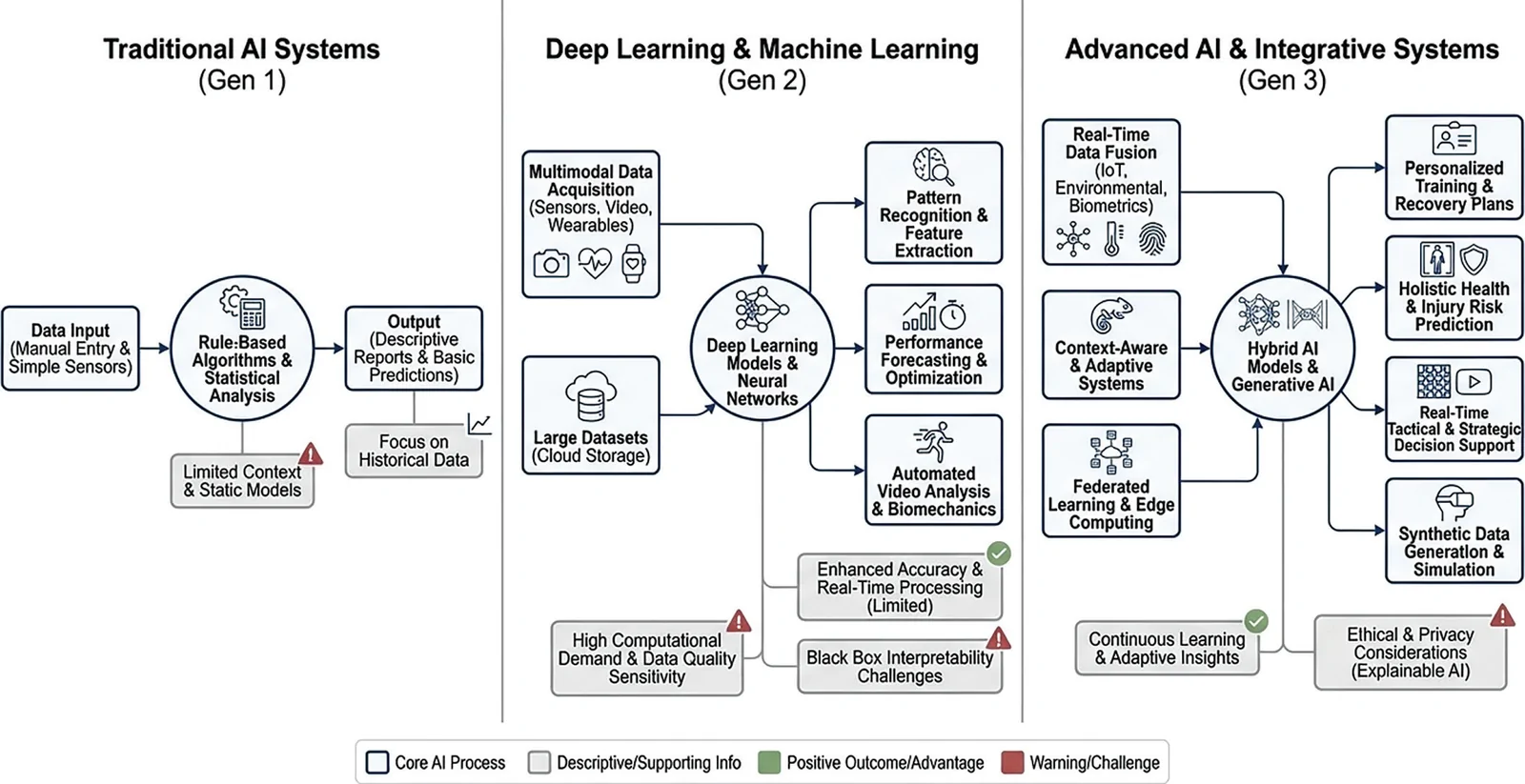
Fundamental Architectural and Operational Distinctions between Traditional Artificial Intelligence (AI), Conversational AI, and autonomous AI agents in Sports Science. Gen: Generation. IoT: Internet of things.

Figure 3 delineates the architectural and operational distinctions among three generations of AI applications pertinent to sports autonomous AI agents in Sports Science. Gen: Generation. IoT: Internet of things. science: (i) Traditional AI is characterized by passive, discrete, and human-dependent workflows, marked by time delays and fragmented information; (ii) Conversational AI (e.g., ChatGPT) exemplifies reactive, text-based interfaces that lack persistent memory, environmental perception, autonomous action, and clinical judgment, and (iii) Autonomous AI agents represent continuous, proactive, and adaptive systems capable of real-time monitoring, reasoning (LLM-driven), function calling for intervention, and dynamic learning from outcomes, while also introducing new risks. The bottom panels summarize the *Current Use* (appropriate vs. risky for athlete management) and the *Future Potential & Path Forward* (transformative automation necessitating human oversight and ethical consideration).

### 3.4 Modular Implementation Framework: Strategic Phasing for Practical Adoption

The pathway from current traditional AI applications to comprehensive multi-agent systems requires strategic planning rather than rapid attempts at full integration. Technology adoption research across multiple industries reveals consistent patterns in which successful implementations begin with focused, high-value applications before expanding to broader integration [69, 70]. Understanding these adoption patterns is essential for the strategic development of sports science AI agents [44, 69–71].

*Apple*’s *iPod* (2001) demonstrated this principle by establishing the technical and commercial infrastructure that enabled the *iPhone*’s integration of telephony, computing, and media six years later [72, 73]. Critically, each phase delivered standalone value regardless of future developments. Sports science AI agent development should follow the same logic: each agent must solve a measurable problem in its domain before integration is attempted. *Apple* to introduce the *iPhone* in 2007, integrating telephony, mobile computing, media playback, and Internet connectivity [72]. The *iPhone* leveraged the technical infrastructure developed for the *iPod*, including user interface principles, manufacturing expertise, and supply chain relationships. However, *Apple* did not attempt this integration immediately after. Six years elapsed between the *iPod* introduction and the *iPhone* launch. This timeline allowed for systematic development, validation of individual technologies, refinement based on user feedback, and accumulation of resources supporting integration that is more ambitious.

Each phase delivered a standalone value, justifying its existence independent of future developments. *iPod* users gained benefits immediately from better portable music, regardless of whether *Apple* developed additional products. *iPhone* users who never owned *iPods* still received tremendous value. This characteristic is crucial. Each implementation phase must solve real problems and deliver measurable benefits on its own merits, not merely serve as a stepping-stone toward eventual comprehensive integration.

### 3.5 Proposed Three-Phase Implementation Framework

We propose a three-phase implementation framework for AI agent development in sports science, extending over a speculative horizon of approximately 5 to 10 years, contingent on validation outcomes, regulatory approvals where applicable, and adoption rates.

#### 3.5.1 Phase 1: Specialized Single-Domain Agents (Years 1–3)

The initial phase focuses on developing and validating autonomous agents that operate within clearly defined domains. Each agent addresses a specific aspect of athlete management. Each agent would operate within its specialized domain while accessing relevant data from other domains to inform context-appropriate decisions in a *Phase 1* implementation. *Phase 1* priorities include training load management, exercise prescription, biomechanical analysis, nutrition optimization, sleep and recovery monitoring, injury prevention, mental skills training, and rehabilitation progression. Each application domain receives comprehensive development attention, including technical implementation, validation studies, user interface design, human oversight protocols, and documentation supporting widespread adoption.

Strategic decisions involve selecting which applications to prioritize for initial development. Four criteria should guide this selection process. First, data availability determines feasibility. Domains with established data collection infrastructure through existing wearable devices or assessment protocols are more immediately accessible than those requiring new measurement systems. Second, the existence of evidence-based guidelines is important because agents require validated decision rules. Domains with clear consensus on best practices are easier to program than those with substantial controversy or limited research. Third, outcome metric clarity enables validation of results. Domains with objective and measurable outcomes allow evaluation of agent performance compared to traditional approaches. Fourth, potential impact magnitude justifies development investment. Domains affecting large populations or addressing critical health and safety issues warrant prioritization.

Implementation requires clarity about data access patterns. Agents must access information beyond their primary domain to make contextually appropriate decisions. A training load agent examining elevated heart rate during a session gains critical insight by accessing recent sleep quality data, which may reveal inadequate recovery explaining the physiological response. Similarly, a nutrition agent calculating fueling requirements needs current training load data to match energy provision to expenditure. This cross-domain data access occurs through standardized queries where agents retrieve information from other measurement systems or databases. Agents interpret this contextual data within their specialized decision-making frameworks without delegating authority or sharing control. The architecture maintains clear domain boundaries while acknowledging that isolated systems lacking contextual awareness produce suboptimal recommendations.

#### 3.5.2 Phase 2: Multi-Agent Coordination (Years 3–5)

Once individual specialized agents demonstrate validated performance within their domains, *Phase 2* would advance from passive crossdomain data access to active multi-agent coordination. *Phase 1* agents access information through unidirectional queries, retrieving data to contextualize decisions within their specialized frameworks. *In Phase 2*, agents would engage in qualitatively different interactions: bidirectional communication, negotiation when recommendations conflict, synchronized interventions, and collective optimization, all requiring formal coordination protocols not established in current *Phase 1* architectures. A training load agent and sleep agent might collaboratively determine whether poor sleep justifies reducing training intensity or whether maintaining training load while addressing sleep factors directly produces better outcomes. This represents fundamentally different functionality requiring formal coordination protocols, conflict resolution mechanisms, and shared decisionmaking frameworks absent in *Phase 1*.

#### 3.5.3 Phase 3: Integrated Systems with Comprehensive Reasoning (Years 5–10+)

*Phase 3* represents a long-term vision that requires substantial advances beyond current technological capabilities. Rather than multiple specialized agents coordinating through protocols, *Phase 3* envisions integrated systems with sophisticated reasoning capabilities that simultaneously handle multiple domains. These systems would understand the complex relationships between training, nutrition, recovery, psychology, and biomechanics. They would make holistic recommendations by considering all factors simultaneously rather than negotiating between specialized agents. They would handle conflicting priorities intelligently through nuanced reasoning about individual athletes’ goals, circumstances, and constraints.

However, this vision remains speculative and unproven. Although current AI agent technology is impressive, we believe that it lacks the reasoning sophistication required for truly integrated athletic performance management. The gap between coordinated specialized agents (*Phase 2*) and comprehensive integrated systems (*Phase 3*) may be substantial. *Phase 3* may require technological advances that are not yet on the horizon. Alternatively, *Phase 2* coordination might prove sufficiently effective, and *Phase 3* integration might provide minimal additional benefits relative to its complexity.

We include *Phase 3* in our framework for completeness but emphasize that immediate priorities rest firmly in *Phase 1*. The field currently lacks even basic single-domain agents that are validated for sports science applications. The development, testing, and refinement of these foundational applications will require years of effort. *Phase 2* coordination can begin once multiple *Phase 1* agents demonstrate validated performance. *Phase 3* integration remains a distant possibility rather than a near-term goal.

Table 4 summarizes the three-phase implementation framework, showing the progression from focused applications to eventual integration.

**TABLE 4:** Three-phase framework for artificial intelligence agent implementation in sports science.

Phase Timeline	Primary focus Technical complexity	Key theoretical applications
**Phase 1: specialized single-domain agents** **Years 1–3**	Develop and validate independent agents operating within clearly defined domains **Moderate**: single agents with tool access and autonomous decision-making within preset parameters	Training load management Exercise prescription Biomechanical analysis Nutrition optimization Sleep and recovery monitoring Injury prevention Mental skills training Rehabilitation progression

**Phase 2: multi-agent coordination** **Years 3–5 (after Phase 1 validation)**	Develop protocols enabling specialized agents to coordinate across domains **High**: inter-agent communication protocols, conflict resolution mechanisms, priority negotiation systems, escalation pathways	Training-nutrition coordination Load-recovery synchronization Injury prevention-performance optimization balance Integrated athlete monitoring systems Cross-domain information sharing

**Phase 3: integrated comprehensive systems** **Years 5–10+ (speculative; dependent on technological advances)**	Develop unified systems with sophisticated reasoning handling multiple domains simultaneously **Very high**: advanced reasoning across domains, complex trade-off analysis, nuanced understanding of interactions, sophisticated long-term planning capabilities	Comprehensive athlete management Holistic performance optimization Integrated health and performance monitoring Adaptive long-term development planning Complex trade-off reasoning


**Phase Timeline**	**Proposed validation approach**	**Projected benefits (pending validation)**

**Phase 1: specialized single-domain agents** **Years 1–3**	Domain-specific outcomes (injury rates, performance metrics, adherence, user satisfaction) compared to traditional approaches through randomized controlled trials and implementation studies	Projected immediate practical value, pending validation, pending validation. in each domain Reduced practitioner cognitive burden 24/7 monitoring capability Faster intervention response times Technical and user experience foundations for subsequent phases

**Phase 2: multi-agent coordination** **Years 3–5 (after Phase 1 validation)**	Integrated outcomes (overall athlete health, performance across multiple dimensions, system efficiency) and coordination quality metrics through comparative effectiveness studies	Holistic athlete management Reduced conflicts between domains Optimized recommendations considering interactions Improved decision quality through information sharing Reduced redundancy in monitoring

**Phase 3: integrated comprehensive systems** **Years 5–10+ (speculative; dependent on technological advances)**	Whole-athlete outcomes, longitudinal development trajectories, long-term health and performance optimization through prospective cohort studies and pragmatic trials	Seamless integration across all domains Sophisticated reasoning about complex trade-offs Reduced system complexity from user perspective Potentially superior decision quality (Benefits uncertain; may not exceed Phase 2 coordination substantially)

This phased approach offers multiple advantages over immediate comprehensive integration. The technical complexity remains manageable in each phase. Single-domain agents involve substantially simpler architectures than multi-agent coordination, which, in turn, proves far simpler than fully integrated systems. Validation is feasible when evaluating focused applications with clear outcome measures. Validating the performance of a comprehensive integrated system across multiple domains simultaneously proves far more challenging than validating the performance of individual specialized agents. User acceptance increases when practitioners clearly understand what each agent does and retains control over complex decisions. Comprehensive integrated systems functioning as opaque algorithms are likely to encounter resistance from practitioners who are uncomfortable delegating complex decisions to systems they cannot fully understand. Resource requirements are scaled appropriately to the benefits delivered. Each phase requires substantial investment but delivers corresponding value, justifying the costs.

The timeline presented assumes reasonable progress with validation studies, regulatory approvals, where applicable, and user acceptance. Delays can substantially extend these timeframes. Conversely, rapid technological advances or urgent needs driven by specific events can accelerate development. The framework provides a roadmap rather than a rigid schedule.

**FIG. 4:**
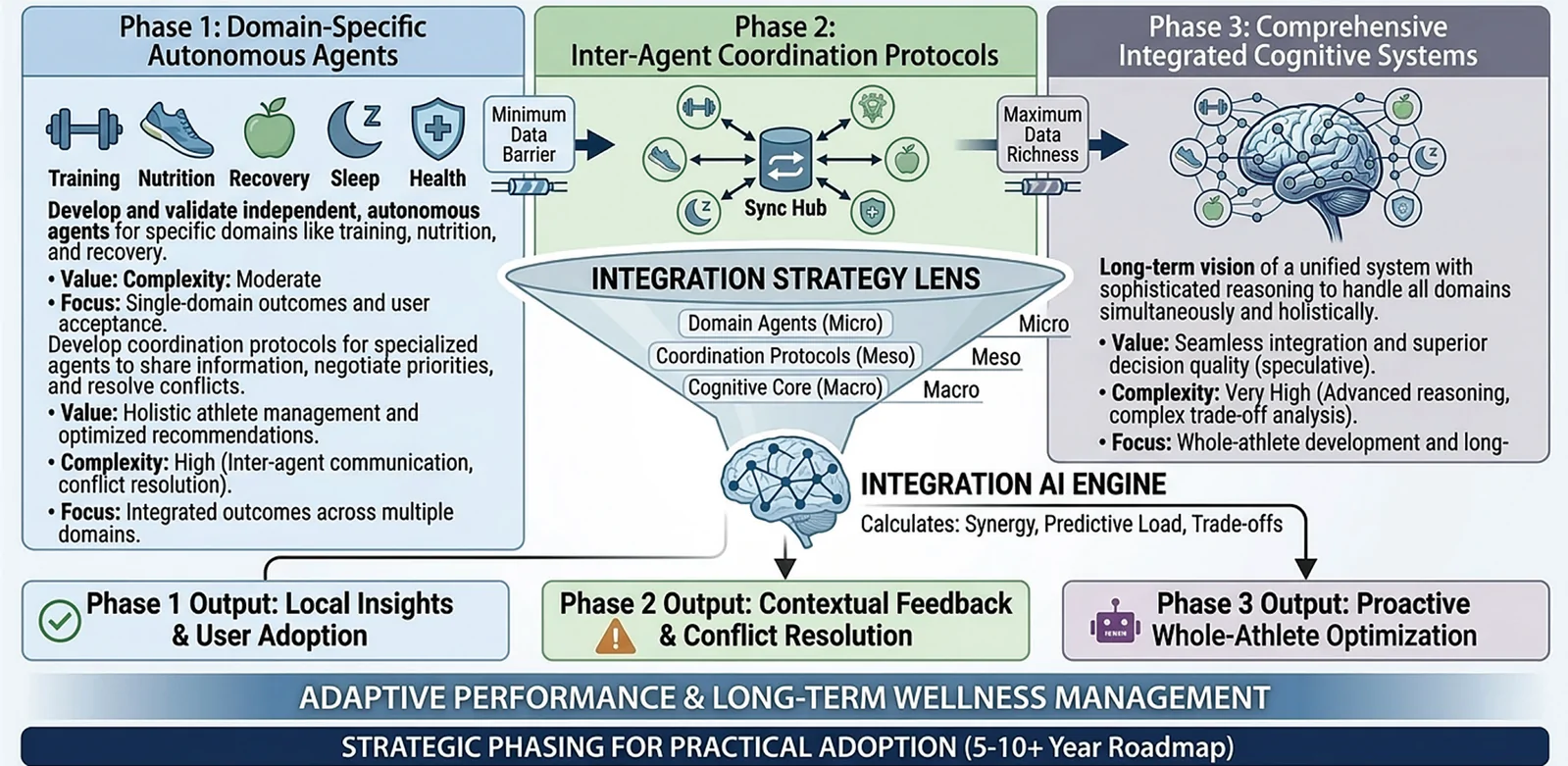
Strategic Phasing of Artificial Intelligence (AI) Integration in Sports Science: A Multi-Stage Roadmap.

Figure 4 illustrates a three-phase implementation roadmap delineating the necessary progression for AI agents within the domain of sports science. The framework initiates with single-domain agents, characterized by low complexity and high immediate value. It subsequently progresses to multi-agent coordination systems, which are marked by high complexity and a holistic approach to athlete management. The roadmap culminates in integrated comprehensive systems, distinguished by very high complexity, superior decision quality, and long-term optimization. This progression indicates a sustained commitment over a period of 5 to 10 or more years.

### 3.6 Theoretical Application Domain 1: Training Load Management Agent

Training load management represents the optimal initial application of AI agent development in sports science. This section examines the specific implementation details, validation requirements, and practical considerations for deploying an autonomous TLMA. All content represents theoretical proposals that require empirical validation before implementation.

#### 3.6.1 Foundational Monitoring Tools: From Subjective Indices to AI Integration

Before examining how AI agents process data, we must establish what data they receive. Training load monitoring evolved from expensive laboratory-based assessments requiring specialized equipment to practical field methods accessible in any setting. This democratization proves essential for AI agent deployment because autonomous systems require systematic data collection, but that data need not originate from costly technology. Understanding the full spectrum of available monitoring tools reveals how agents can function effectively across diverse resource contexts.

##### 3.6.1.1 Subjective Load Quantification: The Foundation of Accessible Monitoring

The rating of perceived exertion (RPE) scale quantifies athletes’ subjective training intensity through standardized numerical ratings. Borg developed the original 6–20 scale with anchoring to HR responses, where RPE 6 approximates 60 beats per minute (bpm) and RPE 20 approximates 200 bpm [73]. The modified Category-Ratio-10 scale provides finer discrimination at higher intensities. These scales require no equipment beyond the athlete’s self-awareness and demonstrate strong validity across diverse populations and training modalities.

Foster et al. [74] extended RPE’s utility by developing session-RPE (sRPE) methodology. This approach multiplies perceived intensity by session duration in minutes, yielding a comprehensive internal load metric. An athlete rating a 90-minute session as RPE 7 produces sRPE of 630 arbitrary units. The elegance lies in integration: sRPE captures both intensity and volume in a single value reflecting the athlete’s internal response. Daily sRPE values accumulate to weekly totals, enabling longitudinal tracking without specialized equipment. Validation studies demonstrate strong correlations with objective markers including blood lactate accumulation, heart rate responses, and subsequent performance changes [74, 75].

Raw weekly load totals provide limited insight without contextual analysis. Training monotony, calculated as mean weekly load divided by standard deviation of daily loads, quantifies day-to-day variability [74]. Low monotony reflects high daily variation characteristic of periodized programs that alternate hard and easy sessions. High monotony indicates minimal daily variation, a pattern that increases injury and illness risk through inadequate stimulus variation and insufficient recovery opportunities. Training strain multiplies weekly load by monotony, yielding a composite measure that integrates volume, intensity, and variation [74]. Foster’s seminal work with speed skaters demonstrated that periods of high strain preceded overtraining symptoms and illness episodes, validating these calculations as meaningful fatigue indicators.

The Acute to Chronic Workload Ratio (ACWR) extends load monitoring by comparing recent loading (typically 7-day rolling average) to longer-term loading (typically 28-day rolling average) [44]. This ratio quantifies training progression rate. Values between 0.8–1.3 represent appropriate progression where athletes adapt to gradually increasing demands. Ratios exceeding 1.5 indicate dangerous load spikes associated with elevated injury risk, though recent research highlights important limitations of fixed thresholds [44, 76–78]. The fundamental insight transcends specific cutpoints: rapid increases in training stress, regardless of absolute magnitude, create injury vulnerability. AI agents must interpret ACWR contextually rather than applying universal thresholds, as discussed in *Section 3.6.1.3*.

Moving beyond training load to broader wellness assessment, the Hooper Index provides rapid daily monitoring of recovery status [79]. Athletes rate four parameters on 7-point scales each morning: sleep quality, fatigue level, general muscle soreness, and stress level. Lower scores indicate better status. The entire assessment requires approximately 30 seconds, making daily completion feasible without disrupting athlete routines. Persistent elevations across multiple days warn of inadequate recovery before performance decrements become apparent, enabling proactive intervention rather than reactive damage control.

Sleep quality specifically warrants dedicated assessment given its fundamental role in recovery processes. The Pittsburgh Sleep Quality Index (PSQI) quantifies seven components: subjective sleep quality, sleep latency, sleep duration, sleep efficiency, sleep disturbances, use of sleeping medication, and daytime dysfunction [80]. The global PSQI score distinguishes good sleepers (≤ 5) from poor sleepers (> 5). While the full PSQI requires several minutes to complete, abbreviated versions provide sufficient temporal resolution for most training contexts.

These subjective tools share a critical advantage: minimal implementation barriers. A smartphone application collecting versions maintain validity for athletic populations while reducing completion burden. Weekly administration post-training RPE, morning Hooper Index values, and weekly PSQI scores provides sufficient systematic data for autonomous AI agent operation. No wearable devices, laboratory equipment, or specialized facilities are required. This accessibility proves transformative. Athletes training in rural areas, developing nations or resource-constrained clubs can access evidencebased monitoring frameworks previously available only to professional organizations with substantial budgets. The agents receive less rich data compared to comprehensive sensor deployments, affecting decision precision, but systematic subjective monitoring vastly exceeds purely intuition-based programming.

##### 3.6.1.2 Heart Rate-Based Monitoring: Bridging Subjective and Objective Assessment

While subjective measures provide accessible load quantification, HR monitoring adds objective physiological data. The technology spectrum ranges from free smartphone applications to research-grade chest strap systems, accommodating diverse implementation contexts. HR metrics complement rather than replace subjective assessment, with integration of both approaches yielding superior monitoring compared to either alone [81].

Resting heart rate (RHR) provides a simple yet informative daily metric. Measurement occurs immediately upon morning waking, before rising from bed, ensuring standardized conditions minimally influenced by activity or circadian variation. Well-trained athletes demonstrate progressive RHR reductions during appropriate training progression, reflecting positive cardiac adaptation and improved autonomic balance [82]. These adaptations typically unfold across weeks to months rather than days.

Conversely, acute RHR elevations signal physiological stress. Increases of 5–7 bpm above individual baseline, sustained across multiple consecutive days, indicate sympathetic nervous system overdrive characteristic of overreaching or early overtraining [83]. This marker provides sensitive early warning before performance decrements, motivation loss, or mood disturbances manifest. The practical value lies in timing: RHR elevation appears early in the fatigue accumulation cascade, enabling proactive load adjustment when reduction prevents breakdown rather than merely responds to established overtraining. Modern smartphone applications measure RHR through camera-based photo-plethysmography, eliminating equipment costs while maintaining adequate accuracy for trend analysis. Athletes place their fingertip over the camera lens for 30–60 seconds, with algorithms extracting heart rate from subtle color changes reflecting blood flow pulsations.

Beyond resting measurements, training HR analysis examines intensity distribution and cardiovascular responses during sessions. HR zone training distributes training intensity across physiological domains, though multiple zone classification systems exist. The traditional five-zone model defines zones based on percentages of maximum HR or HR reserve calculated via the Karvonen formula [84]. *Zone 1* (recovery, < 60% HR maximal (HRmax)) elicits minimal training stress. *Zone 2* (aerobic base, 60–70% HRmax) develops aerobic capacity through high-volume low-intensity work. *Zone 3* (tempo, 70–80% HRmax) represents moderate intensity often termed “no man’s land” in polarized training models. *Zone 4* (lactate threshold, 80–90% HRmax) improves sustainable high-intensity performance. *Zone 5* (maximal oxygen uptake (${\dot{\text{V}}\text{O}}_{2\max}$), > 90% HRmax) develops maximal aerobic power through interval training.

Training intensity distribution analysis examines time spent in each zone across days, weeks, or mesocycles. Polarized training models emphasize extensive low-intensity work (*Zones 1–2*, approximately 75–80% of total training time) with limited high-intensity intervals (*Zones 4–5*, approximately 15–20% of training time), deliberately minimizing moderate intensity (*Zone 3*) [85]. This approach contrasts with threshold-focused programs concentrating work in *Zone 3–4*. The optimal distribution varies by sport, athlete development level, and training phase. AI agents can track compliance with prescribed distribution patterns, detecting drift toward excessive moderate-intensity work that often occurs when athletes train by “feel” without systematic monitoring.

HRV quantifies beat-to-beat fluctuations in cardiac rhythm, reflecting autonomic nervous system balance [86]. Higher HRV indicates greater parasympathetic (recovery-promoting) activity and better stress resilience. HRV typically increases during appropriate training progression and decreases during overreaching, illness, or psychological stress. Morning HRV measurement requires 2–5 minutes using smartphone applications or chest strap monitors. The metric shows substantial day-to-day fluctuation, necessitating rolling averages (typically 7-day) for meaningful interpretation. HRV analysis proves particularly valuable when integrated with other metrics: concordant changes (elevated RHR plus decreased HRV plus poor Hooper scores) provide strong evidence of inadequate recovery, while discordant patterns (one metric aberrant, others normal) may reflect measurement error or transient factors requiring continued monitoring rather than immediate intervention.

HR drift during prolonged steady-state sessions offers additional insight. Cardiovascular drift describes the phenomenon where HR gradually increases during extended exercise at constant power output or pace, reflecting fluid loss, thermoregulatory demands, and progressive cardiovascular strain [87]. Excessive drift (> 5% HR increase during a steady 60-minute run) indicates incomplete recovery, heat stress, or inadequate fueling. AI agents can detect abnormal drift patterns suggesting athletes should terminate sessions early rather than completing planned durations under excessive physiological stress.

The equipment accessibility for HR monitoring creates implementation flexibility. Free smartphone applications provide RHR and HRV measurement without any additional hardware. Bluetooth chest strap monitors ($30–80) improve accuracy during training sessions. Research-grade systems ($200–500) enable detailed zone analysis and drift quantification. This tiered structure allows progressive technology adoption as resources permit, with each tier adding data richness rather than replacing previous approaches.

##### 3.6.1.3 Periodization Frameworks: Contextualizing Load Metrics

The metrics described above acquire meaning only within periodization context. Identical workload values or ratio calculations represent entirely different scenarios depending on where athletes reside in their training cycle. AI agents require periodization awareness to avoid inappropriate interventions that misinterpret intentional load manipulations as problematic deviations.

Periodization organizes training into hierarchical time structures with distinct objectives at each level. Microcycles, typically spanning one week, establish the fundamental repeating unit [88]. Daily training sessions within microcycles follow planned sequences: high-intensity sessions interspersed with recovery sessions, technical work alternating with physical loading, sport-specific training balanced with supplementary conditioning. The weekly structure repeats with progressive overload through the training block.

Mesocycles, spanning 3–6 weeks, group microcycles pursuing common objectives [88]. A preparatory mesocycle emphasizes general physical preparation with high volume and moderate intensity, building the physiological foundation for subsequent training. A strength mesocycle reduces endurance volume while increasing resistance-training loads. A competition mesocycle maintains fitness while managing fatigue through reduced volume and increased technical/tactical emphasis. Each mesocycle concludes with a recovery week featuring substantially reduced load before transitioning to the next phase.

Macrocycles encompass entire competitive seasons or annual training plans, organizing mesocycles into coherent progressions [88]. Traditional periodization models move from general preparation (high volume, low intensity, low specificity) through specific preparation (moderate volume, high intensity, high specificity) to competition phases (low volume, high intensity, maximum specificity) and concluding with transition periods (minimal structured training, active recovery). Block periodization models, advocated by Issurin [89], concentrate on developing specific abilities within shorter blocks through highly focused loading, then sequence these blocks to produce peak performance at target competitions.

This hierarchical organization creates profound implications for load monitoring interpretation. Consider ACWR values. A ratio of 0.6 during the first week of a planned taper represents appropriate load reduction preparing for competition. The same 0.6 ratio during early-season base building indicates insufficient training stimulus, potentially from athlete illness, excessive caution, or programming error. Without periodization context, an AI agent might incorrectly flag the taper scenario as problematic undertraining or miss the basebuilding scenario’s training deficiency.

Similarly, training monotony carries phase-dependent interpretation. High monotony (low day-to-day variation) during competition phases where athletes perform similar race-pace sessions repeatedly may prove acceptable or even intentional. Identical monotony during preparatory phases should trigger concern because varied stimuli promote broader adaptations. An appropriately trained agent recognizes that monotony thresholds require adjustment based on mesocycle objectives.

Weekly load progressions follow phase-specific patterns. Basebuilding phases employ gradual linear progressions (approximately 5–10% weekly increases) sustained across multiple weeks. Strength phases may feature more aggressive loading (10–15% increases) for shorter durations before planned recovery. Taper phases deliberately reduce load by 40–60% across 1–3 weeks. Competition phases maintain consistent moderate loads between events. Transition phases feature minimal structured loading. AI agents must recognize these patterns as intentional rather than concerning deviations from stable training.

The practical implementation requires programming agents with periodization awareness. Training plans uploaded to the system specify current microcycle, mesocycle, and macrocycle phases along with associated objectives. The agent references this contextual information when evaluating metrics. A load spike receives different interpretation during a shock microcycle designed to provoke adaptation versus a recovery microcycle intended for restoration. Expected ACWR ranges, acceptable monotony values, and appropriate weekly progressions all adjust based on current training phase. This contextual sensitivity distinguishes intelligent adaptive systems from simple threshold-based alarms.

##### 3.6.1.4 Tiered Implementation: Democratizing AI-Guided Training Load Management

The monitoring tools and frameworks described above support diverse implementation approaches accommodating different resource availability. This tiered structure prevents technology access from becoming a barrier to evidence-based training management.

*Tier 1* implementations, requiring minimal financial investment (free to $50), rely exclusively on subjective measures collected via smartphone applications. Athletes complete post-training RPE ratings, morning Hooper Index assessments, and weekly PSQI questionnaires through simple mobile interfaces. Applications transmit this data to cloud-based AI agents via standard internet connectivity. The agent receives daily sRPE values; wellness scores, and sleep quality ratings. From these inputs alone, the system calculates weekly loads, monotony, strain, and ACWR values. It compares current patterns to expected periodization progressions and generates training recommendations. Decision precision operates with wider confidence intervals compared to sensor-rich implementations, leading to more conservative load adjustments and lower autonomy in decision-making. However, systematic subjective monitoring with AI interpretation vastly exceeds purely intuition-based programming common in resource-limited settings.

*Tier 2* implementations add heart rate monitoring ($50–500 total investment). Athletes acquire basic chest strap monitors or use smartphone HRV applications for morning RHR and HRV measurement. Training sessions incorporate HR zone tracking when possible, though not all sessions require this level of monitoring. The agent now integrates subjective load perception with objective physiological responses. Discordance detection becomes possible: unusually high RPE relative to HR-indicated intensity suggests illness onset, psychological stress, or sleep deprivation affecting perceived effort. Conversely, blunted RPE despite elevated HR may indicate motivation deficits or underreporting requiring athlete education. This crossvalidation improves data quality and enables more precise recommendations.

*Tier 3* implementations incorporate comprehensive wearable technology (estimated $500–5,000+ investment), including GPS units, accelerometers, force plates, and biochemical monitoring [90]. The agent receives multidimensional input enabling more precise and potentially more autonomous decision support, appropriate for elite environments where marginal gains carry competitive value. e.g., ground contact time, stride characteristics, asymmetries), and additional physiological data.

This tiered approach addresses health equity concerns while maintaining scientific rigor. A community-running club in rural Kenya using *Tier 1* implementation receives autonomous load management recommendations based on systematic RPE collection via smartphones. A university program employing *Tier 2* implementation integrates HR data with subjective measures. An elite professional team deploying *Tier 3* implementation accesses comprehensive multidimensional analysis. All three benefit from AI-guided training management superior to purely coach-intuited programming. Data richness differs; affecting recommendation precision and autonomy levels, but the fundamental agent architecture remains identical across tiers.

The agent adjusts its operational parameters based on available data. With sparse *Tier 1* inputs, the system widens safety margins, requires human confirmation for more interventions, and presents recommendations with appropriate uncertainty quantification. With rich *Tier 3* inputs, the system narrows safety margins, operates more autonomously within preset boundaries, and provides precise quantitative guidance. This adaptive conservatism prevents the agent from overreaching its epistemic foundations.

**FIG. 5:**
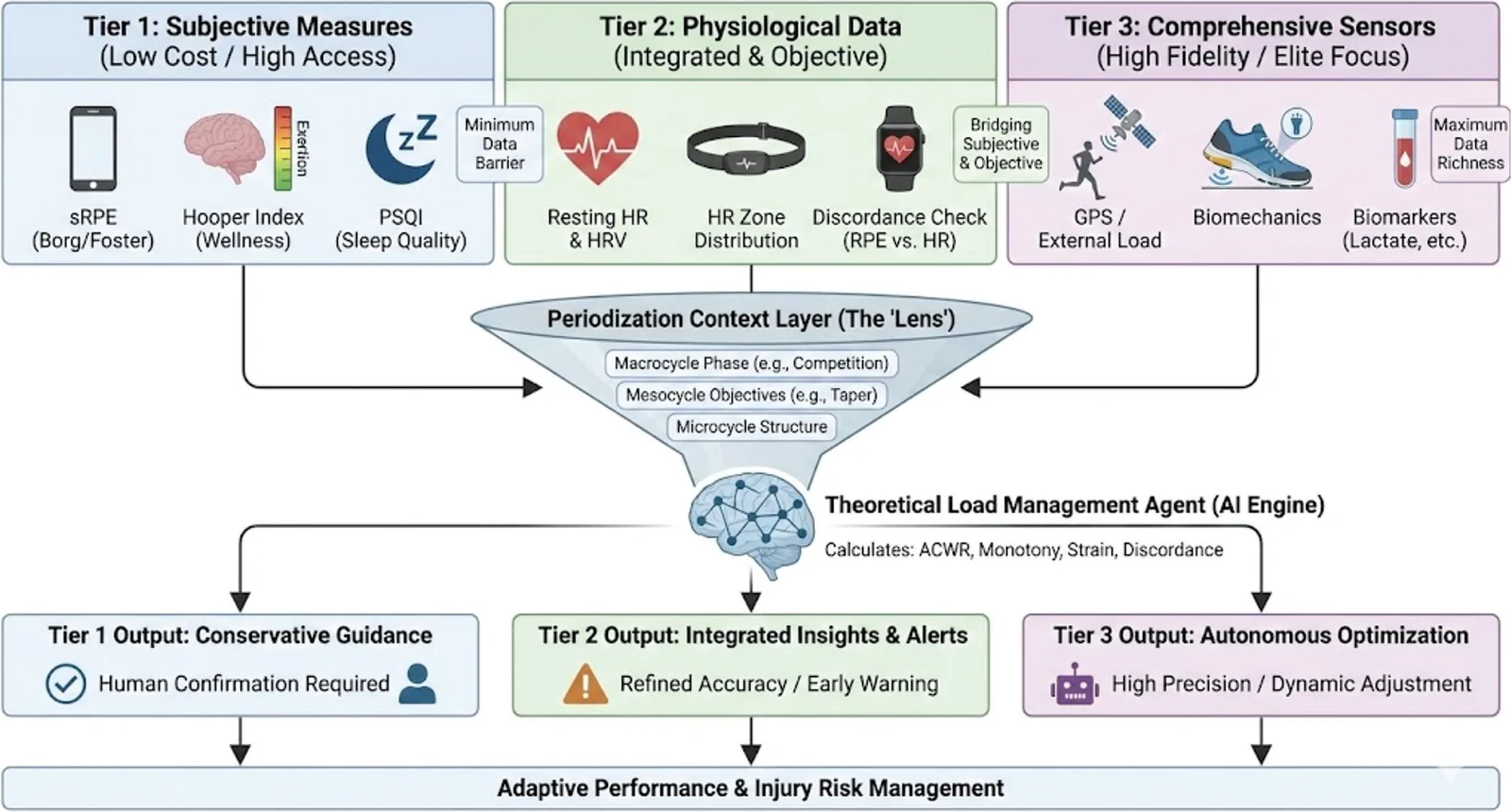
Tiered Framework for Artificial Intelligence (AI)-Driven Training Load Monitoring. ACWR: Acute Chronic Workload Ratio; GPS: Global Positioning System; HR: Heart Rate; HRV: Heart Rate Variability; PSQI: Pittsburgh Sleep Quality Index; RPE: Rating of Perceived Exertion; sRPE: Session Rating of Perceived Exertion.

Implementation tier selection depends on organizational resources, athlete populations, and performance objectives. Community health and fitness contexts benefit substantially from *Tier 1* systems that systematize monitoring without financial barriers. Developmental athletic programs leverage *Tier 2* implementations balancing cost with enhanced data quality. Elite performance environments justify *Tier 3* investments where marginal performance gains from optimized loading carry substantial competitive and financial value. The critical insight is that meaningful AI-guided load management remains accessible across the complete resource spectrum, from zero-budget community programs to well-funded professional organizations. The integration of these multi-level data streams into a unified processing architecture is synthesized in Figure 5, illustrating how AI agents adapt their operational precision based on available resource tiers.

#### 3.6.2 Core Functionality and Operational Workflow

A theoretical TLMA would operate through continuous monitoring cycles integrated with the existing wearable device infrastructure. The agent receives data streams from GPS units, HR monitors, accelerometers, and subjective wellness questionnaires as athletes complete training sessions. Modern wearable devices typically transmit data in real-time or near-real-time through wireless connections to cloud platforms. The agent accesses these data streams through application programming interfaces provided by device manufacturers or third-party data aggregation services.

Upon receiving new data, the agent calculates relevant metrics established in the sports science literature as indicators of training stress and recovery status. The acute training load represents the average daily load over the past seven days, typically using exponentially weighted moving averages, giving greater weight to recent sessions. The chronic training load represents the average daily load over the past 28 days, again using exponentially weighted calculations. The acute-chronic workload ratio divides the acute load by the chronic load, producing a metric indicating whether the current training exceeds or falls below the athlete’s established baseline [91, 92].

Research has established that acute-chronic workload ratios between 0.8 and 1.3 represent optimal ranges for adaptation, with minimal injury risk [93]. Ratios below 0.8 suggest undertraining, where athletes receive insufficient stimulus for continued adaptation. Ratios above 1.5 indicate dangerous spikes in training load associated with substantially elevated injury risk, particularly for soft-tissue injuries [94]. Ratios between 1.3 and 1.5 represent moderate concern, requiring monitoring and potential intervention depending on other factors.

The agent also calculated training monotony (average weekly load divided by standard deviation of daily loads) and training strain (total weekly load multiplied by monotony) [74]. High monotony indicates repetitive training without sufficient variation, which may increase the risk of injury and produce staleness. High strain combines a high absolute load with high monotony, representing particularly concerning patterns.

Beyond these established metrics, the agent incorporates individual athlete baselines and response patterns. Athletes vary substantially in their training load tolerance. What proves optimal for one individual may represent overtraining for another. The agent tracks each athlete’s historical responses to various load patterns, identifying individual thresholds and early warning signs specific to that person.

#### 3.6.3 Hierarchical Decision-Making Structure

When the agent would detect concerning patterns, it initiates decisionmaking processes to determine appropriate responses. This process follows a hierarchical structure, moving from minor adjustments to major interventions based on severity.

– **Level 1:** Low Concern (Monitoring Without Intervention)
  An ACWR between 1.3 and 1.5 with no other concerning indicators would trigger enhanced monitoring without immediate intervention. The agent flags the athlete for closer attention and increases the wellness questionnaire prompt frequency to detect the development of any subjective symptoms. The agent would send informational notifications to the coaching staff noting the elevated ratio but not recommending specific action beyond continued monitoring.
– **Level 2:** Moderate Concern (Minor Automatic Adjustments)
  An ACWR exceeding 1.5 or a ratio between 1.3 and 1.5 combined with declining wellness scores or reduced HRV would trigger minor automatic adjustments. The agent modifies the next scheduled training session within the preset parameters. For example, if the planned session involved high-intensity intervals, the agent might reduce the interval count from 8 to 6, decrease the intensity from 95% to 90% of the maximum, or increase the recovery duration between intervals from 2 to 3 minutes.

These modifications would occur automatically without requiring human approval because they fall within the authority parameters established during agent configuration. The agent would simultaneously send detailed notifications to the athlete explaining the modification with specific reference to monitoring data: “Your training has been adjusted for today. Your workload ratio is currently 1.6, which indicates that you require additional recovery. Today’s interval session will include 6 intervals instead of 8, allowing your body to adapt to recent training stress”.

The coaching staff would receive parallel notifications with more technical details: “Training modification implemented for Athlete X. ACWR ratio: 1.6 (threshold: 1.5). HRV declined by 15% from the baseline. Wellness fatigue rating: 6/10. Adjustment: The interval count was reduced from 8 to 6, and the intensity was maintained at 90%. Rationale: Moderate overload indicators suggest need for load reduction while maintaining intensity to preserve adaptation stimulus.”

– **Level 3:** High Concern (Major Interventions and Escalation)
  An ACWR exceeding 2.0, or a ratio above 1.5 persisting for more than three consecutive days despite previous adjustments, or any ratio elevation combined with severely declining wellness scores and physiological markers would trigger major interventions and escalation to human oversight. The agent would replace scheduled training with active recovery or complete rest, depending on the severity. This would alert both the coaching staff and medical personnel. Immediate medical evaluation should be scheduled to rule out illness, injury, or other underlying issues. All decisions and monitoring data are documented in the athlete’s medical record for clinician review.

The agent would not return the athlete to normal training autonomously after high concern escalations. Human clearance is required. A sports medicine physician or sports scientist must review the athlete’s status, conduct necessary examinations or testing, and explicitly authorize return to training before the agent resumes normal programming.

#### 3.6.4 Learning and Adaptation Mechanisms

A critical feature distinguishing AI agents from static algorithms is outcome-based learning. The theoretical TLMA would continuously observe the relationship between its decisions and subsequent athlete outcomes. When an athlete experiences an injury, the agent conducts a retrospective analysis to examine whether any detectable warning signs preceded the injury that the agent missed or weighted insufficiently. When an athlete remains healthy and performs well despite elevated load metrics, the agent notes this positive outcome and adjusts its understanding of the individual’s tolerance.

This learning process occurs through several mechanisms. First, the agent would maintain detailed logs of all decisions, including monitoring data at decision time, the decision made, the reasoning process followed, and subsequent outcomes over the following days and weeks. Second, the agent periodically analyzes these logs to identify patterns. ML algorithms can detect correlations between early warning signs and subsequent injuries that may not match conventional wisdom. Third, the agent adjusts the decision-making weights based on the observed patterns. If HRV decline proves more predictive of injury than wellness questionnaire scores for a particular athlete, the agent would give HRV greater weight in future decisions for that individual.

However, this learning must occur within careful constraints to prevent harmful adaptations. The agent cannot autonomously modify core safety thresholds. Established parameters, such as never allowing ACWRs above 2.0, would remain fixed. The agent can adjust the weights of various indicators within safe ranges but cannot override the fundamental safety limits. All learning adaptations are logged for periodic human review. Sports scientists would examine learning patterns quarterly to identify concerning trends or inappropriate adjustments.

#### 3.6.5 Proposed Validation Approach

Before deploying a TLMA in operational settings, comprehensive validation through multiple study designs is necessary. We propose a three-stage validation pathway that progresses from controlled studies to real-world implementation.

– **Stage 1:** Retrospective Validation
  Researchers would train the agent using historical data from athletic populations, where outcomes are already known. The agent would receive training load and wellness data from past seasons but not injury outcome information. The agent would make decisions about when it would have intervened. Researchers would then compare agent decisions to actual injury occurrences. The metrics would include sensitivity (i.e., proportion of injuries the agent would have prevented through timely intervention), specificity (i.e., proportion of healthy athletes who would not have received unnecessary interventions), positive (i.e., proportion of agent interventions that would have prevented actual injuries), and negative (i.e., proportion of athletes flagged as safe who actually remained healthy) predictive values.
  This retrospective approach provides initial evidence regarding the performance of agents without compromising athlete safety. Limitations include the inability to determine whether agent interventions would have actually prevented injuries (since interventions did not occur) and potential differences between retrospective decisionmaking and real-time operations.
– **Stage 2:** Prospective Observational Study
  The agent operates in real-time, monitoring athletes and generating recommendations. However, the implementation of these recommendations remains at the discretion of the human. Coaches and sports scientists would receive agent suggestions but decide independently whether to follow them. Researchers tracked the concordance between agent recommendations and human decisions, injury rates when humans followed versus ignored agent advice, and acceptability and usability metrics from practitioners.
  This observational design provides evidence of the clinical validity of agent recommendations while maintaining complete human control. This allows the identification of situations in which the agent performs well or poorly. This reveals user acceptance issues that require interface or communication improvements. Limitations include confounding (e.g., humans might ignore agent recommendations for valid reasons the agent does not understand), and inability to definitively establish causation (e.g., did following agent advice prevent injury, or were those athletes already at lower risk?), and selection bias (e.g., coaches might be more likely to follow agent recommendations for athletes they already perceived as high risk).
– **Stage 3:** Randomized Controlled Trial
  Athletes or teams will be randomly assigned to TLMA (intervention) or traditional practitioner-managed training load (control). In the intervention group, the agent operated with authority, implementing automatic adjustments within preset parameters while escalating major decisions to human oversight. In the control group, practitioners managed the training load using traditional approaches with access to the same monitoring data but no agent recommendations. The primary outcomes included injury rates, training availability (proportion of scheduled training sessions completed), and performance measures. Secondary outcomes included practitioner time burden, athlete satisfaction, and cost-effectiveness.
  Rigorous validation is necessary. Traditional parallel-group Randomized Controlled Trials face limitations here. They struggle to evaluate autonomous agents because the algorithm’s decision process adapts dynamically. Empirical validation in sports science requires Algorithm-Adaptive Trial Designs. Researchers should utilize Micro-Randomized Trials and Sequential Multiple Assignment Randomized Trials. These frameworks evaluate dynamic, just-in-time adaptive interventions in real-world environments. Single-subject trial methodologies are also crucial. They confirm the personalized accuracy of specialized agents. This step must occur before deployment across wider team populations.
  All three-validation stages contributed essential information. Retrospective studies have provided initial safety and feasibility data. Observational studies reveal real-world performance and identify the challenges of implementation. RCTs establish definitive evidence of effectiveness, justifying widespread adoption.

#### 3.6.6 Practical Implementation Considerations

Deploying a TLMA in operational settings requires attention to several practical issues beyond technical functionality.

First, integration with existing workflows is crucial for acceptance. The agent must complement, rather than disrupt, established routines. If sports scientists currently review athlete-monitoring data each morning at 8 AM, the agent should deliver its reports and recommendations around that time rather than requiring practitioners to check updates constantly. If coaches prefer to receive summaries via email rather than mobile app notifications, the system should accommodate these preferences.

Second, transparency in decision-making builds trust and enables appropriate human oversight. When the agent implements training modifications, explanations should include specific data driving the decision, the reasoning process followed the range of actions considered, and why the chosen action proved optimal. Practitioners need sufficient information to evaluate whether agent decisions make sense, given their understanding of the athlete and the situation.

Third, the override mechanisms must be readily accessible. Humans must be able to disagree with agent recommendations and easily implement alternative decisions. The agent should request reasoning when practitioners override its suggestions, allowing the system to learn from these disagreements, but should not create barriers preventing overrides. The agent serves as a decision-support tool, not a dictator.

Fourth, the agent must be configurable to accommodate different coaching philosophies and athlete population characteristics. Some coaches prefer conservative approaches, prioritizing injury prevention even at a modest performance cost. Others accept a higher injury risk to maximize performance gains. The agent should allow practitioners to adjust their risk tolerance parameters within safe ranges. Youth athletes require different thresholds than elite professional athletes do. The agent must consider the developmental stage, training age, and performance level.

Fifth, data quality and reliability substantially affect agent performance. Wearable devices occasionally malfunction, producing spurious data. Athletes occasionally forget to wear the devices or wear them incorrectly. Subjective wellness questionnaires are prone to response bias. Agents require robust methods for detecting and addressing data quality issues. It should flag suspicious data for human review rather than making potentially harmful decisions based on unreliable data.

**FIG. 6:**
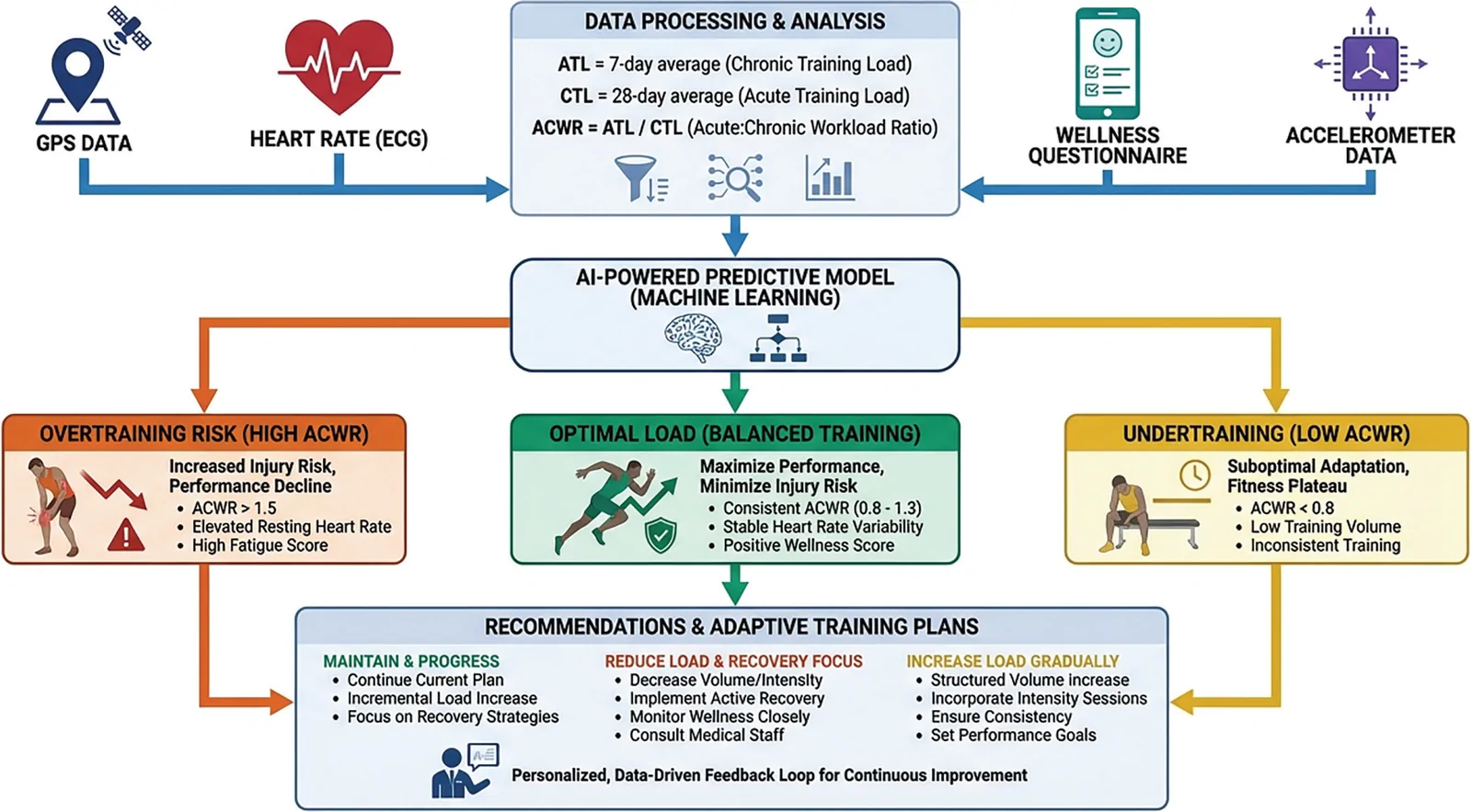
Artificial Intelligence (AI) Powered Training Load Management Agent for Athletes. ACWR: Acute Chronic Workload Ratio; ATL: Acute Training Load; CTL: Chronic Training Load; GPS: Global Positioning System.

Figure 6 outlines the operational workflow and hierarchical decision-making structure of a theoretical AI agent designed for managing athlete-training load. The process begins with data input from wearable devices (e.g., GPS, HR monitors, accelerometers) and subjective wellness questionnaires. This data is then processed and analyzed by the agent to calculate key sports science metrics such as acute training load over 7 days, chronic training load over 28 days, and the ACWR (ACWR = acute training load / chronic training load), along with training monotony and training strain. Based on these calculations, the agent employs a hierarchical decision-making process: *Level 1* (ACWR 1.3–1.5) triggers enhanced monitoring and informational notifications to coaching staff; *Level 2* (ACWR > 1.5) leads to minor automatic training adjustments (e.g., reduced interval count) and detailed notifications to athletes and staff; and *Level 3* (ACWR > 2.0 or persistent high risk) initiates major interventions like active recovery or rest, escalating to coaching and medical staff for mandatory review. The AI agent continuously learns and adapts by analyzing the correlation between its interventions and athlete health and performance outcomes, though core safety thresholds (e.g., ACWR never exceeding 2.0) remain fixed. Validation of this agent involves a threestage pathway: retrospective validation using historical data, a prospective observational study where humans decide on recommendations, and a randomized controlled trial comparing agent-managed and practitioner-managed groups.

### 3.7 Theoretical Application Domain 2: Exercise Prescription Agent

Exercise prescription is the second priority domain for the development of specialized AI agents. Unlike training load management, which focuses primarily on elite athletic populations, exercise prescription agents would extend benefits across athletic, clinical, and general populations [8]. This section examines the implementation details specific to exercise prescription while, noting how the requirements differ from those of training load management. All the content represents theoretical proposals that require empirical validation.

#### 3.7.1 Target Population Diversity and Adaptation Requirements

Exercise prescription agents must accommodate extraordinarily diverse populations, ranging from elite athletes seeking performance optimization to sedentary individuals beginning exercise programs to clinical patients managing chronic diseases. This diversity creates challenges that are absent in training load management, where the target population (i.e., athletes) shares common characteristics.

Three representative use cases illustrating diversity are considered. First, a collegiate basketball player seeks supplementary strength training to improve their vertical jump height. This athlete would possess high baseline fitness, access to professional-quality equipment, familiarity with complex exercises, no significant medical limitations, and performance enhancement as the primary goal. Second, a 55-year-old office worker with prediabetes seeks to improve metabolic health and prevent progression to T2DM. This individual has low baseline fitness, limited equipment access (perhaps a basic home gym), minimal exercise experience, medical contraindications requiring modified programming, and disease prevention as the primary goal. Third, a 72-year-old recovering from hip replacement surgery requires rehabilitation exercises to restore function. This patient has compromised baseline capacity, specific therapeutic equipment requirements, very limited exercise tolerance, strict medical protocols governing progression, and functional restoration as the primary goal.

A single agent must appropriately address all these scenarios. This requires sophisticated assessment processes, extensive exercise libraries with multiple difficulty progressions, decision rules that account for medical contraindications and adaptation algorithms that are responsive to individual goals and constraints.

#### 3.7.2 Comprehensive Assessment and Individualization

The theoretical exercise prescription agent would begin each engagement with a structured assessment gathering information across multiple domains.

Medical history assessment would identify contraindications and precautions through questionnaires addressing cardiovascular disease, diabetes mellitus, respiratory conditions, musculoskeletal injuries or surgeries, neurological conditions, pregnancy, medications affecting exercise responses, and any symptoms suggesting undiagnosed conditions. For certain high-risk conditions, medical clearance is required before proceeding with exercise prescription. The agent could not provide cleared-to-exercise authorization independently but could identify situations where physician consultation was necessary before beginning exercise programs.

Fitness assessment establishes the baseline capacity across relevant dimensions. Cardiovascular fitness assessment may use submaximal testing protocols to predict ${\dot{\text{V}}\text{O}}_{2\max}$ from HR responses to standardized work rates [73]. For individuals unable to perform standard tests due to health limitations, the agent would use simpler alternatives, such as the 6-minute walk test or self-reported activity tolerance [95]. Muscular fitness assessment evaluates strength and endurance through standardized tests appropriate for each individual’s capacity. Collegiate athletes may perform maximum repetition testing with external loads. An elderly individual might use bodyweight assessments or functional tests, such as sitto-stand repetitions [96].

Goal identification establishes what an individual wants to achieve through exercise. Goals vary significantly across populations. Athletic performance goals may focus on sport-specific capabilities, such as improving sprint speed, increasing muscular power, or enhancing endurance capacity. Health-related goals may emphasize weight management, blood pressure reduction, improved glycemic control, and enhanced cardiovascular fitness. Functional goals might target daily activity capabilities, such as climbing stairs without breathlessness, carrying groceries without fatigue, or maintaining independence in activities of daily living. The agent must understand and accommodate these diverse goal structures to achieve optimal results.

Constraint assessment identifies practical limitations affecting programming. Time availability varies from athletes with multiple daily training sessions to busy professionals managing 30-minute windows three times a week. Equipment access ranges from professional training facilities to home settings with minimal equipment availability. Geographic location, financial resources, or mobility constraints may limit facility access. Exercise preferences are significant for adherence. Some individuals enjoy group classes, whereas others prefer solitary exercise. Some people like outdoor activities, while others favor climate-controlled indoor environments. The agent considers these preferences when selecting exercise modalities to maximize adherence.

#### 3.7.3 Evidence-Based Programming and Progression

Once the assessment is complete, the agent generates individualized exercise programs following the established prescription principles from the exercise science literature. The FITT (Frequency, Intensity, Time, Type) principle provides a foundational framework [73, 97]. Frequency specifies how many days per week each exercise modality is performed. Intensity determines how hard each exercise bout should feel, expressed through methods appropriate to each modality, such as target HR zones for aerobic exercise, percentage of onerepetition maximum (1RM) for resistance training, or RPE scales. Time indicates the duration of each session. Type identifies the exercise modalities employed in the program.

For aerobic exercise prescription, guidelines recommend 150 to 300 minutes of moderate-intensity or 75 to 150 minutes of vigorousintensity aerobic activity weekly for general health benefits [98, 99]. The agent adapts these guidelines based on individual goals and constraints. Individuals seeking maximum cardiovascular adaptation may receive programming at the upper end or beyond these ranges. Individuals beginning exercise after prolonged sedentary behavior would start well below these targets with gradual progression.

Intensity prescription requires particular care, given the diverse target populations. For young healthy individuals, intensity might be prescribed as HR zones calculated from the age-predicted maximum HR [100]. For individuals with cardiovascular disease or those taking HR-affecting medications, alternative intensity markers such as the RPE or talk test guidelines should be used [101]. For clinical populations, the agent incorporates specific precautions. Individuals with uncontrolled arterial hypertension should not perform isometric exercises or heavy resistance training [102]. Individuals with proliferative diabetic retinopathy should avoid high-intensity aerobic exercise or resistance training with the Valsalva maneuver [103].

Resistance training prescriptions would follow similar evidencebased frameworks. For muscular strength development, the agent typically prescribes higher loads (70% to 85% of 1RM) with lower repetitions (6 to 12) and longer rest periods (2 to 3 min) [104]. For muscular endurance, the agent prescribes lower loads (less than 70% of 1RM) with higher repetitions (12 to 20) and shorter rest periods (less than 1-min). For power development in athletes, the agent would prescribe moderate loads (30% to 60% of 1RM) performed explosively with adequate rest for full recovery between sets [105].

The agent provided detailed instructions for each prescribed exercise. Text descriptions explain the proper technique, emphasizing key points for safety and effectiveness. Video demonstrations would show correct movement patterns from multiple angles. Common errors received specific attention with explanations of what to avoid and why. Equipment setup instructions would ensure proper configuration. Scaling options would accommodate different fitness levels, allowing individuals to adjust the difficulty appropriately.

Progression algorithms would determine when and how to advance the programming difficulty. For beginners, the agent would emphasize gradual increases, prioritizing technique mastery before advancing intensity. The initial weeks would focus on learning proper movement patterns with light loads or modified exercises. Subsequent progression increases the volume (sets and repetitions) before increasing the intensity (load or speed). This sequence allows physiological adaptation to occur before imposing a greater mechanical stress.

The agent monitors several indicators to determine progression readiness. Technical proficiency would be assessed through self-report questionnaires asking individuals to evaluate their comfort and confidence with current exercises. When someone reports that the exercises feel easy and the technique feels solid, progression consideration begins. Physiological adaptation markers include HR responses to standardized work rates decreasing over time; declining RPE scores for given intensities, and shortening recovery time between sessions. Performance improvements can be directly assessed through periodic testing or inferred from training data showing increasing work capacity.

When progression occurs, the agent implements conservative increases to minimize the risk of injury. The 10% rule provides a general guideline: weekly increases in training volume should not exceed 10% of the previous week’s total [106]. The agent applies this principle flexibly based on individual circumstances. Individuals with an extensive training history might tolerate larger increases. Individuals with a history of injury or medical conditions would receive progressions that are more conservative.

#### 3.7.4 Non-Communicable Disease Applications

A critical benefit of exercise prescription agents is the expansion of access to evidence-based programming for clinical populations managing non-communicable diseases. Cardiovascular disease, T2DM, chronic obstructive pulmonary disease, osteoarthritis, osteoporosis, and numerous other conditions benefit from appropriate exercise interventions [107, 108]. However, many patients lack access to clinical exercise physiologists or physical therapists who can provide specialized programs.

Exercise prescription agents could partially address this gap; however, important caveats apply. The agent provided programming only after medical clearance. Individuals diagnosed with chronic diseases must obtain physician approval before beginning exercise programs. The agent cannot be substituted for medical evaluation. Once cleared for exercise, the agent can provide individualized programming following established clinical guidelines.

For individuals with T2DM, exercise prescription should follow specific principles that address metabolic goals. The agent emphasizes moderate-intensity aerobic exercise accumulated through multiple daily sessions to maximize the effects of glucose uptake [109]. This includes resistance training to preserve and build muscle mass, which improves insulin sensitivity [110]. It would provide timing guidance, recommending exercise during periods of adequate glycemic control rather than when glycaemia levels are extremely high or low. This includes safety precautions regarding the risk of hypoglycemia, particularly in individuals taking insulin or sulfonylureas. The agent reminds users to monitor glycaemia before and after exercise, carry rapid-acting carbohydrates during exercise, and avoid exercising when glycaemia exceeds 250 mg/dL with ketones present [111]. Exercise prescription for individuals with cardiovascular disease requires particular caution [112]. The agent implements strict intensity limits based on medical clearance information. Individuals who have undergone cardiac rehabilitation would receive exercise prescriptions based on their rehabilitation program. Those with recent cardiac events or procedures would require updated medical clearance before progression [112]. The agent would monitor for warning symptoms, including chest pain or discomfort, unusual shortness of breath, dizziness or light-headedness, palpitations or irregular HR, or excessive fatigue during or after exercise. Any concerning symptoms triggered automatic alerts recommending immediate medical consultation.

For individuals with chronic obstructive pulmonary disease, the agent would prescribe exercise programs emphasizing aerobic training at moderate intensity with frequent rest intervals [113]. This includes upper body strength training to improve respiratory muscle function. It would provide breathing technique instructions emphasizing pursed-lip breathing during exertion. It would monitor oxygen saturation levels where measurement equipment is available, recommending medical consultation if saturation falls below 88% during exercise [114].

#### 3.7.5 Autonomous Operation and Human Oversight Balance

The theoretical exercise prescription agent would operate with substantial autonomy in routine programming decisions. Once the initial assessment is completed and medical clearance is confirmed, the agent generates and implements exercise prescriptions without requiring human approval for each decision. The agent would make automatic adjustments based on performance and feedback, including increasing difficulty when users report that exercises feel easy, decreasing difficulty when users report that exercises feel too challenging or cause discomfort, modifying exercise selections when users report disliking specific exercises and adapting schedules when users cannot complete planned sessions.

However, several situations could trigger an escalation in human oversight. Medical symptoms would require immediate medical evaluation rather than agent-managed responses. New or worsening pain patterns, particularly in the chest, left arm, jaw, or upper back, require medical assessment [115]. Significant injuries during exercise would require evaluation by healthcare providers before exercise is resumed [116]. Persistent difficulty tolerating prescribed exercise

despite multiple modifications would suggest the need for professional evaluation to ensure that no underlying medical issues prevent exercise participation. The agent clearly communicates its limitations to the users. It does not diagnose medical conditions, cannot clear individuals for exercise without physician approval, cannot treat injuries or medical problems, and cannot replace professional medical care. Users will receive explicit information about these limitations during the initial setup and at regular intervals throughout the ongoing use.

For individuals with access to exercise professionals, the agent would function as a complementary tool rather than a replacement. Sports scientists or personal trainers can configure the agent’s parameters, review its programming decisions, override recommendations when clinical judgment suggests alternative approaches, and maintain oversight while delegating routine monitoring and adjustment tasks to the agent. This supervised approach represents an optimal implementation model.

For individuals without access to exercise professionals, the agent would provide a valuable resource, while acknowledging the inherent limitations of unsupervised use. The agent implements conservative approaches, prioritizing safety over optimal progression. It would err toward underdosing rather than overdosing when there is uncertainty. It would provide extensive education on warning signs requiring medical attention. This would encourage periodic reassessment by healthcare providers, ensuring that exercise programming remains appropriate as health status changes.

This dual-access model addresses health equity concerns while maintaining safety. Individuals in well-resourced environments receive optimal human-supervised, agent-augmented care. Individuals in underserved settings gain access to evidence-based programming that exceeds unstructured guidance. The agent does not eliminate disparities but reduces them relative to the current realities in which underserved populations often receive no structured exercise guidance.

**FIG. 7:**
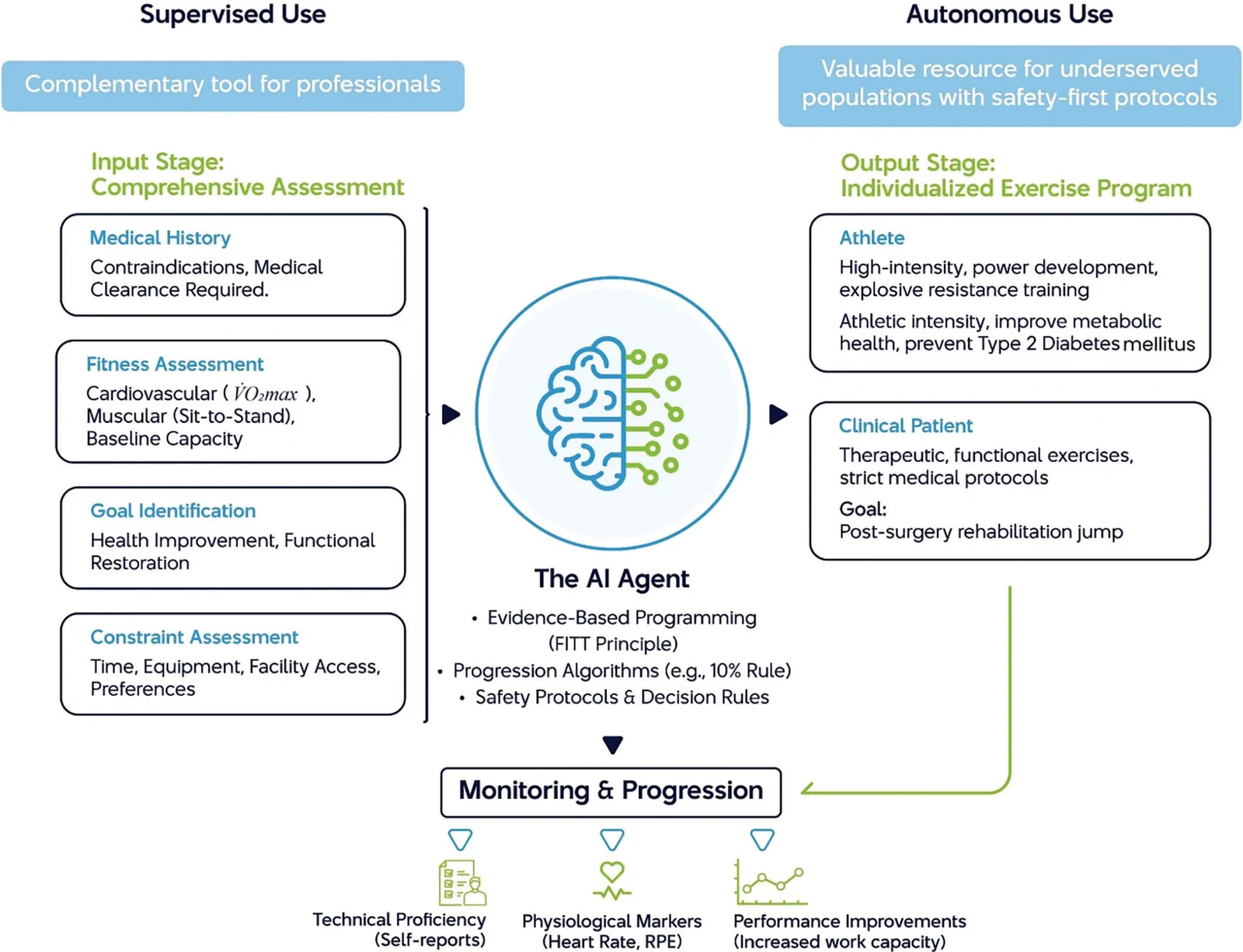
Artificial Intelligence (AI) Exercise Prescription Agent: Comprehensive Assessment to Individualized Programming and Adaptive Monitoring. FITT: Frequency, Intensity, Time, Type; RPE: Rating of Perceived Exertion; ${\dot{\text{V}}\text{O}}_{2\max}$: Maximal Oxygen Uptake.

Figure 7 illustrates the AI-powered system’s comprehensive approach, from multi-domain user assessment and intelligent processing to the generation of individualized exercise programs for diverse populations, all supported by continuous monitoring and flexible oversight models.

### 3.8 Theoretical Application Domain 3: Biomechanical Analysis and Technique Optimization Agent

Biomechanical analysis represents a critical domain in which autonomous agents could theoretically transform both athletic skill development and clinical movement rehabilitation. Current approaches rely heavily on periodic video analysis sessions in which coaches or clinicians review footage after training completion, identify technical flaws, and provide feedback during subsequent sessions [9]. This delayed feedback cycle may be suboptimal for certain motor learning objectives, depending on task characteristics and learner stage [117, 118]. Research has demonstrated that immediate feedback during skill execution produces superior learning outcomes compared to delayed correction [117, 118]. However, human practitioners cannot provide continuous real-time analysis during every training repetition, given the cognitive demands of a detailed biomechanical assessment.

#### 3.8.1 Proposed Agent Architecture and Functionality

We propose a theoretical biomechanical analysis agent that employs computer vision algorithms to track human movement from video footage captured during training sessions. Modern pose estimation systems, such as *OpenPose* and *DeepLabCut*, can extract joint positions from standard video cameras with sufficient accuracy for many athletic applications [119, 120]. The agent would process video streams in real time, extracting three-dimensional joint position data throughout movement execution.

For overhead throwing sports, such as baseball pitching, the agent would monitor specific biomechanical parameters established in the literature as performance and injury risk factors. The shoulder external rotation angle at the maximum cocking position in healthy adult professional pitchers, typically ranges from 165° to 180° as measured by three-dimensional motion analysis [121]. Excessive rotation beyond this range correlates with an increased injury risk to the ulnar collateral ligament [122]. Insufficient hip and trunk rotation forces compensate for the throwing arm, increasing the elbow valgus stress. Kinetic chain timing deficits, where lower body rotation fails to precede upper body rotation, reduce throwing velocity while increasing joint loading [123].

The agent detects deviations from the optimal patterns during each throwing repetition. When shoulder external rotation exceeds safe thresholds, the agent could, in principle, deliver immediate audio cues, for example: “Reduce shoulder layback, keep elbow below 175 degrees”. When hip rotation appears insufficient relative to trunk rotation, the feedback would become: “Drive harder with your back hip before rotating your trunk”. This real-time correction

The agent would maintain individualized biomechanical profiles for each athlete rather than applying rigid, standardized models. Some throwers naturally exhibit greater shoulder external rotation without an increased injury risk. The agent learns each athlete’s normal range through extended monitoring. Deviations from an individual’s typical patterns would trigger attention, even when absolute values fall within population norms. An athlete who typically demonstrates 170° of shoulder external rotation but suddenly shows 180° would receive feedback about this change despite both values being within the general guidelines. Individual baselines would be defined as rolling means calculated across a minimum monitoring window (proposed: 14–21 sessions), with deviations flagged when an athlete’s value exceeds a pre-specified percentage departure (e.g., ± 10%) from their own running mean.

#### 3.8.2 Sport-Specific Technical Monitoring

Different sports require specialized biomechanical monitoring that is adapted to their unique technical demands [9]. In swimming, technique analysis focuses on body position, stroke mechanics, and breathing patterns [124]. The agent would track the head position relative to the waterline (e.g., excessive head lift increases drag), hip position relative to the surface (e.g., dropped hips increase resistance), elbow bend during the catch phase (e.g., straight arm catch reduces propulsion [125]), and rotation timing between body roll and arm pull. Swimmers would receive real-time audio feedback through bone conduction headphones: “Keep your head lower in the water” or “Bend your elbow earlier in the catch”.

For Olympic weightlifting, the agent would monitor the bar path trajectory, joint angles during the catch position, timing of hip extension relative to knee extension, and landing positions after the pull. The proper bar path remains close to the body throughout the lift [126]. Excessive horizontal displacement indicates a technical inefficiency. The agent would detect these deviations and provide corrective feedback: “Keep the bar closer to your body during the second pull” or “Drive your hips forward before jumping”.

In an injury prevention context, running gait analysis focuses on parameters associated with overuse injury risk; in a performance context, it targets biomechanical efficiency and running economy [127, 128]. Overstriding (e.g., foot contact well anterior to the center of mass), excessive vertical oscillation (e.g., bouncing), asymmetrical ground contact times, and crossover gait patterns should all be monitored [127, 128]. The agent would provide real-time feedback during treadmill running or outdoor sessions using smartphone video analysis: “Increase your cadence by 5%, shorten your stride length” or “Land with your foot closer to your body”.

For golf swing analysis, the agent tracks the club path, face angle at impact, swing plane consistency, weight transfer patterns, and kinematic sequence. The kinematic sequence describes the order and timing of segment rotations from the ground up through the pelvis, trunk, arms, and club [129]. Optimal sequences generate maximum clubhead speed while maintaining the control. The agent would detect sequence deviations: “Start your downswing with your hips, not your arms” or “Maintain your spine angle through impact”.

For tennis stroke production, the agent analyzes racquet preparation timing, body rotation patterns, contact point positions, and follow-through mechanics. Research has established optimal contact point positions for different stroke types [130]. The agent would monitor whether athletes achieve these positions consistently and provide corrections when deviations occur: “Contact the ball further in front on your forehand” or “Rotate your shoulders more before the racquet moves”.

#### 3.8.3 Clinical Applications for Movement Rehabilitation

Beyond athletic performance optimization, biomechanical analysis offers substantial theoretical potential in clinical rehabilitation settings [9]. Gait analysis following lower extremity surgery or injury can identify compensatory movement patterns that increase fall risk or contribute to secondary problems [131]. An elderly patient recovering from total knee replacement may show reduced knee flexion during the stance phase, compensating for excessive hip hiking on the surgical side. This asymmetrical pattern increases the metabolic cost of walking, delays functional recovery, and potentially contributes to low back pain from repetitive asymmetrical loading [75].

The rehabilitation-focused agent would track gait parameters through video analysis as the patient walked on a treadmill or through the clinic. When compensatory patterns appear, the agent would provide verbal cueing: “Bend your surgical knee more during stance” or “Level your hips, don’t hike your right side”. The physical therapist would supervise the session but delegate continuous monitoring to the agent, freeing their attention for other rehabilitation activities or working with multiple patients.

The agent also automatically adjusts the exercise prescription based on the observed movement quality. If a patient demonstrates poor form during a prescribed exercise despite multiple cueing attempts, the agent could identify that the exercise exceeds the patient’s current motor control capacity. It would automatically regress the exercise to an easier variation, allowing for proper technique. For example, a single-leg squat might regress to a supported single-leg squat holding a TRX strap, then to a bilateral squat, until the patient develops sufficient strength and balance for an unsupported singleleg execution.

Gait training is a critical intervention for individuals with Parkinson’s disease [76]. The agent monitors gait parameters, including step length, cadence, arm swing amplitude, and postural stability [132]. Parkinson’s disease typically produces a shortened step length, reduced cadence, diminished arm swing, and forward-flexed posture [133]. The agent would provide rhythmic auditory cueing to improve gait parameters: “Step longer, match the beat” or “Swing your arms more”. Research has demonstrated that rhythmic auditory cueing improves gait parameters in patients with Parkinson’s disease [82].

#### 3.8.4 Integration with Training Load Management

In this theoretical coordination scenario, the theoretical biomechanical analysis agent would coordinate with the TLMA to provide comprehensive athlete-monitoring. When the training load increases substantially, movement quality often deteriorates owing to accumulated fatigue. The biomechanical agent would detect these quality decrements and would communicate them to the TLMA. This information would influence load management decisions. An athlete showing progressive deterioration in throwing mechanics across a practice session might trigger a training load reduction, even if absolute workload metrics remain within acceptable ranges..

A practical coordination scenario is considered. A baseball pitcher completes bullpen sessions monitored by both a TLMA (e.g., tracking pitch counts, velocities, and arm slot consistency) and a biomechanical agent (e.g., analyzing shoulder external rotation, elbow flexion angles, and kinetic chain timing). Midway through the session, the biomechanical agent detects that shoulder external rotation has increased from the athlete’s normal 172° to 178° over the past 15 pitches. Simultaneously, elbow flexion at foot contact decreased, indicating a fatigue-related technique breakdown. The biomechanical agent sends a message to the TLMA: “Athlete X showing biomechanical fatigue indicators. Shoulder external rotation increased by 3.5%, and elbow flexion decreased by 8% from the baseline. Recommend session termination”. The TLMA reviews recent workload data, notes that today represents the third consecutive day of throwing after a high-volume week, and determines that the biomechanical concerns combined with workload accumulation warrant immediate termination of the session. It would automatically stop the session, notifies the pitching coach with a detailed rationale, and schedules two recovery days before the next throwing session.

Conversely, when the TLMA implements recovery periods, the biomechanical agent adjusts its expectations. Movement quality typically improves following recovery, and the agent would use this improvement as validation that the training load adjustment achieved the intended effects. If movement quality fails to improve during recovery periods, this suggests either that recovery is insufficient or that biomechanical problems stem from structural limitations rather than fatigue.

#### 3.8.5 Proposed Validation Approach

Validating a theoretical biomechanical analysis agent requires demonstrating both technical accuracy and performance or injury outcome. Technical validation would assess whether the agent accurately identified biomechanical deviations compared to expert human analysis. Researchers would record athletes performing their sports skills while both the agent and experienced bio-mechanists evaluate the technique. The agreement between the agent and human assessments would quantify technical accuracy. Disagreements underwent detailed analysis to understand whether the agent missed subtle deviations or falsely flagged normal variations.

Performance validation will examine whether athletes using agentguided feedback improve technical proficiency and performance outcomes faster than those receiving traditional periodic video analysis. A RCT would compare skill development rates, performance measures (e.g., throwing velocity, swimming times, lifting loads), and subjective assessments of technique quality between agent-supported and traditionally coached groups. For example, novice swimmers might be randomized to receive either real-time agent feedback during every practice session or traditional coach feedback provided once a week during video review sessions. Primary outcomes would include swim time improvements over 12 weeks, stroke efficiency measures, and technique rating scores by blinded expert evaluators.

Injury prevention validation represents the most critical outcome, given the proposed application of biomechanical agents for injury risk reduction. Prospective cohort studies should track injury rates in athletes using versus not using agent-based biomechanical monitoring. The hypothesis predicts that real-time correction of highrisk movement patterns will reduce the incidence of injury. For baseball pitchers, a multi-season cohort study might compare elbow and shoulder injury rates between pitchers using biomechanical agents providing real-time feedback about excessive shoulder external rotation versus pitchers receiving traditional coaching without real-time biomechanical monitoring. However, this hypothesis requires empirical testing rather than assumptions. It remains theoretically possible that constant feedback could paradoxically impair performance by inducing conscious control of normally automated movements, a phenomenon known as “paralysis by analysis” in the motor learning literature [83].

**FIG. 8:**
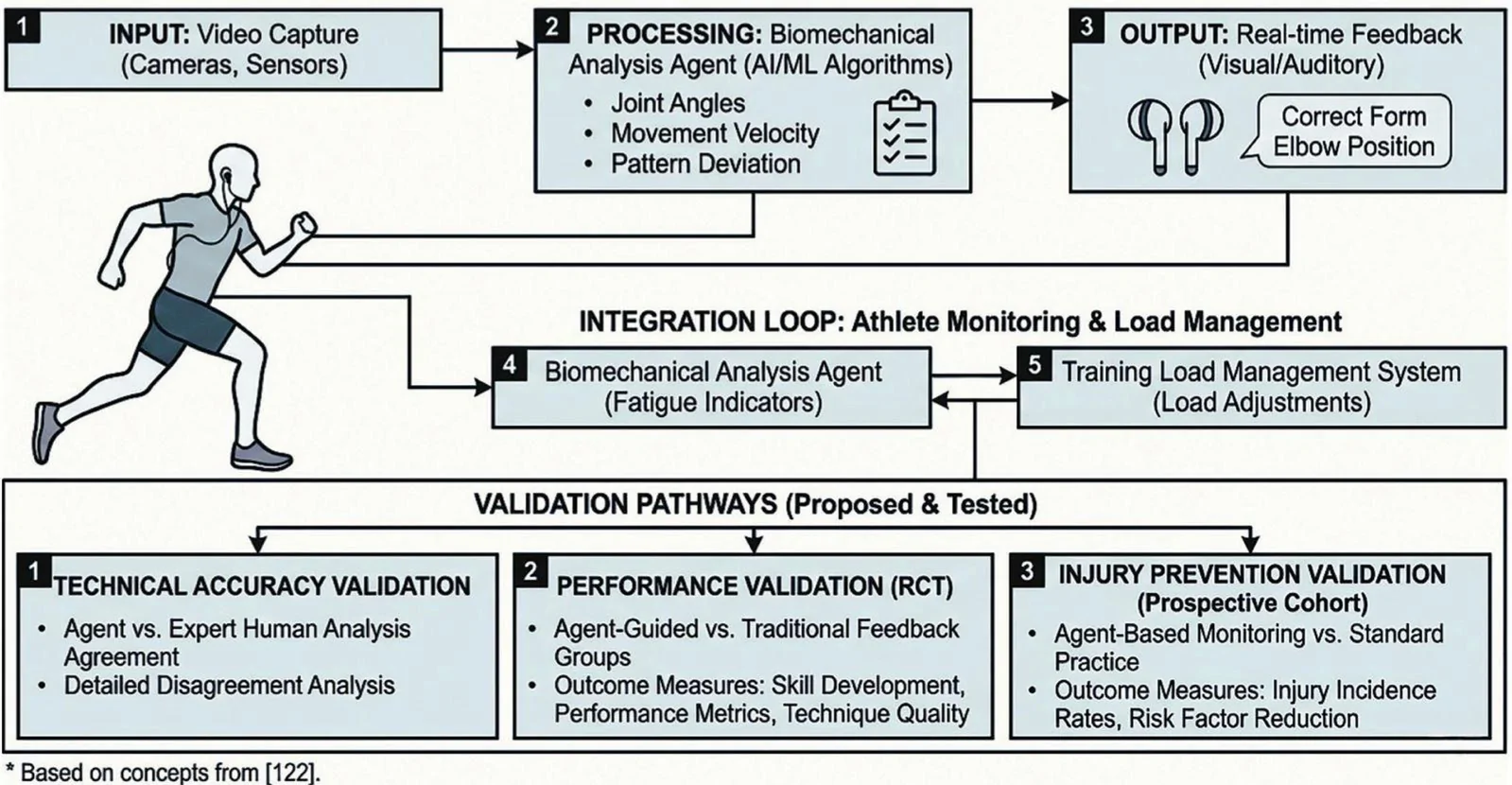
Real-Time Biomechanical Analysis for Integrated Athlete Monitoring. AI: Artificial Intelligence; ML: Machine Learning; RCT: Randomized Controlled Study.

Figure 8 illustrates a system for real-time biomechanical analysis, from video capture and automated detection of movement deviations to immediate feedback. It also depicts the integration of this analysis with training load management for comprehensive athlete monitoring.

### 3.9 Theoretical Application Domain 4: Nutrition Optimization Agent

Nutrition profoundly affects athletic performance, recovery, body composition, and health outcomes [134, 135]. However, many athletes receive suboptimal nutritional guidance owing to limited access to sports nutritionists qualified to provide individualized programs [136]. The nutrition domain presents particularly complex challenges for autonomous agents, given the enormous individual variability in energy requirements, macronutrient preferences, cultural food traditions, budget constraints, and physiological responses to dietary interventions [137]. All the content in this section represents theoretical proposals that require empirical validation.

#### 3.9.1 Comprehensive Nutrition Management Scope

Preliminary evidence that ML frameworks can automate nutritional screening stably across maturation phases in athletic youth populations [138] supports the technical feasibility of fully autonomous nutrition agents. We propose a theoretical nutrition optimization agent, beginning with a comprehensive multidimensional assessment. Body composition analysis using bioelectrical impedance, skinfold measurements, or dual-energy X-ray absorptiometry can establish baseline fat mass, lean mass, and bone density [139]. The agent would use these data to calculate the resting metabolic rate using the Mifflin-St Jeor equation [140], which accounts for age, sex, height, weight, and body mass as the currently recommended approach for resting metabolic rate prediction.

Energy expenditure estimation integrates the resting metabolic rate with activity energy expenditure derived from training load data. The TLMA shared information about the daily training volume and intensity. The nutrition agent would apply sport-specific energy cost equations to convert training data into estimated kilocalories expended. For endurance training, the equations would account for distance, speed, terrain grade, and body weight [141]. For resistance training, equations would consider volume-load (i.e., sets × repetitions × load), rest intervals, and session duration [142].

The agent establishes specific goals through structured dialogue with the athlete. Performance-focused goals might emphasize muscle hypertrophy, requiring a caloric surplus and elevated protein intake; fat loss while preserving lean mass, requiring a caloric deficit with high protein; or weight maintenance with optimized body composition. Health-focused goals may target improved metabolic markers, reduced cardiovascular risk, or management of food allergies and intolerances. The agent documents these goals with specific quantitative targets: increase lean mass by 3 kg over 12 weeks, reduce body fat percentage from 18% to 15% over 8 weeks, or maintain current weight within 1 kg throughout the competition season.

Dietary preferences and restrictions will be thoroughly documented. The agent would inquire about vegetarian or vegan dietary patterns, religious restrictions affecting food choices (e.g., halal, kosher, no beef, no pork), cultural food traditions the athlete wanted to maintain, specific food allergies requiring strict avoidance (e.g., peanuts, shellfish, gluten), food intolerances affecting digestion (e.g., lactose intolerance, FodMAP (Fermentable, Oligosaccharides, Disaccharides, Monosaccharides, And Polyols) sensitivity), and personal taste preferences. This information constrains food selection for meal planning while ensuring that the recommendations are practically implementable.

#### 3.9.2 Macronutrient Targeting and Meal Planning

The agent calculates individualized macronutrient targets based on established sports nutrition principles. Protein requirements typically range from 1.6 to 2.2 g/kg body weight daily for athletes, with higher values during caloric restriction or intensive training [143]. Carbohydrate requirements vary substantially based on training volume and intensity, ranging from 3–12 g/kg body weight/day [144]. Low-intensity training or rest days may require only 3–5 g/kg, whereas high-volume endurance training may necessitate 8–12 g/kg. Fat intake generally comprises 20% to 35% of the total energy intake, with an emphasis on unsaturated fats [145].

The agent generates daily meal plans that meet these targets while accommodating individual constraints. It would select recipes from extensive databases that match user preferences. It would adjust portion sizes to achieve macronutrient goals precisely. Meals should be timed appropriately around training sessions to optimize nutrient availability and recovery. Pre-training meals would emphasize easily digestible carbohydrates consumed 2–3 hours before exercise for energy availability [146]. Post-training meals should combine carbohydrates for glycogen restoration with protein for muscle protein synthesis and should ideally be consumed within 2 hours of training completion [147].

For a 75 kg endurance runner requiring 8 g/kg carbohydrate (600 g), 1.8 g/kg protein (135 g), and approximately 3,500 total daily calories, the agent would generate a structured meal plan distributing energy across five to six eating occasions, prioritizing easily digestible carbohydrate in the two to three hours before training and combined carbohydrate-protein intake within two hours after training to support glycogen restoration and muscle protein synthesis [147, 148].

The agent adapts recommendations dynamically based on changing circumstances. When the TLMA communicates upcoming intensive training, the nutrition agent automatically increases carbohydrate allocation. When body composition monitoring reveals fat loss exceeding targets, the agent increases energy intake to prevent excessive loss of lean mass. When an athlete reports experiencing gastrointestinal distress with certain pre-training meals, the agent would modify food selections to improve tolerance, perhaps by reducing the fiber or fat content of meals consumed close to training.

#### 3.9.3 Supplement Recommendations and Anti-Doping Considerations

Supplement recommendations should follow evidence-based guidelines [148]. The agent would suggest only supplements with strong research support for an individual’s specific goals. For muscle hypertrophy, creatine monohydrate may be considered, given the extensive evidence for its efficacy and safety when dosed at 3–5 grams daily [149]. For endurance performance, caffeine (3–6 mg/kg consumed 60 min before exercise) and sodium bicarbonate (0.3 g/kg consumed 60–90 minutes before exercise) may be considered based on event characteristics [150, 151]. The agent avoided recommending supplements with weak evidence, potential health risks, or antidoping concerns.

Anti-doping considerations are critical for competitive athletes [152]. The agent cross-referenced all supplement recommendations against the World Anti-Doping Agency prohibited substance list [153]. This would warn athletes that supplements carry contamination risks, even when the primary ingredient is permitted. Research indicates that a substantial percentage of nutritional supplements contain undeclared prohibited substances [154]. The agent recommends that athletes use only supplements certified through third-party testing programs that screen for prohibited substances. When athletes inquire about supplements lacking strong evidence or carrying potential risks, the agent provides balanced information. For beta-alanine supplementation, claimed to improve high-intensity exercise performance, the agent would explain that evidence shows modest benefits for exercise lasting 60–240 seconds, typical dosing is 3–6 grams daily split into multiple doses, and common side effects include paresthesia (tingling sensations) that are harmless but uncomfortable [155]. The agent would not recommend or discourage use, but would provide information allowing for informed decisions.

#### 3.9.4 Hydration Strategy Optimization

Hydration is another critical nutritional domain that requires individualized guidance [156]. Weight loss during training, under conditions of no fluid intake during the measurement session, primarily reflects fluid loss through sweating [157]. The agent would use these data to calculate sweat rates: a runner losing 1.5 kg during a 60-minute session in 25°C ambient temperature has a sweat rate of approximately 1.5 L/h under those conditions [158].

The agent adjusted the hydration recommendations based on the environmental conditions. Heat, humidity, and altitude affect the sweat rate and fluid requirements [157]. Training in 35°C heat might increase sweat rates by 50–100% compared to 20°C conditions [159]. The agent accounts for these factors and recommends increased fluid intake before, during, and after training when environmental stress is elevated.

For prolonged training sessions exceeding 60–90 minutes, the agent would recommend consuming fluids containing carbohydrates and electrolytes rather than plain water alone. Carbohydrate consumption during exercise maintains blood glucose levels and delays fatigue [160]. Sodium replacement prevents hyponatremia, which can occur when athletes consume large volumes of plain water during prolonged exercise [161]. The agent would recommend sports drinks providing 6–8% carbohydrate solution (60–80 g carbohydrate per liter) and 20–30 mmol/L sodium [162].

#### 3.9.5 Coordination with Training Load and Recovery Agents

The theoretical nutrition agent coordinates extensively with other domain agents. When the TLMA identifies an upcoming period of intensive training, it would send notification to the nutrition agent: “Athlete X entering high-volume training block, daily energy expenditure estimated to increase by 800 kcal for 10 days starting tomorrow”. The nutrition agent would respond by automatically adjusting meal plans, increasing carbohydrate allocation from 6 to 8 g/kg, distributing additional energy across multiple meals and snacks, and emphasizing nutrient timing around the training sessions.

When the sleep and recovery agent detects poor sleep quality, it communicates with the nutrition agent regarding potential dietary interventions to support sleep. Nutrition agents might recommend an earlier dinner time (e.g., finishing meals 3–4 hours before bedtime to allow digestion), increased carbohydrate intake at the evening meal (e.g., carbohydrate consumption in the evening may improve sleep quality [163]), avoidance of caffeine after 2 PM, and consideration of tart cherry juice consumption in the evening (e.g., contains melatonin and may improve sleep duration [164]).

When the injury prevention agent identifies an elevated injury risk requiring training load reduction, it notifies the nutrition agent. The nutrition agent would adjust energy intake recommendations downward to match the reduced energy expenditure, preventing unwanted weight gain during the recovery period. However, it would maintain elevated protein intake (2.0–2.2 g/kg rather than the typical 1.6–1.8 g/kg) because higher protein intake during periods of reduced training helps preserve lean mass [165].

#### 3.9.6 Special Populations’ Considerations

The nutrition agent would adapt their approach for special populations with unique nutritional needs. Female athletes face specific considerations, including adequate energy availability to prevent relative energy deficiency in sports, iron intake given menstrual blood losses, and calcium and vitamin D intake for bone health [166]. The agent would monitor energy intake relative to exercise energy expenditure, flagging situations where energy availability falls below 30 kcal per kg fat-free mass per day, the threshold below which hormonal and metabolic disturbances occur [167]. Adolescent athletes require special attention to support their growth and development while meeting training demands. The agent would ensure adequate total energy intake to support both training and growth, calcium and vitamin D intake for bone development, and iron intake for expanding blood volume during adolescent growth spurts [168]. It would avoid recommending caloric restriction for body composition goals in adolescents unless medically supervised, given the risks of impaired growth and development. Master athletes (e.g., typically defined as over age 35–40) experience age-related changes that affect their nutritional needs [169]. Protein requirements may be elevated compared to younger athletes to overcome anabolic resistance and the reduced muscle protein synthesis response to protein intake with aging [170]. The agent recommends protein intake at the upper end of the range (2.0–2.2 g/kg) for master athletes. It emphasizes distributing protein relatively evenly across meals rather than concentrating it in one meal, as even distribution optimizes muscle protein synthesis in older individuals [171].

#### 3.9.7 Proposed Validation Approach

Validating a theoretical nutrition optimization agent requires demonstrating that athlete-adjusted nutritional intake improves relevant outcomes compared to self-directed nutrition or standard dietary advice. RCTs should compare athletes receiving agent-generated individualized meal plans with those receiving standard nutrition education materials. Primary outcomes would include adherence to nutritional targets (e.g., measured through food logging or objective biomarkers), body composition changes (i.e., fat mass and lean mass measured through validated methods), performance outcomes (i.e., sport-specific tests), and subjective measures of recovery quality and training tolerance.

For clinical populations managing non-communicable diseases, validation should focus on disease-specific outcomes. A trial in individuals with T2DM would assess glycemic control (glycated hemoglobin A1c, fasting glycaemia, and glycaemia variability), body weight changes, medication requirements, and cardiovascular risk markers. A trial in individuals with cardiovascular disease would assess blood pressure, lipid profiles, body composition, exercise tolerance, and cardiovascular events.

User experience and adherence are critical outcomes, given that even optimal nutritional plans provide no benefit if athletes do not follow them. Qualitative research would explore factors affecting adherence, including meal plan practicality, food palatability, preparation time requirements, cost, and social impacts. This information can guide agent refinements to improve real-world usability.

**FIG. 9:**
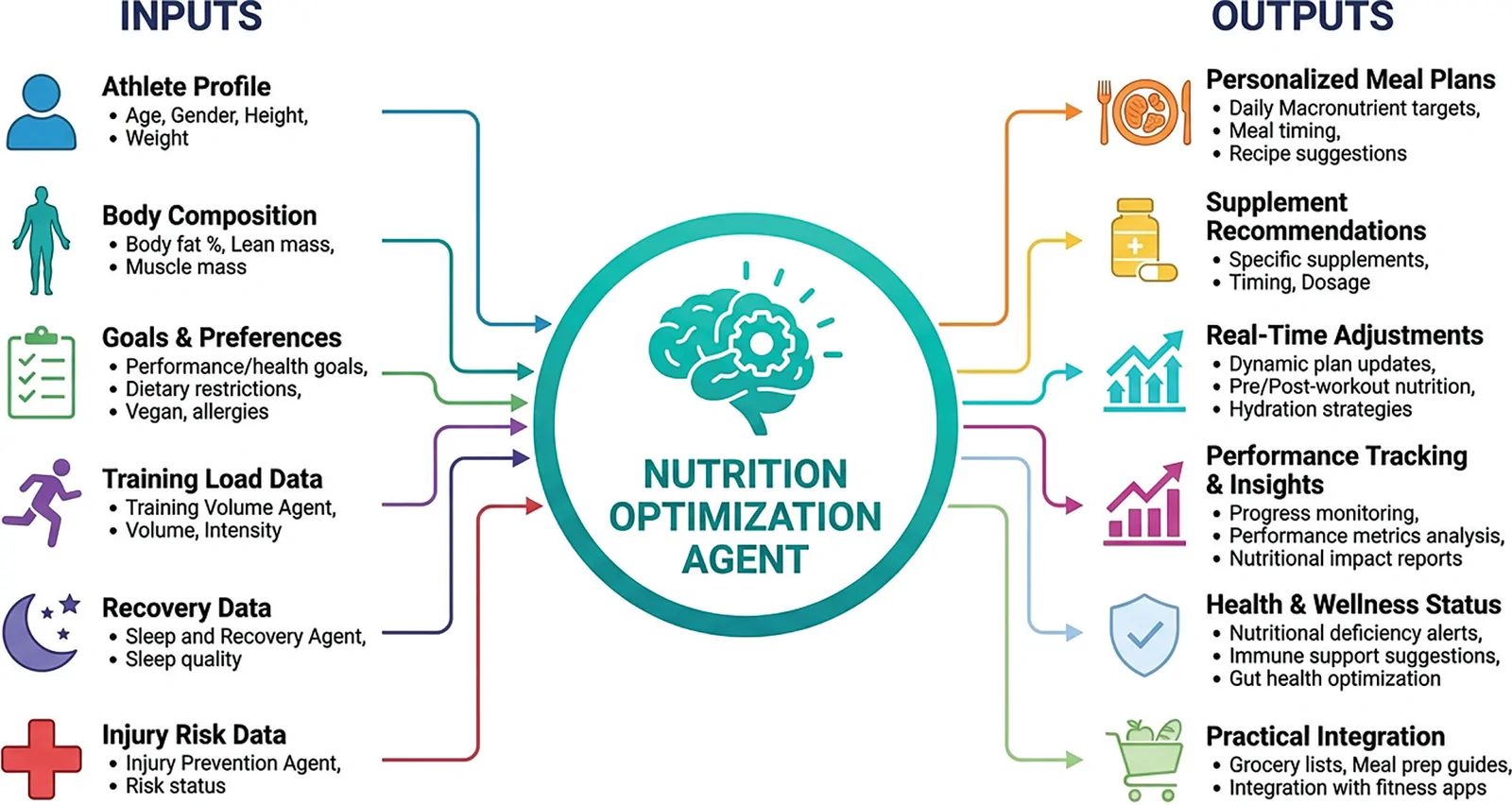
Integrated Architecture of the Nutrition Optimization Agent.

Figure 9 illustrates how the Nutrition Optimization Agent integrates multidimensional data to deliver adaptive, personalized, and scientifically grounded nutrition strategies that evolve through continuous feedback and monitoring.

### 3.10 Theoretical Application Domain 5: Sleep and Recovery Monitoring Agent

Sleep quality profoundly affects athletic performance, injury risk, and health outcomes [172, 173]. Despite this importance, many athletes receive inadequate sleep due to training schedules, academic or occupational demands, traveling across time zones, or poor sleep habits. A theoretical sleep and recovery-monitoring agent would track sleep duration and quality through wearable devices, detect patterns indicating insufficient recovery, provide evidence-based recommendations for sleep optimization, and coordinate with TLMA to adjust the program when sleep deficits occur. All content represents theoretical proposals requiring empirical validation.

#### 3.10.1 Sleep Monitoring and Analysis

Modern wearable devices track sleep stages using movement sensors (accelerometers) and HR monitoring. The agent would analyze this data nightly, calculating total sleep duration, time in each sleep stage (i.e., light sleep, deep sleep, rapid-eye movement (REM) sleep), sleep efficiency (i.e., percentage of time in bed actually sleeping), and nighttime HR patterns indicating autonomic nervous system recovery status. Research has established that adults require 7–9 hours of sleep nightly for optimal health and performance, with athletes potentially requiring more sleep given their additional recovery demands [174].

The agent tracked trends over time rather than focusing excessively on single nights. Sleep naturally varies from night to night [175, 176]. One poor night’s sleep typically has minimal impact on performance, whereas chronic sleep restriction (i.e., consistently obtaining insufficient sleep over days or weeks) produces cumulative performance decrements [177]. The agent would calculate rolling averages of sleep duration over 7-day periods, flagging athletes whose average sleep duration fell below 7 hours nightly.

In addition to total sleep duration, sleep architecture is important [175, 176]. Deep sleep (i.e., slow-wave sleep) is particularly important for physical recovery and growth hormone release [178]. REM sleep plays a critical role in memory consolidation and emotional regulation [179]. The agent monitored whether the athletes obtained adequate time in each sleep stage. While optimal distributions remain debated, general guidelines suggest that adults should spend approximately 50–60% of their sleep time in light sleep, 10–20% in deep sleep, and 20–25% in REM sleep consumer wearable devices demonstrate only moderate agreement with polysomnography for sleepstage classification, particularly for slow-wave sleep, and agent recommendations based on wearable sleep-stage data should therefore be interpreted with appropriate caution [180, 181].

HRV during sleep provides information on autonomic nervous system balance and recovery status [182]. Higher HRV generally indicates better recovery and greater parasympathetic nervous system activity. Day-to-day HRV is subject to substantial biological variability influenced by measurement timing, posture, and respiratory rate; interpretation therefore requires rolling averages rather than singleday values [86]. The agent tracked nighttime HRV trends. A declining HRV over consecutive nights suggests accumulating stress or inadequate recovery, even when subjective sleep quality reports remain normal.

#### 3.10.2 Sleep Optimization Interventions

When sleep quality declines, the agent investigates potential causes using structured questionnaires. Stress or anxiety may prevent sleep onset or cause frequent awakenings. Environmental factors, such as noise, light, and temperature, may disrupt sleep. Training timing may interfere with sleep, particularly when intensive sessions occur late in the evening [183, 184]. Caffeine intake may extend too late in the day [185]. Screening time before bedtime may suppress melatonin production [186]. Irregular sleep-wake schedules may disrupt circadian rhythms [187].

The agent provides targeted recommendations to address the identified issues. When sleep quality declines, the agent would investigate potential causes through structured questionnaire prompts and deliver targeted evidence-based recommendations: relaxation techniques for stress-related disruption [188], bedroom environment optimization for environmental factors, and sleep hygiene education addressing schedule consistency, caffeine timing, and screen exposure [189]. Breathing exercises, such as 4-7-8 breathing (i.e., inhale for four counts, hold for seven counts, exhale for eight counts), activate the parasympathetic nervous system, promoting relaxation [190]. Guided meditation delivered through smartphone apps helps quieten mental activity and prevents sleep onset.

For environmental issues, the agent recommends optimizing bedroom conditions. Temperature between 15 and 19°C (i.e., 60–67°F) generally promote better sleep than warmer environments [191]. Complete darkness using blackout curtains or sleep masks prevents light from disrupting the circadian rhythms [192]. White noise machines or earplugs can mask disruptive sounds [193]. Comfortable bedding appropriate for the season prevents temperature-related awakening [194].

The agent provided education on sleep hygiene practices. Maintaining consistent sleep-wake schedules, even on weekends, strengthens circadian rhythms, promoting better sleep quality [195]. Avoiding large meals within 2–3 hours of bedtime prevents digestive discomfort that disrupts sleep. Limiting caffeine intake to morning hours prevents caffeine’s stimulant effects from interfering with sleep onset given caffeine’s 5–6 hour half-life [196]. Reducing screen exposure (e.g., phones, tablets, computers, and televisions) in the hour before bed minimizes blue light exposure, which suppresses melatonin production [197], in elite soccer players, pre-bedtime smartphone use impairs attention, reaction time, and explosive strength the following day, with effects amplified across successive nights [198].

#### 3.10.3 Coordination with Training Load Management

The theoretical sleep agent would coordinate extensively with the TLMA. When sleep quality declines, the sleep agent notifies the TLMA, which then adjusts the program to account for impaired recovery capacity. Empirical evidence confirms that sleep restriction significantly impairs reaction time, agility, and cognitive function in elite athletes [199, 200], providing the performance rationale for autonomous TLMA intervention when sleep deficits accumulate. Consider a practical scenario in which an athlete may complete three consecutive nights obtaining only 5–6 hours of sleep instead of their typical 8 hours, likely due to academic examination stress. The sleep agent detects this pattern and sends a notification to the TLMA: “Athlete X showing sleep deficits: average 5.5 hours over past 3 nights, 30% below normal. Deep sleep duration was reduced by 40%. HRV declined 15% from baseline. Recovery capacity likely impaired”. The TLMA receives this notification and determines appropriate responses. The athlete has a high-intensity interval training session scheduled for the following day. Given the impaired recovery capacity from sleep deficits, the TLMA determines that proceeding with planned high-intensity training carries an elevated injury risk and is likely to produce poor quality work due to fatigue. It automatically modifies the next session from high-intensity intervals to moderate-intensity steady-state training, reducing both the absolute load and intensity. It sends notifications to the athlete and coach explaining the modification and its rationale. It schedules reassessment in 48 h to determine whether additional recovery proves necessary or whether normal training can resume.

Conversely, when athletes obtain exceptional sleep quality, the TLMA may leverage this improved recovery capacity. After three consecutive nights of 9-hour sleep with above-average deep sleep percentages and elevated HRV, the athlete demonstrated an excellent recovery status. The TLMA might advance planned progressions slightly, adding one additional interval set or increasing the intensity by 5%, given the athlete’s demonstrated capacity to handle additional stress.

#### 3.10.4 Travel and Jet Lag Management

For athletes traveling across time zones for competitions, sleep agents would provide evidence-based strategies for minimizing jet lag effects. Jet lag results from a misalignment between the internal circadian rhythms and local time at the destination [201]. The severity depends on the number of time zones crossed and the direction of travel. Eastward travel (e.g., traveling from California to New York) typically produces worse jet lag than westward travel (e.g., traveling from New York to California) because phase advancing the circadian clock is more difficult than phase delaying it [202].

The agent recommends strategies for accelerating circadian rhythm adjustment. Light exposure at appropriate times relative to the travel direction was particularly effective [203]. For eastward travel, bright light exposure in the morning at the destination advances circadian rhythms toward the new time zone [204]. For westward travel, bright light exposure in the evening appropriately delays circadian rhythms. The agent would provide specific timing recommendations: “Seek bright outdoor light between 7–9 AM for the first three days after arrival to help your body adjust to the new time zone”.

Melatonin supplementation can assist in circadian rhythm adjustment when appropriately timed [205, 206]. For eastward travel, taking melatonin (0.5–5 mg) in the evening at the destination advances circadian rhythms [207]. The agent would recommend: “Consider taking 3 mg melatonin at 10 PM local time for the first three nights after arrival to help adjust your sleep-wake cycle”. The agent also provided practical advice on adjusting sleep schedules before departure. For eastward travel, gradually shifting sleep-wake times earlier by 1–2 hours in the days preceding travel can reduce jet lag severity upon arrival [208]. For westward travel, gradually shifting sleep-wake times later achieves a similar pre-adaptation.

#### 3.10.5 Napping Strategies

Strategic napping can provide recovery benefits, particularly for athletes who train twice daily or experience sleep deficits [209]. However, napping requires careful implementation to avoid interfering with the nighttime sleep [210, 211]. The agent provides evidencebased napping guidance. Short naps (e.g., 10–20 minutes) provide alertness and performance benefits without producing sleep inertia (grogginess upon awakening) [212, 213]. Longer naps (e.g., 60–90 minutes) allow the completion of full sleep cycles, including deep sleep and REM sleep, providing greater recovery benefits but risking sleep inertia if awakened mid-cycle [213–215].

Nap timing is important. Napping too late in the day interferes with nighttime sleep onset [210]. The agent recommends napping before 3 PM to minimize interference with nighttime sleep [216]. For athletes training twice daily, strategic napping between training sessions enhances afternoon training quality [217]. The agent would schedule naps 2–3 hours after morning training, allowing time for post-training nutrition and avoiding napping immediately after exercise when elevated body temperature may prevent sleep onset.

#### 3.10.6 Proposed Validation Approach

Validating a theoretical sleep monitoring agent requires demonstrating that agent-guided sleep optimization improves sleep outcomes and, critically, that improved sleep translates into performance or health benefits. RCTs should compare athletes receiving agent-guided sleep recommendations with those receiving general sleep education materials or no specific intervention. Primary outcomes would include objective sleep measures (e.g., duration, efficiency, and architecture assessed through polysomnography or validated wearables), subjective sleep quality (e.g., PSQI or similar instruments), daytime alertness and mood, training quality and tolerance, and performance outcomes in sport-specific tests.

The implementation of this strategy would track adherence to agent recommendations. Athletes might receive recommendations but fail to implement them due to competing demands (e.g., academic workload, social activities, and family responsibilities). Intention-to-treat analysis assesses outcomes based on randomization assignment, regardless of adherence. Per-protocol analysis examines outcomes specifically in athletes who successfully implemented recommendations, providing information about intervention efficacy when properly followed.

Qualitative research would explore the barriers to implementing sleep recommendations and the factors facilitating successful sleep optimization. This information can guide agent refinements by addressing common obstacles and providing more practical and implementable suggestions.

**FIG. 10:**
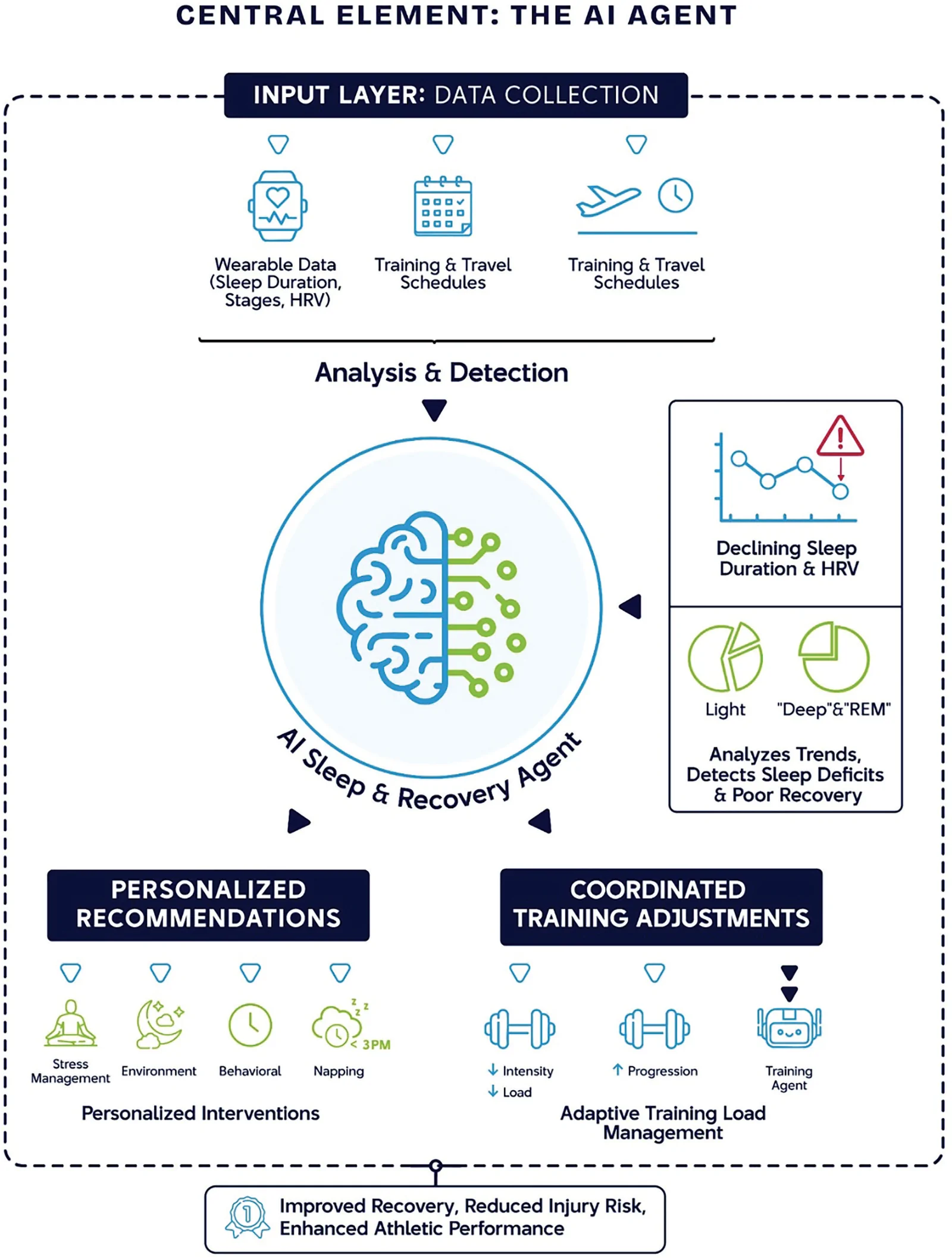
Artificial Intelligence (AI)-Powered Athletic Optimization: A Continuous Loop of Sleep and Recovery Management. HRV: Heart rate variability; REM: Rapid Eye Movement.

Figure 10 represents a graphical abstract that illustrates an intelligent AI agent that continuously monitors an athlete’s sleep, recovery, and training data to deliver personalized interventions and adaptive training adjustments, ultimately enhancing performance and reducing injury risk.

### 3.11 Theoretical Application Domain 6: Injury Prevention Agent

Injury prevention represents one of the most critical applications of AI agents in sports science, given the substantial personal, performance, and economic costs of athletic injuries [218–221]. A theoretical injury prevention agent integrates information from multiple sources to provide comprehensive risk assessment and intervention recommendations. Unlike TLMAs that focus primarily on workload patterns, injury prevention agents would incorporate biomechanical screening data, strength and flexibility assessments, medical history, including previous injuries, psychological factors, and environmental conditions affecting injury risk. All content represents theoretical proposals requiring empirical validation.

#### 3.11.1 Multi-Modal Data Integration for Risk Assessment

The theoretical injury prevention agent would synthesize information across multiple domains to create comprehensive risk profiles. Training load data were obtained from the TLMA, providing acute and chronic workload metrics, training monotony and strain calculations, and recent load changes. Biomechanical data were obtained from a biomechanical analysis agent, providing information on movement asymmetries, technique deviations from optimal patterns, and fatiguerelated quality decrements. Strength and flexibility assessments would be based on periodic testing to identify deficits in muscle strength, power, or range of motion associated with injury risk. The medical history includes previous injuries, surgical procedures, chronic conditions, and family history of certain injuries. Psychological factors would include competitive anxiety, fear of re-injury following previous injuries, and overall stress levels. Environmental factors would include playing surface characteristics, weather conditions, and equipment quality.

The agent would apply sport-specific injury risk models developed from epidemiological studies. For soccer players, anterior cruciate ligament (ACL) injury prevention would receive particular emphasis, given the high incidence in this population [222]. Risk factors for ACL injury include female sex, previous ACL injury, family history of ACL injury, decreased neuromuscular control during landing tasks, knee valgus collapse during cutting maneuvers, and strength imbalances between the quadriceps and hamstrings [223, 224]. The agent assesses which risk factors apply to each athlete and calculates individualized risk estimates. For runners, the agent focuses on common overuse injuries, such as patellofemoral pain syndrome, iliotibial band syndrome, Achilles tendinopathy, and tibial stress injuries. Risk factors include training load errors (i.e., rapid increases in mileage), biomechanical factors (i.e., excessive pronation and reduced hip strength), previous injury history, and anatomical factors [225, 226]. The agent implements screening protocols to assess these risk factors and provides risk estimates for specific injury types.

#### 3.11.2 Evidence-Based Injury Prevention Programming

When an agent identifies an elevated injury risk, evidence-based prevention programs tailored to sport-specific risks and individual vulnerabilities are implemented. For soccer players at elevated ACL injury risk, agents prescribe neuromuscular training programs that have demonstrated efficacy in reducing ACL injury rates. These programs typically include balance exercises performed on unstable surfaces, plyometric training emphasizing proper landing mechanics, hip abductor and external rotator strengthening targeting a strength ratio at or above quadriceps strength [227], and sport-specific cutting and deceleration drills with biomechanical feedback from the biomechanical analysis agent [228, 229].

For a practical example, consider a female soccer player with identified risk factors, including knee valgus during landing tasks and reduced hip abductor strength (i.e., less than 80% of quadriceps strength, below the recommended ratio [227]). The injury prevention agent prescribes a comprehensive program.

Balance training was performed three times a week, including single-leg stance progressions (firm surface → foam pad → BOSU ball), single-leg reaches in multiple directions, and single-leg Romanian deadlifts.

Plyometric training was performed twice a week, emphasizing proper landing mechanics. The exercises included box jumps with video feedback on the landing position, broad jumps with emphasis on soft landings and minimal knee valgus, and lateral bounds with attention to hip and knee control. The agent coordinates with the biomechanical analysis agent to provide real-time feedback on landing mechanics during these exercises.

Hip strengthening exercises were performed thrice a week, including clamshells, side-lying hip abduction, monster walks with resistance bands, and single-leg deadlifts. The agent progressively increased resistance as strength improved, targeting hip abductor strength equal to or exceeding quadriceps strength.

Cutting and deceleration technique training were performed twice a week. The agent would coordinate with the biomechanical analysis agent to analyze the cutting technique, providing feedback about maintaining an upright trunk position, avoiding knee valgus and planting with the knee flexed rather than extended.

In runners, injury prevention programming addresses common overuse injury risk factors [230]. The agent would emphasize the gradual progression of running volume following the 10% rule as a general guideline [231]. It prescribes strength training targeting muscles commonly weak in injured runners, including hip abductors, hip external rotators, and calf muscles [232]. For runners showing biomechanical inefficiencies, such as overstriding, the agent would coordinate with the biomechanical analysis agent to implement gait retraining. Research has demonstrated that increasing cadence by 5–10% reduces ground reaction forces and may reduce injury risk [233].

#### 3.11.3 Coordinated Decision-Making with Multiple Agents

The theoretical injury prevention agent requires extensive coordination with other domain agents. When the injury risk increases, the injury prevention agent notifies the TLMA, which may reduce the training volume or intensity. This would notify the nutrition agent, which might increase protein intake, supporting tissue repair and recovery. It would notify the sleep agent, which might emphasize sleep optimization, given that sleep deprivation increases the risk of injury [234].

In this theoretical scenario, the injury prevention agent would synthesize the four converging indicators and estimate an approximately 3.2-fold elevation in injury risk, proposing a coordinated plan. The TLMA would concur, recommending a 25% volume reduction over seven days with temporary elimination of high-intensity plyometric activities. The biomechanical agent would attribute landing asymmetry to identified hamstring weakness and recommend concurrent gait retraining. The sleep agent would identify examination-related stress as the probable cause of sleep deficit and coordinate with the mental skills agent. The nutrition agent would increase protein allocation by 10% and emphasize anti-inflammatory dietary strategies. The injury prevention agent would compile these contributions into a single coordinated plan, transmitted with explanatory rationale to the athlete, coaching staff, and medical team, with reassessment scheduled at one week.

#### 3.11.4 Return-to-Sport Decision Support

Following injury, the injury prevention agent provides decision support for return-to-sport timing. Return-to-sport decisions involve complex clinical reasoning, considering tissue-healing status, restoration of strength and range of motion, functional performance capacity, psychological readiness, and sport-specific skill proficiency [235]. Premature return carries a substantial re-injury risk, while excessively delayed return affects athlete development and team performance.

The agent tracks progress through the rehabilitation phases. The initial phases emphasize pain and swelling management, restoring the range of motion, and preventing muscle atrophy. Intermediate phases focus on strengthening neuromuscular control and sport-specific movement patterns. The final phase emphasized the return to full training intensity and volume. The agent would have clear criteria for advancement between phases based on clinical assessment, objective testing, and functional performance assessment.

For an ACL reconstruction example, the agent would track time since surgery (minimum 6–9 months typically required [236]), quadriceps and hamstring strength (should exceed 90% of uninjured limb [237]), hop testing symmetry (single-leg hop distance, triple hop, crossover hop should all exceed 90% symmetry [238, 239]), functional movement quality (assessed through a biomechanical agent), and psychological readiness (assessed through validated questionnaires such as the ACL-Return to Sport after Injury scale [240]).

The agent made return-to-sport recommendations only when all criteria were met. If an athlete meets the strength and hop testing criteria but shows persistent biomechanical asymmetries, the agent would recommend continued rehabilitation focusing on movement quality. If an athlete meets the physical criteria but reports low psychological readiness scores indicating fear of re-injury, the agent would recommend continued rehabilitation, including psychological support, before a full return. Critically, the injury prevention agent does not make autonomous return-to-sport decisions. These decisions require human clinical judgment because of their complexity and importance. The agent provides decision support by compiling relevant information, comparing the athlete’s status against established criteria, and generating recommendations. However, final clearance must come from qualified medical professionals (i.e., team physicians, physical therapists, athletic trainers) who can consider factors beyond the agent’s data, including clinical examination findings, imaging results when obtained, and contextual factors affecting decision-making.

#### 3.11.5 Proposed Validation Approach

Validating a theoretical injury prevention agent requires demonstrating that agent-guided prevention programs reduce injury rates compared to standard care programs. RCTs compared teams or athletes receiving agent-managed injury prevention with those receiving traditional approaches. Primary outcomes included injury incidence rates, injury severity (time loss from sport), re-injury rates, and training availability. The secondary outcomes included intervention adherence, user satisfaction, and cost-effectiveness.

For example, a cluster-RCT might randomize high school soccer teams to receive either agent-managed injury prevention with coordinated interventions across multiple domains, or standard coachdirected warm-up and conditioning. Teams were followed for an entire season, with all injuries documented by certified athletic trainers Versus Standard Care. using standardized injury surveillance protocols. The primary outcome would be the injury incidence rate (i.e., injuries per 1000 athlete-exposures), with a separate analysis of different injury types (e.g., ACL injuries, ankle sprains, muscle strains). Effect sizes from previous injury prevention research provide context for the expected outcomes. Neuromuscular training programs have demonstrated ACL injury risk reductions of approximately 50% in female athletes [241]. If agent-managed comprehensive prevention approaches achieve similar or greater reductions while demonstrating acceptable usability and adherence, this would provide strong evidence to support implementation.

**FIG. 11:**
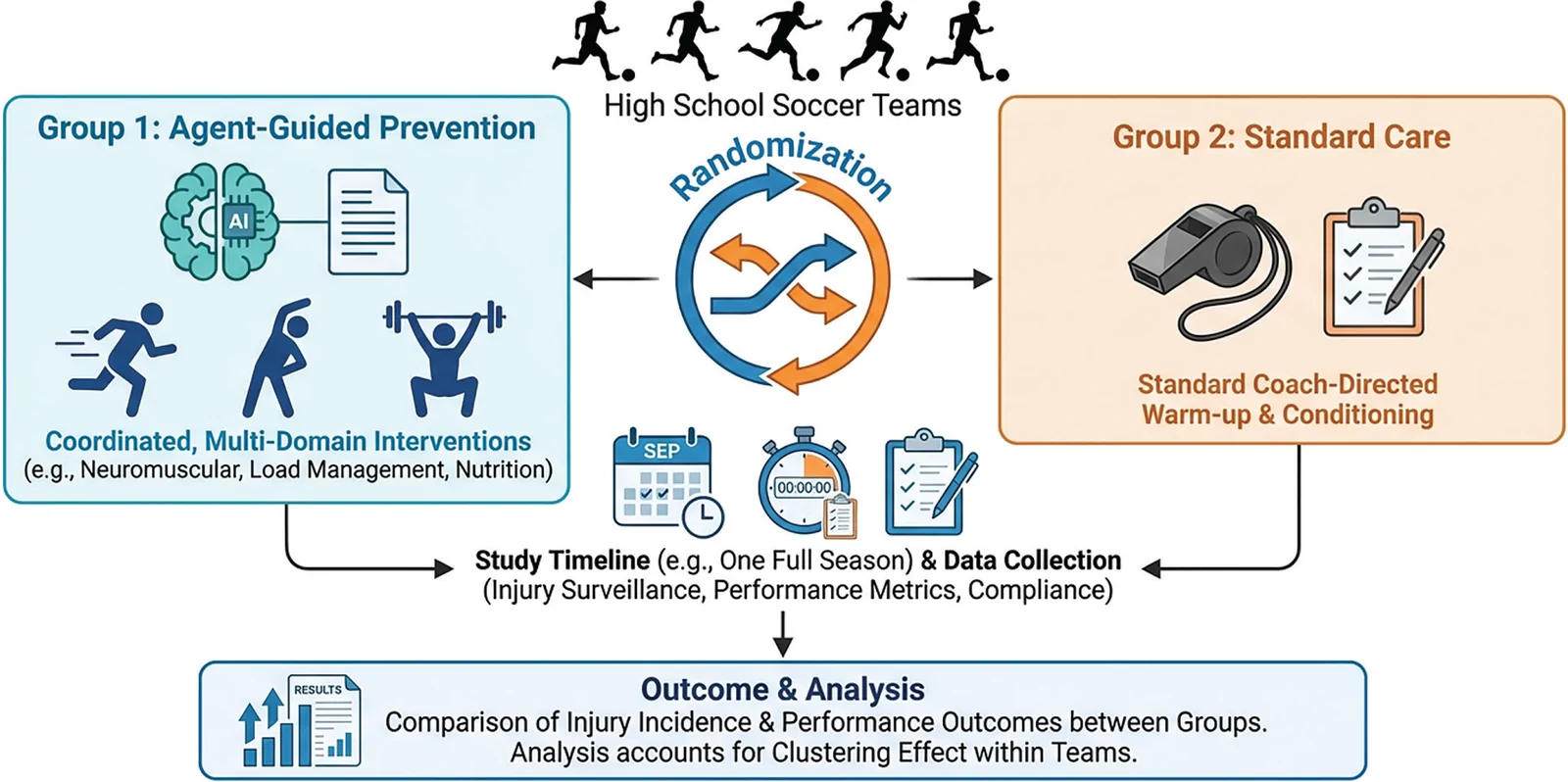
Cluster Randomized Controlled Trial Evaluating the Efficacy of an Artificial Intelligence (AI)-Powered Injury Prevention Agent Versus Standard Care.

Figure 11 outlines the methodology for validating an AI-driven injury prevention system using a cluster RCT design. High school sports teams serve as the randomization unit (clusters) to prevent intervention contamination between athletes on the same squad. The study compares an experimental arm utilizing AI-guided, coordinated, multidomain interventions against a control arm receiving standard, coachdirected conditioning. Certified athletic trainers collect data over a full season to assess primary clinical outcomes (e.g., injury incidence density per 1000 athlete-exposures, severity) and secondary implementation outcomes (e.g., adherence, cost-effectiveness). The stated goal of approximately 50% reduction in specific injury types (e.g., ACL) represents a hypothesized effect size based on pre-existing evidence for specific intervention modalities included in the coordinated program.

### 3.12 Theoretical Application Domain 7: Mental Skills Training Agent

Mental skills profoundly affect athletic performance, with research consistently demonstrating that psychological factors distinguish successful athletes from less successful ones [242, 243]. However, many athletes lack access to qualified sports psychologists who can provide systematic mental skills training. A theoretical mental skills training agent would assess psychological characteristics, provide evidence-based mental training techniques, monitor implementation and progress, and coordinate with other domain agents when psychological factors affect physical preparation or performance. All content represents theoretical proposals requiring empirical validation.

#### 3.12.1 Psychological Assessment and Profiling

The theoretical mental skills training agent would begin with a comprehensive psychological assessment identifying individual strengths and areas requiring development. Assessment would employ validated questionnaires administered through the agent interface. The “Sport Competition Anxiety Test” or “Competitive State Anxiety Inventory” assesses competitive anxiety levels [244]. The “Sport Confidence Inventory” evaluates self-confidence across multiple domains, including physical skills, cognitive efficiency, and resilience [245]. The “Athletic Coping Skills Inventory” assesses coping mechanisms, including confidence, concentration, goal setting, imagery use, and performance under pressure [246]. The “Test of Performance Strategies” evaluates the use of mental training techniques during practice and competition [247].

Beyond standardized assessments, the agents gathered qualitative information through structured interviews. It inquired about specific situations causing anxiety or performance decrements, previous experiences with mental training, motivation for sports participation, short- and long-term goals, and preferences regarding mental training approaches. This information can guide intervention selection and individualization.

The agent identifies patterns in the assessment data. Some athletes might show high competitive anxiety but strong confidence and effective coping skills, suggesting that they experience anxiety as facilitating rather than debilitating. Others might show low confidence, poor concentration abilities, and ineffective coping, suggesting the need for comprehensive mental skills development. The agent prioritizes interventions based on individual profiles and sports demands.

#### 3.12.2 Evidence-Based Mental Skills Training Interventions

The theoretical agent delivers evidence-based mental training techniques through guided exercises, instructional content, and practice assignments. Key techniques would include goal setting, imagery/ visualization, self-talk modification, arousal regulation, concentration training, and pre-performance routines.

##### 3.12.2.1 Goal Setting

Effective goal setting involves establishing specific, measurable, achievable, relevant, and time-bound objectives [248]. The agent guides athletes through organized goal-setting processes. It would help establish long-term outcome goals (e.g., qualify for national championships), intermediate performance goals (e.g., achieve specific performance standards), and short-term process goals (e.g., execute specific technical or tactical elements during competition).

Research has demonstrated that combining outcome, performance, and process goals produces better results than focusing exclusively on any single goal type [249]. The agent helps athletes establish balanced goal structures. For a swimmer targeting qualification for national championships (*outcome goal*), intermediate goals might include achieving specific race times in qualification meets (*performance goals*), whereas short-term goals might focus on start technique, turn execution, and race pacing strategy (*process goals*).

The agent implements regular goal reviews and adjustments. As athletes progress, their initial goals may require revision. Goals set too ambitiously may need to be adjusted downward to maintain motivation. Goals achieved earlier than expected may require replacement with more challenging ones. The agent schedules monthly goal review sessions, prompting athletes to evaluate their progress and adjust their goals appropriately.

##### 3.12.2.2 Imagery and Visualization

Mental imagery involves creating or recreating experiences in the mind using multiple sensory modalities [250]. Research has demonstrated that imagery practice improves skill learning, aids performance preparation, and supports injury rehabilitation [251, 252]. The agent taught methodical imagery practice through guided audio sessions.

The initial sessions would develop imagery vividness and control. The agent would guide athletes through exercises involving imaging simple objects and movements, progressively increasing the complexity. Athletes would practice incorporating multiple sensory modalities: visual (e.g., seeing the movement or environment), auditory (e.g., hearing sounds associated with the activity), kinesthetic (e.g., feeling the movement sensations), and emotional (e.g., experiencing associated feelings) modalities.

Once basic imagery skills are developed, the agent guides sportspecific imagery practice. For skill learning, athletes would image themselves performing techniques with perfect form, imagining the visual appearance, kinesthetic sensations, and successful outcome. For competition preparation, athletes imagine themselves executing their race or game plan, handling challenges that might arise, and performing successfully under pressure. The agent delivered these guided imagery sessions through audio recordings that athletes could access daily.

The agent coordinates with the biomechanical analysis agent for imagery optimization. Videos of athletes performing skills correctly would provide visual templates for imagery practice. The agent would instruct: “Watch this video of your best vault performance three times. Then close your eyes and image yourself performing the vault exactly as you see in the video, incorporating the visual appearance and the feeling of the movements”.

##### 3.12.2.3 Self-Talk Management

Self-talk refers to the internal dialogue that athletes engage in during training and competitions. Research has demonstrated that self-talk affects performance, with positive self-talk generally enhancing performance and negative self-talk undermining it [253, 254]. The agent helps athletes become aware of their self-talk patterns, identify negative or unhelpful self-talk, and develop more constructive alternatives.

The agent first increased self-talk awareness through self-monitoring exercises. Athletes completed brief questionnaires after training sessions and competitions indicating what they said to themselves during the activity, whether the self-talk was positive or negative, and whether it helped or hindered their performance. Usually, this tracking identifies trends that athletes were not aware of before.

Once patterns emerge, the agent helps athletes identify unhelpful self-talk that requires modification. Common negative patterns include catastrophizing (“If I miss this shot, everything is ruined”), harsh self-criticism (“I’m terrible at this”), and attention to irrelevant factors (“Everyone is watching me mess up”). The agent teaches cognitive restructuring techniques to replace unhelpful thoughts with more constructive alternatives.

For catastrophizing, the agent would encourage a realistic perspective: “Missing one shot does not determine the entire outcome. I’ve made many shots today and will have more opportunities”. For harsh self-criticism, the agent would promote self-compassion: “Everyone makes mistakes during learning. I’m working hard to improve”. For attention to irrelevant factors, the agent would redirect focus: “Instead of worrying about spectators, I’ll focus on my technique and execution”.

The agent provides cue words that athletes can use during performance to maintain optimal mental states. For sports requiring explosive power, cue words such as “explode”, “drive”, or “attack” can facilitate appropriate intensity. For sports requiring precision and control, cue words such as “smooth”, “steady”, or “controlled” can promote optimal execution. Athletes would practice incorporating cue words during training until they become automatic.

##### 3.12.2.4 Arousal Regulation

Optimal performance typically occurs within a specific arousal range that varies by sport and individual [255]. Sports requiring fine motor control and precision (e.g., archery, golf putting, and rifle shooting) generally require lower arousal. Sports requiring explosive power and speed (e.g., sprinting, weightlifting, and tackling in football) generally require higher arousal. The agent helps athletes understand their optimal arousal zones and develop techniques for reaching and maintaining these states.

For athletes who become over-aroused (e.g., experiencing excessive anxiety or activation), the agent teaches relaxation techniques. Progressive muscle relaxation involves systematically tensing and relaxing muscle groups to reduce physical tension [256]. Breathing exercises, such as box breathing (e.g., inhale 4 counts, hold 4 counts, exhale 4 counts, hold 4 counts) or 4-7-8 breathing, activate parasympathetic nervous system responses, promoting relaxation [257]. The agent provides guided audio instructions for these techniques, encouraging daily practice so that athletes can employ them effectively when needed before or during competition.

For athletes who become under-aroused (i.e., lacking sufficient activation and intensity), the agent would teach energizing techniques. These may include listening to high-tempo music, performing explosive movements or dynamic stretching, using activating selftalk or imagery, and engaging in brief high-intensity physical activity. The agent would help athletes identify techniques that effectively increase their arousal to optimal levels without producing anxiety.

##### 3.12.2.5 Pre-Performance Routines

Pre-performance routines are sequences of task-relevant thoughts and actions performed before skill execution [258]. Research has demonstrated that routines improve performance consistency and help athletes manage anxiety [259]. The agent helps athletes develop individualized routines appropriate for their sports.

For a basketball free throw example, an effective routine might include approaching the free throw line, receiving the ball from the referee, taking a deep breath for arousal regulation, bouncing the ball three times (consistent action providing temporal structure), visualizing the ball going through the basket, focusing on the front of the rim (attention focus), and shooting using well-practiced mechanics. The entire sequence took approximately 10–15 seconds and remained identical across all free throw attempts.

The agent guides routine development through an organized process. First, identify the performance situation requiring a routine (e.g., free throws, tennis serves, golf putts, penalty kicks). Second, break down the components of effective execution, including physical actions, attentional focus, cognitive elements, and emotional state. Third, sequence these components into a brief routine lasting 10–20 seconds for most sports. Fourth, the routine should be practiced extensively during training until it becomes automatic. Fifth, use the routine consistently during competitions.

The agent monitored routine adherence and effectiveness. It would prompt athletes to report whether they used their routines during competitions and whether they felt that the routines helped their performance. For routines that are ineffective or cumbersome, the agent guides the refinement. Research indicates that routines should feel natural and automatic rather than effortful, suggesting the need for simplification when athletes report routines feeling forced or distracting [260].

#### 3.12.2.6 Coordination with Other Domain Agents

The theoretical mental skills agent would coordinate with other agents when psychological factors affect physical preparation or performance of the athlete. When a sleep agent detects poor sleep quality, it may inquire whether anxiety or racing thoughts prevent sleep onset. If so, it would notify the mental skills agent, which would provide relaxation and cognitive techniques specifically to improve sleep onset. The mental skills agent might teach thoughtstopping techniques (mentally saying “stop” when worrying begins, then redirecting attention to relaxing imagery [261]) or cognitive defusion techniques [262] from acceptance and commitment therapy.

When the TLMA must reduce training due to injury risk or accumulated fatigue, the mental skills agent would help athletes cope with the frustration of modified training. Many highly motivated athletes struggle emotionally with reduced training and experience anxiety about fitness losses or falling behind competitors. The mental skills agent would normalize these feelings, provide perspective on the temporary nature of recovery periods, and help athletes maintain mental engagement during physical recovery through mental imagery practice of their sports skills.

When the injury prevention agent identifies an athlete recovering from injury and approaching return to sport, the mental skills agent assesses and addresses psychological readiness. Fear of reinjury commonly affects athletes following significant injuries, sometimes persisting even after full physical recovery [263]. The agent implements graded exposure approaches, where athletes progressively engage in increasingly challenging activities while using anxiety management techniques. It would provide cognitive restructuring for catastrophic thinking about the probability of reinjury. Imagery is used to build confidence in the injured body part’s capacity to handle sports demands.

#### 3.12.3 Proposed Validation Approach

Validating a theoretical mental skills training agent requires demonstrating that agent-delivered interventions improve both psychological characteristics and performance outcomes compared to no intervention or standard education. RCTs should be conducted to compare athletes who receive mental skills training delivered by agents with those in waitlist control groups or attention control groups that receive generic sports psychology education without systematic skills training. Primary outcomes would include changes in psychological characteristics assessed through validated questionnaires (e.g., anxiety, confidence, concentration, coping skills), intervention adherence (e.g., completion of assigned practice exercises), and most critically, performance outcomes in sport-specific tests or competitions. Secondary outcomes included user satisfaction, perceived helpfulness of specific techniques, and continued use of techniques beyond the intervention period.

For example, a trial might randomize collegiate athletes to receive either 12 weeks of agent-delivered mental skills training (including assessment, personalized intervention selection, guided practice sessions, and progress monitoring) or a control condition. Mental skills can be assessed at baseline, mid-intervention (6 weeks), post-intervention (12 weeks), and follow-up (24 weeks). Performance can be assessed through competition results during the season. The hypothesis predicts that athletes receiving agent-delivered training will show greater improvements in mental skills and competitive performance than the controls.

Qualitative research would explore athlete experiences with agentdelivered training, identifying which components athletes find most valuable, barriers to consistent practice implementation, and suggestions for improving the delivery methods. This information guides iterative agent refinement.

**FIG. 12:**
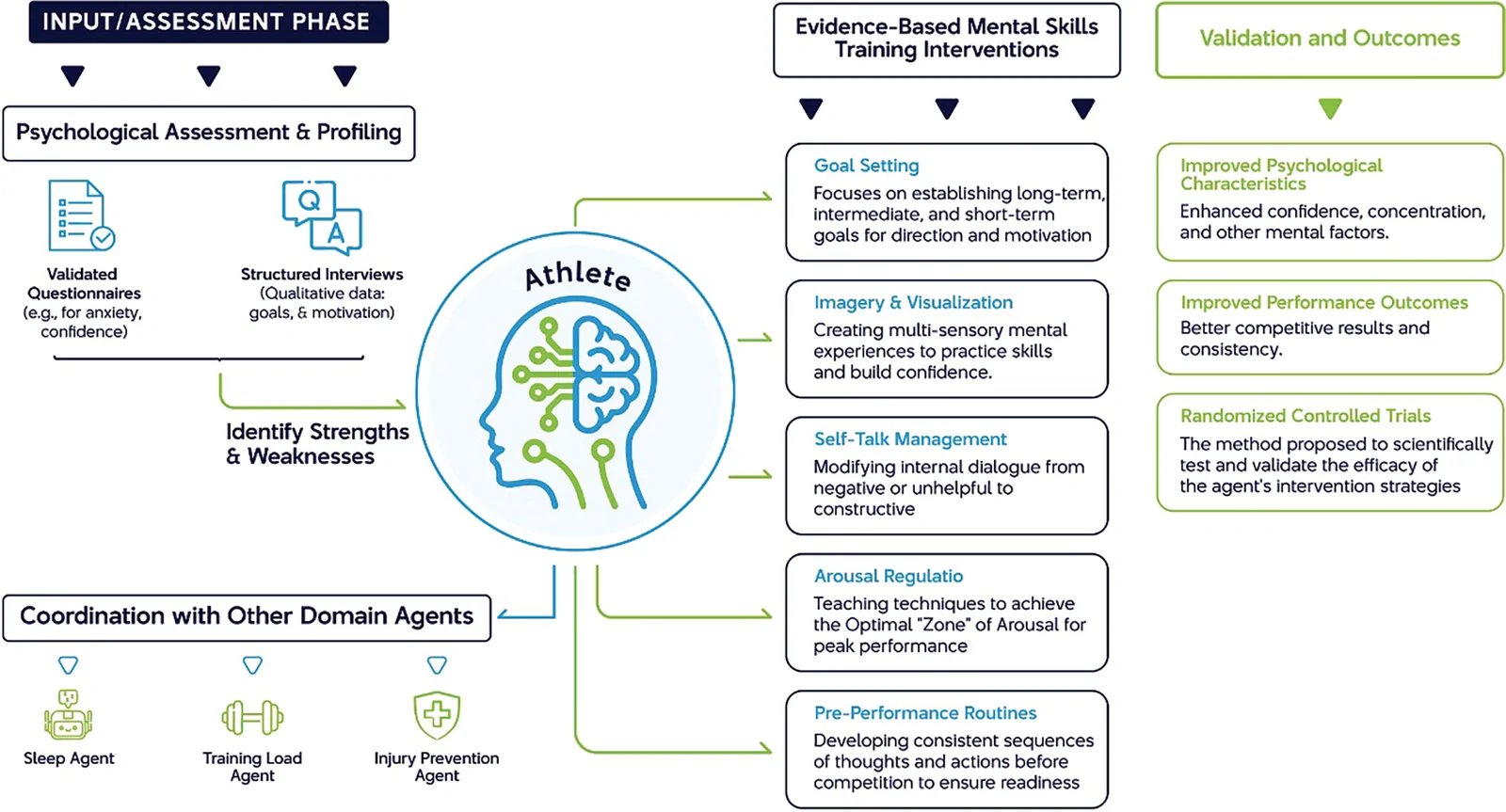
Mental Skills Training Agent: A Flowchart of Psychological Assessment, Intervention, and Integrated Outcomes.

Figure 12 illustrates the comprehensive process of a Mental Skills Training Agent, beginning with psychological assessment to identify an athlete’s strengths and weaknesses. It then details evidence-based mental skills training interventions, including goal setting, imagery, self-talk management, arousal regulation, and pre-performance routines. The figure also highlights the agent’s coordination with other domain agents for holistic athlete support and concludes with the validation methods and desired outcomes such as improved psychological characteristics and performance.

### 3.13 Theoretical Application Domain 8: Rehabilitation Progression Agent

Rehabilitation following musculoskeletal injury represents a complex clinical domain that requires organized progression through phases targeting different recovery objectives. A theoretical rehabilitation progression agent would monitor recovery status through objective assessments, guide exercise prescription through rehabilitation phases, determine readiness for phase advancement, coordinate with other domain agents during recovery, and support the eventual return to full activity. All content represents theoretical proposals requiring empirical validation before implementation.

#### 3.13.1 Rehabilitation Phase Structure and Progression Criteria

Rehabilitation typically progresses through distinct phases, each with specific objectives and progression criteria [264, 265]. The theoretical agent would manage the progression through these phases based on objective assessment data.

##### 3.13.1.1 Phase 1: Acute/Protection Phase

This initial phase, immediately following injury or surgery, emphasizes the protection of healing tissues, pain and swelling control, muscle atrophy prevention, and maintenance of the range of motion in unaffected joints. The duration varies by injury severity but typically lasts 1–2 weeks for minor injuries and 4–6 weeks following surgical procedures [266].

The agent prescribes interventions appropriate for this phase, including initial injury management guided by contemporary frameworks: protection, elevation, avoidance of anti-inflammatory modalities, compression, and education (PEACE) followed by load, optimism, vascularization, and exercise (LOVE) [267] are recommended; the RICE (Rest, Ice, Compression, Elevation) protocol [268] for initial injury management; gentle range of motion exercises within pain-free ranges to prevent stiffness; isometric muscle contractions to minimize atrophy without stressing healing tissues; and cardiovascular exercise using unaffected body parts to maintain general fitness. For an ACL reconstruction example, *Phase 1* interventions would include immediate post-operative pain and swelling management, heel slides and knee flexion/extension exercises to restore range of motion with a goal of achieving 0–90 degrees within 2 weeks, quadriceps setting exercises and straight leg raises to activate muscles without stressing the graft, and upper body and contralateral leg cardiovascular exercise. Progression criteria from *Phase 1* to *Phase 2* would include well-controlled pain and swelling (e.g., patient-reported pain below 3/10 on the visual analog scale), range of motion approaching normal (e.g., knee flexion ≥ 90° and extension to 0° for ACL example), adequate muscle activation (e.g., ability to perform straight leg raise without quadriceps lag), and physician clearance based on the tissue healing timeline.

##### 3.13.1.2 Phase 2: Subacute/Strengthening Phase

This phase emphasizes progressive strengthening, continued range of motion restoration toward full normal ranges, and the introduction of functional movements. The duration typically spans 4–8 weeks [269].

The agent prescribed progressive resistance exercises with gradually increasing loads, functional movement patterns relevant to the individual’s activity goals, proprioceptive and balance training, and sport- or activity-specific movements at reduced intensities. For ACL reconstruction, *Phase 2* would include progressive strengthening exercises (e.g., leg press, step-ups, hamstring curls, hip strengthening), balance training (e.g., single-leg stance progressions on firm and unstable surfaces), and the introduction of controlled lunges and squats.

Progression criteria from *Phase 2* to *Phase 3* would include strength deficits reduced to less than 20% compared to the uninjured side [270], full pain-free range of motion, demonstrated proprioceptive control (single-leg balance tests), and the ability to perform functional movements with proper form.

##### 3.13.1.3 Phase 3: Advanced Strengthening and Return to Activity Phase

This phase emphasizes achieving strength symmetry, developing sport-specific skills, progressing to higher-intensity activities, and building confidence in the injured body part’s capacity. The duration spans several weeks to months, depending on the injury severity and activity demands [271].

The agent would prescribe high-level strengthening exercises approaching sport-specific demands, plyometric exercises progressing from bilateral to unilateral and from low to high intensity, agility and sport-specific drills, and gradual return to practice activities with appropriate volume and intensity progressions. For ACL reconstruction, *Phase 3* would include advanced strengthening (e.g., single-leg squats, Bulgarian split squats, Nordic hamstring exercises), progressive plyometrics (e.g., box jumps, broad jumps, single-leg hops), cutting and pivoting drills with progressive intensity, and a controlled return to sport-specific training.

Progression from *Phase 3* to full return to sport requires meeting comprehensive criteria, including strength symmetry ≥ 90% between limbs [239, 272], hop test symmetry ≥ 90% across multiple tests [273], biomechanical symmetry during functional movements (assessed by a biomechanical agent), psychological readiness (assessed by a mental skills agent), completion of progressive sportspecific training without setbacks, and medical clearance from qualified clinicians.

#### 3.13.2 Objective Monitoring and Data-Driven Progression

The theoretical rehabilitation agent would employ objective assessments to determine phase advancement readiness rather than relying solely on time-based protocols. While tissue healing timelines provide important constraints (e.g., cannot progress to high-impact activities before tissues have adequately healed), within these constraints, progression should be based on the demonstrated capacity rather than arbitrary time points [274]. Most-updated evidence confirms that even at standard rehabilitation timepoints most athletes fail to meet objective strength criteria, underscoring the inadequacy of time-based protocols and validating the criteria-driven progression approach proposed here [274].

The agent integrates data from multiple sources. Strength testing through handheld dynamometry or isokinetic testing can quantify force production capacity and symmetry. Range of motion measurements through goniometry or motion capture can document flexibility restoration. Functional tests, including hop tests, balance assessments, and movement quality screens, were used to evaluate dynamic capacity. Patient-reported outcomes through validated questionnaires would capture subjective function, pain, and psychological readiness. Biomechanical analysis through motion capture or video analysis can identify persistent movement compensations that require attention.

For a practical example, consider an athlete who is 4 months post-ACL reconstruction. Traditional time-based protocols might advance athletes to return-to-sport training based solely on the 4-month timeline. However, objective assessment revealed quadriceps strength at only 75% of the uninjured limb, well below the 90% criterion. Hop test symmetry averaged 82%, which was also below the criteria. The rehabilitation agent would determine that despite adequate healing time, the athlete has not yet developed sufficient physical capacity for a safe return to the sport. *Phase 3* strengthening and functional training would continue, with reassessment every 2 weeks until the criteria were met. Conversely, another athlete at 4 months post-ACL reconstruction demonstrated quadriceps strength at 92% of the uninjured limb, hop test symmetry at 94%, and excellent movement quality. This athlete meets the physical criteria ahead of the typical timeline. However, psychological readiness assessment revealed low confidence scores and high fear of re-injury. The rehabilitation agent would coordinate with the mental skills agent to address psychological barriers before full return to sports clearance, recognizing that psychological readiness is as important as physical capacity [275].

#### 3.13.3 Exercise Modification Based on Pain and Response

The theoretical rehabilitation agent implements sophisticated pain monitoring and exercise modification algorithms. Pain during rehabilitation requires careful consideration. Some discomfort during exercise is acceptable and may indicate appropriate tissue loading, promoting adaptation. However, excessive pain suggests inappropriate loading, which is potentially harmful to healing tissues [276].

The agent implements a traffic light system for pain monitoring [277]. *Green* indicates acceptable pain (i.e., mild discomfort during exercise that resolves within a few hours, discomfort rated 3/10 or less on pain scales, and no increase in pain, swelling, or stiffness on the following day). *Yellow* indicates concerning pain (i.e., moderate pain during exercise rated 4–6/10, pain persisting several hours after exercise, or mild increases in pain, swelling, or stiffness the following day). *Red* indicates unacceptable pain (i.e., severe pain during exercise rated 7/10 or higher, sharp or catching sensations suggesting mechanical issues, significant increases in pain or swelling persisting for 24+ hours, or any pain affecting movement quality).

When athletes report green-light pain, the agent continues the current program with normal progression. Yellow-light pain would trigger exercise modifications, including reduced load (weight, repetitions, or sets), increased rest intervals between sets, substituting alternative exercises targeting the same goals, or temporarily reducing exercise frequency. Red-light pain triggers session termination, urgent notification to supervising clinicians, temporary cessation of aggravating activities, and medical evaluation before resuming rehabilitation.

The agent learns from individual pain responses over time. Some athletes are more pain-sensitive and consistently report higher pain ratings for given activities. Others are less sensitive. The agent calibrates its pain interpretation to individual baseline tendencies while maintaining conservative safety margins.

#### 3.13.4 Coordination with Training Load and Performance Agents

During rehabilitation, athletes often continue training uninjured body parts to maintain their fitness. The theoretical rehabilitation agent would coordinate closely with the TLMA to ensure that the overall stress remains appropriate. An athlete rehabilitating a knee injury may perform intensive upper body training. The rehabilitation agent communicates the loading of the injured limb to the TLMA, which calculates the total body stress considering both rehabilitation exercises and supplementary training.

When the rehabilitation agent determines that an athlete has progressed sufficiently to return to modified sport-specific training, it would work with the TLMA to implement gradual exposure progressions. Research has demonstrated that graduated return-to-sport protocols reduce re-injury risk compared to abrupt returns to full training [278]. The agents would coordinate a progressive plan: week 1 might include 25% of the normal training volume with no high-intensity activities. In week 2, 50% of the volume may be achieved with the introduction of low-intensity sport-specific drills. In week 3, the volume may progress to 75% with moderate-intensity activities. In week 4, full volume with all activities may be allowed if tolerated well.

The rehabilitation agent would continuously monitor for warning signs of excessive loading, including increased pain or swelling, declining strength or range of motion, compromised movement quality identified by the biomechanical agent, or patient-reported increases in concern about the injury. Any concerning indicators would trigger discussions between agents about temporarily reducing progression rates.

#### 3.13.5 Patient Education and Adherence Support

Rehabilitation adherence is critical for successful outcomes but remains challenging. Studies indicate that 40–65% of patients demonstrate poor adherence to home exercise programs [279, 280]. The theoretical rehabilitation agent implements multiple strategies to support adherence. First, the agent provides comprehensive education about injury mechanisms, healing processes, rehabilitation rationales, and expected timelines. Research has demonstrated that patient education improves adherence and outcomes [281]. The agent would explain in accessible language: “Your ACL graft is currently in the remodeling phase, where new collagen is being laid down. The strengthening exercises we’re doing provide the mechanical stimulus needed for optimal collagen alignment, which will make your knee stronger and more stable”. Second, the agent would deliver exercises through multimodal instruction, including detailed text descriptions, video demonstrations from multiple angles, common error warnings, and real-time form feedback through the biomechanical analysis agent when video analysis is available. This multimodal approach accommodates different learning preferences and improves adherence to proper techniques. Third, the agent implements structured goal-setting for rehabilitation. Rather than vague goals like “get stronger”, the agent would establish specific milestones: “Achieve quadriceps strength within 10% of your uninjured leg by 6 weeks from now”. It would break long rehabilitation timelines into shorter achievement periods with clear objectives, providing a sense of progress and accomplishment. Fourth, the agent uses motivational interviewing principles to address adherence barriers [282]. When adherence declines, the agent would inquire about obstacles: “I notice you have completed only two of your last seven scheduled exercise sessions. What challenges are you experiencing with your rehabilitation program?” Based on the responses, the agent would collaboratively problem-solve. If time proves problematic, it might condense programs to essential exercises only. If exercise locations prove difficult, it might substitute home-based alternatives may be considered. If boredom is an issue, exercise variations can be introduced while maintaining similar benefits. Fifth, the agent provides positive reinforcement for adherence and progress. When patients consistently completed the prescribed exercises, the agent acknowledged this by saying, “Excellent adherence this week! You have completed all seven scheduled sessions. This consistency will accelerate your recovery”. When objective assessments show improvement, the agent would highlight progress: “Your knee flexion has improved from 95° to 115° over the past 2 weeks. This represents excellent progress and indicates your exercises are working well”.

#### 3.13.6 Special Considerations for Different Injury Types

The rehabilitation agent adapts its approach based on the injury characteristics. Acute injuries (e.g., sudden onset, typically from traumatic events) and overuse injuries (e.g., gradual onset from repetitive microtrauma) require different management approaches [283]. Surgical and conservative management alter rehabilitation timelines and protocols. Soft tissue injuries (e.g., muscles, tendons, ligaments) and bony injuries have different healing characteristics and constraints.

For conservatively managed Achilles tendon ruptures (without surgery), the agent implemented progressive loading protocols. In the early phases, isometric contractions are performed without lengthening the tendon. Middle phases would introduce eccentric exercises (heel drops) shown to promote optimal collagen alignment [284]. Late phases would progress to plyometric exercises and running. The agent would closely monitor for signs of tendon irritation including localized pain, morning stiffness, or tendon thickening reported by the patient, as excessive loading during healing can produce inferior outcomes [285].

For rotator cuff repairs, the agent would respect tissue-healing constraints that differ from lower extremity injuries. Initial phases would maintain the arm in protective positions avoiding stress to surgical repairs. Range of motion restoration would proceed cautiously, with external rotation particularly restricted in early phases to protect healing tissues [286]. Strengthening would not begin until tissue healing achieves adequate integrity, typically 6–8 weeks post-operatively. The agent would coordinate with the biomechanical analysis agent to ensure shoulder movements avoid excessive strain on healing structures.

For concussions, rehabilitation differs fundamentally from musculoskeletal injuries. The agent would implement graduated returnto-activity protocols beginning with rest, progressing through light aerobic exercise, sport-specific training, non-contact practice, and finally full return to sport, with advancement only when athletes remain asymptomatic at each level [287]. The agent would monitor symptom reports carefully, as symptom exacerbation indicates inadequate recovery requiring return to previous levels. It would coordinate with the sleep agent, as sleep disturbances commonly follow concussions and affect recovery [288]. It would coordinate with the mental skills agent, as cognitive symptoms (e.g., difficulty concentrating, memory problems) often require specific management strategies.

#### 3.13.7 Proposed Validation Approach

Validating a theoretical rehabilitation progression agent requires demonstrating that agent-managed rehabilitation produces outcomes equal to or better than traditional clinician-managed rehabilitation. RCTs would compare patients receiving agent-managed rehabilitation (with clinician oversight for complex decisions) versus traditional face-to-face rehabilitation with the same clinician contact frequency.

Primary outcomes would include time to achieve return-to-activity clearance, objective functional outcomes (e.g., strength, range of motion, hop tests), patient-reported outcome measures (e.g., subjective function, pain, satisfaction), re-injury rates during the 12 months following return to activity, and healthcare utilization (e.g., clinic visits, additional interventions required).

For an ACL reconstruction population, a trial might randomize patients to receive either agent-managed rehabilitation with weekly clinician check-ins or traditional twice-weekly in-person rehabilitation sessions. Both groups would receive equivalent total clinician contact time but distributed differently. The hypothesis would predict that agent-managed rehabilitation produces non-inferior outcomes to traditional rehabilitation while potentially offering advantages in adherence (due to continuous monitoring and feedback), consistency (due to standardized progression criteria), and patient satisfaction (due to convenience of home-based exercise with remote monitoring).

Economic evaluation would assess cost-effectiveness considering direct rehabilitation costs, indirect costs (e.g., patient time, travel), and downstream costs (e.g., complications, re-injuries, additional procedures). If agent-managed rehabilitation produces similar outcomes at lower costs, this would support widespread implementation, particularly in underserved populations with limited access to rehabilitation facilities. Growing recognition that AI-assisted personalized guidance may improve rehabilitation adherence and neuroplastic outcomes in clinical populations [289] lends independent external support to the rehabilitation agent framework proposed here and suggests broader applicability beyond musculoskeletal injury recovery.

**FIG. 13:**
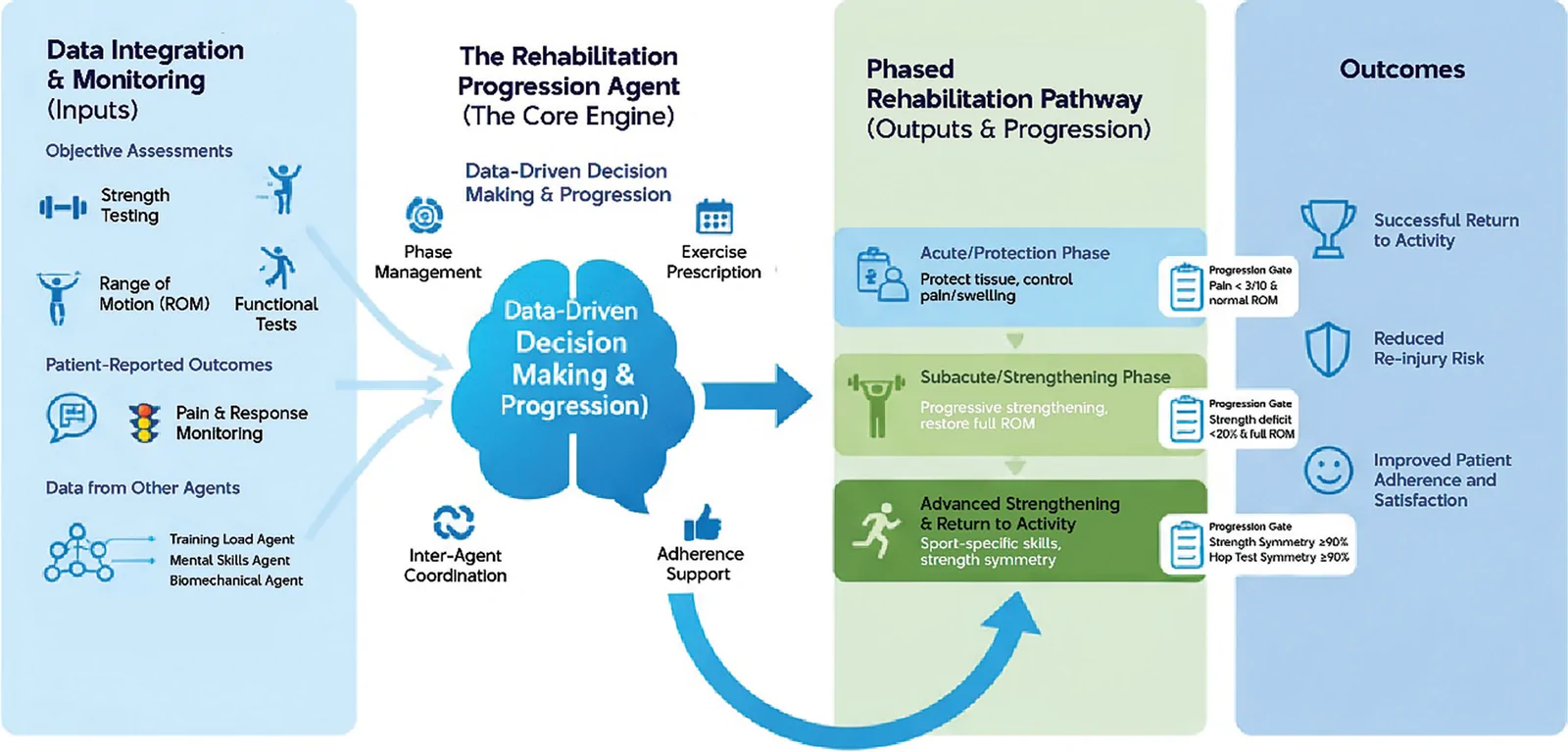
An Artificial Intelligence-Driven Agent for Personalized Rehabilitation Progression.

Figure 13 illustrates the comprehensive function of the Rehabilitation Progression Agent, an AI-driven system that integrates diverse patient data to facilitate data-driven decision-making. It guides individuals through a phased rehabilitation pathway, from acute protection to advanced strengthening and return to activity, ensuring personalized progression through defined gates and ultimately leading to successful outcomes like reduced re-injury risk and improved patient satisfaction, all within a continuous monitoring and adjustment feedback loop.

### 3.14 Multi-Agent Coordination Protocols and Technical Architecture

Once specialized agents demonstrate validated performance within their individual domains, developing coordination protocols enabling collaborative operation becomes essential for comprehensive athlete management. Multi-agent coordination introduces substantial technical complexity but provides corresponding benefits through integrated decision-making, reduced conflicting recommendations, and holistic athlete support. This section examines theoretical coordination architectures and protocols. All content represents proposals requiring implementation and validation.

#### 3.14.1 Centralized Versus Decentralized Coordination Architectures

Two primary architectural approaches exist for multi-agent coordination: centralized systems with a coordination hub managing interagent communication, and decentralized systems where agents communicate directly through peer-to-peer protocols [290, 291]. Each approach offers distinct advantages and disadvantages.

*Centralized architecture* employs a coordination hub that all agents connect to and communicate through. The hub maintains system-wide state information, manages message routing between agents, detects patterns across domains that individual agents might miss, orchestrates collective responses to complex situations, and enforces priority rules when conflicts arise. Advantages of centralized architecture include simplified communication protocols (i.e., agents only need to communicate with the hub rather than every other agent), easier implementation of system-wide constraints and priorities, comprehensive logging and monitoring of all inter-agent communication facilitating debugging and oversight, and pattern detection across domains that individual agents cannot observe. Disadvantages include the hub becoming a single point of failure (if the hub malfunctions, all coordination ceases), potential communication bottlenecks when many agents generate high message volumes, increased system complexity requiring the hub to understand all domain-specific information, and reduced resilience (i.e., individual agents cannot continue coordinating if the hub fails).

*Decentralized Architecture* allows agents to communicate directly with each other through standardized peer-to-peer protocols without central coordination hubs. Each agent maintains awareness of which other agents exist and how to contact them. When coordination is needed, agents communicate directly. Advantages of decentralized architecture include no single point of failure (i.e., system continues functioning if individual agents malfunction), greater scalability (i.e., adding new agents doesn’t overburden a central hub), simpler hub implementation (or no hub required), and greater resilience (i.e., agents can continue coordinating even if some system components fail). The disadvantages include the heightened complexity of communication protocols, as each agent must possess the capability to interact with multiple other agents. Furthermore, there is a challenge in enforcing system-wide priorities because of the lack of a central authority to determine which agent’s recommendations should take precedence. Furthermore, challenges arise in detecting patterns that require information from multiple domains, and monitoring and debugging processes are complicated by communication occurring through multiple channels rather than being routed through a single, observable central point.

For sports science applications, we propose a *hybrid architecture* combining centralized and decentralized elements. A lightweight coordination hub handles message routing, logging, and pattern detection across domains but does not make decisions itself. Agents communicate through the hub for visibility and monitoring but can also establish direct peer-to-peer connections for routine coordination requiring rapid responses. This hybrid approach balances the advantages of both architectures while mitigating their disadvantages.

**FIG. 14:**
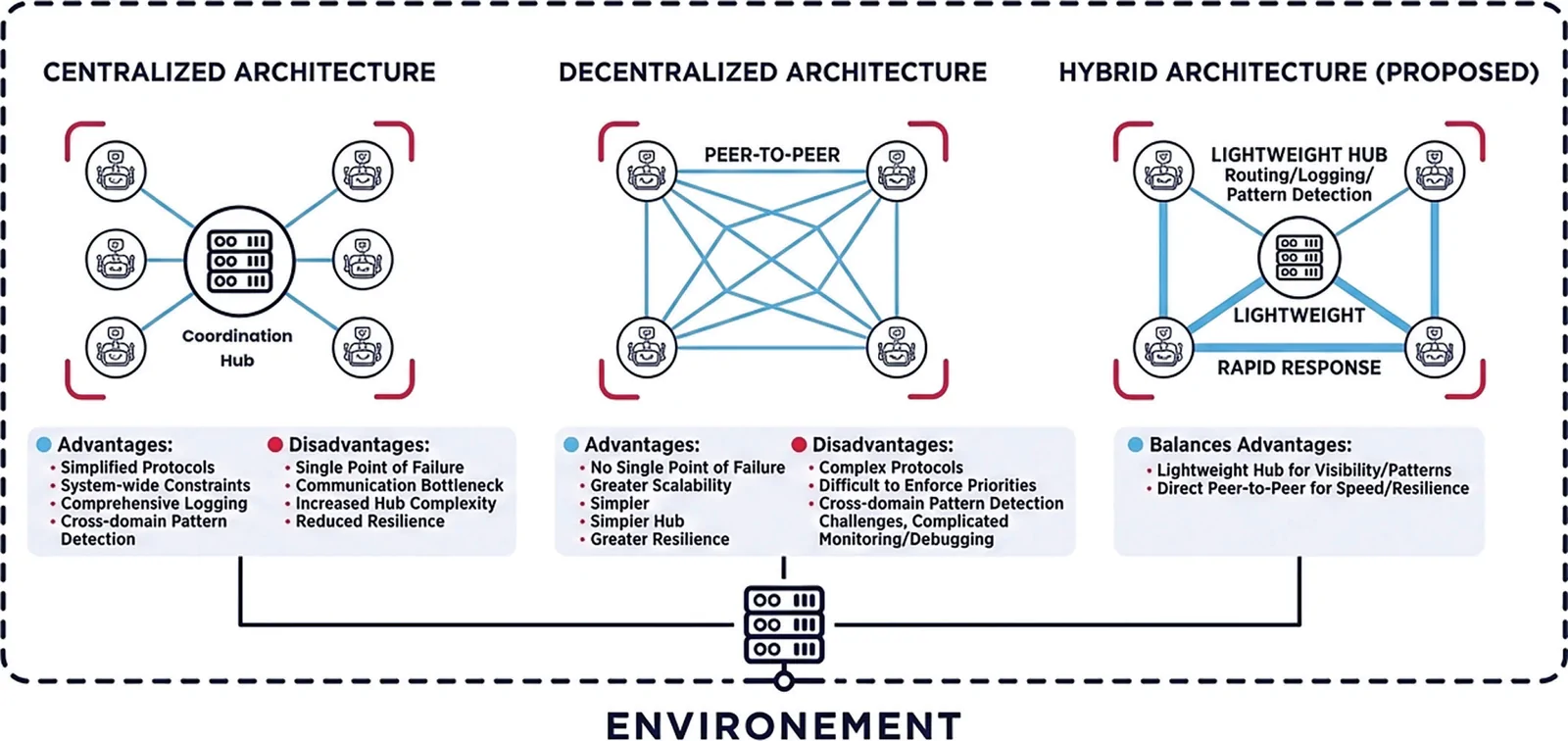
Architectural Classifications of Advanced Artificial Intelligence Agents.

Figure 14 illustrates the three major architectural categories for designing AI agents: *Hybrid Architecture* (combining rapid response and complex planning), *Multi-Agent Systems* (where several agents cooperate or compete for shared goals), and *Hierarchical Agents* (where a high-level agent delegates and orchestrates tasks to specialized low-level agents). These agents interact within an Environment.

#### 3.14.2 Standardized Communication Protocol Development

Effective multi-agent coordination requires standardized protocols specifying message formats, required information fields, and response expectations. We propose a hierarchical message structure supporting different coordination scenarios.

Information-sharing messages represent the most basic coordination type, in which an agent notifies others of changes relevant to their domain without requiring an explicit response. For example, a TLMA notifying of an upcoming reduced training week would transmit the athlete identifier, current training phase, projected load reduction percentage, and duration, allowing the nutrition and sleep agents to adjust their recommendations autonomously upon receipt.

**Figure:**
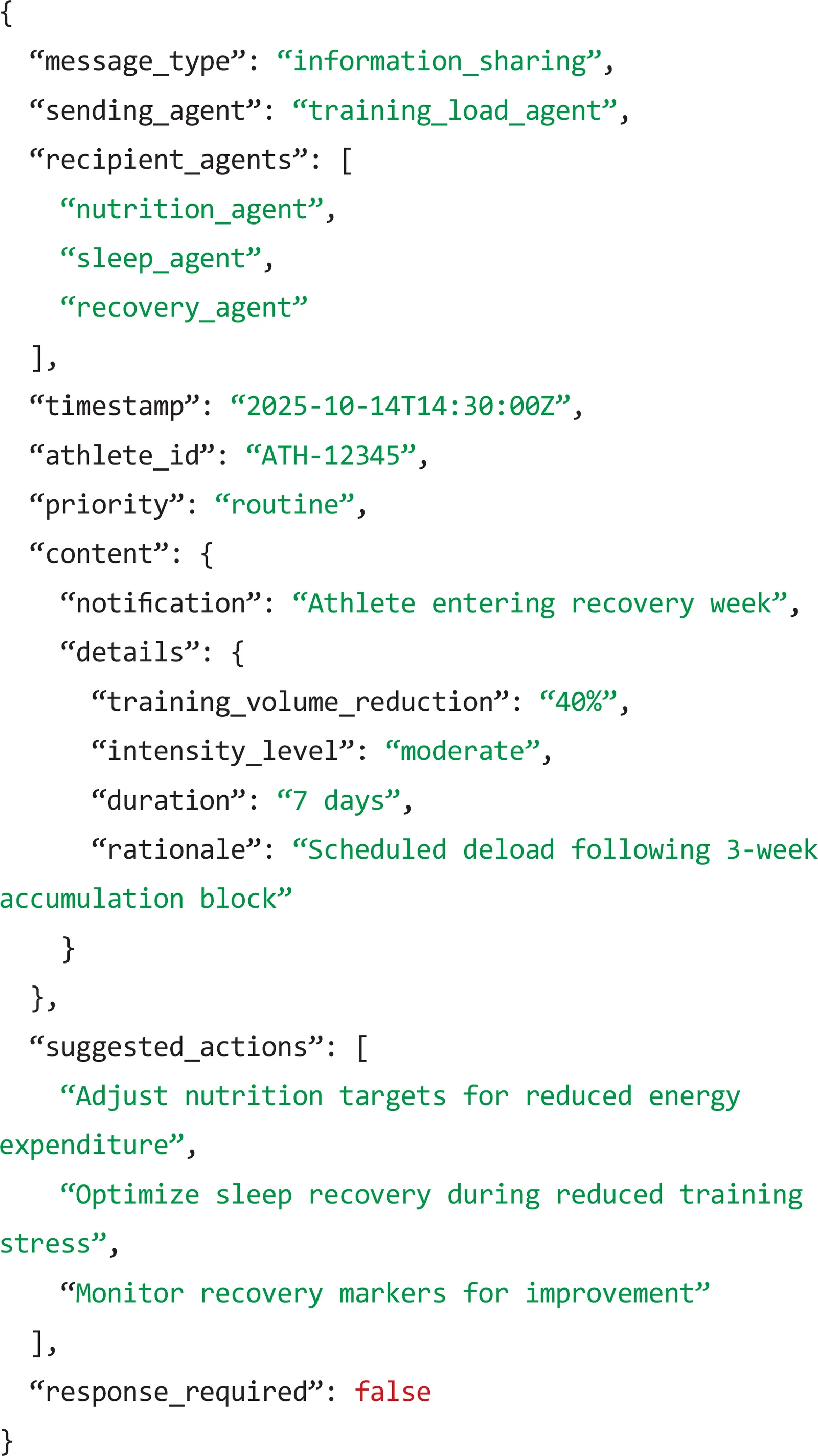


This message informs relevant agents about upcoming reduced training without requiring explicit responses. The nutrition agent receives this notification and automatically reduces daily energy targets by approximately 15–20% to match reduced expenditure while maintaining protein intake for tissue maintenance. The sleep agent notes that reduced training stress should facilitate improved sleep quality and will monitor whether this occurs. The recovery agent recognizes this represents an opportunity to assess whether the previous training block produced appropriate adaptation or accumulated excessive fatigue.

**Request for information messages** occur when an agent needs data from another agent’s domain to make optimal decisions. Message structure would include:

**Figure:**
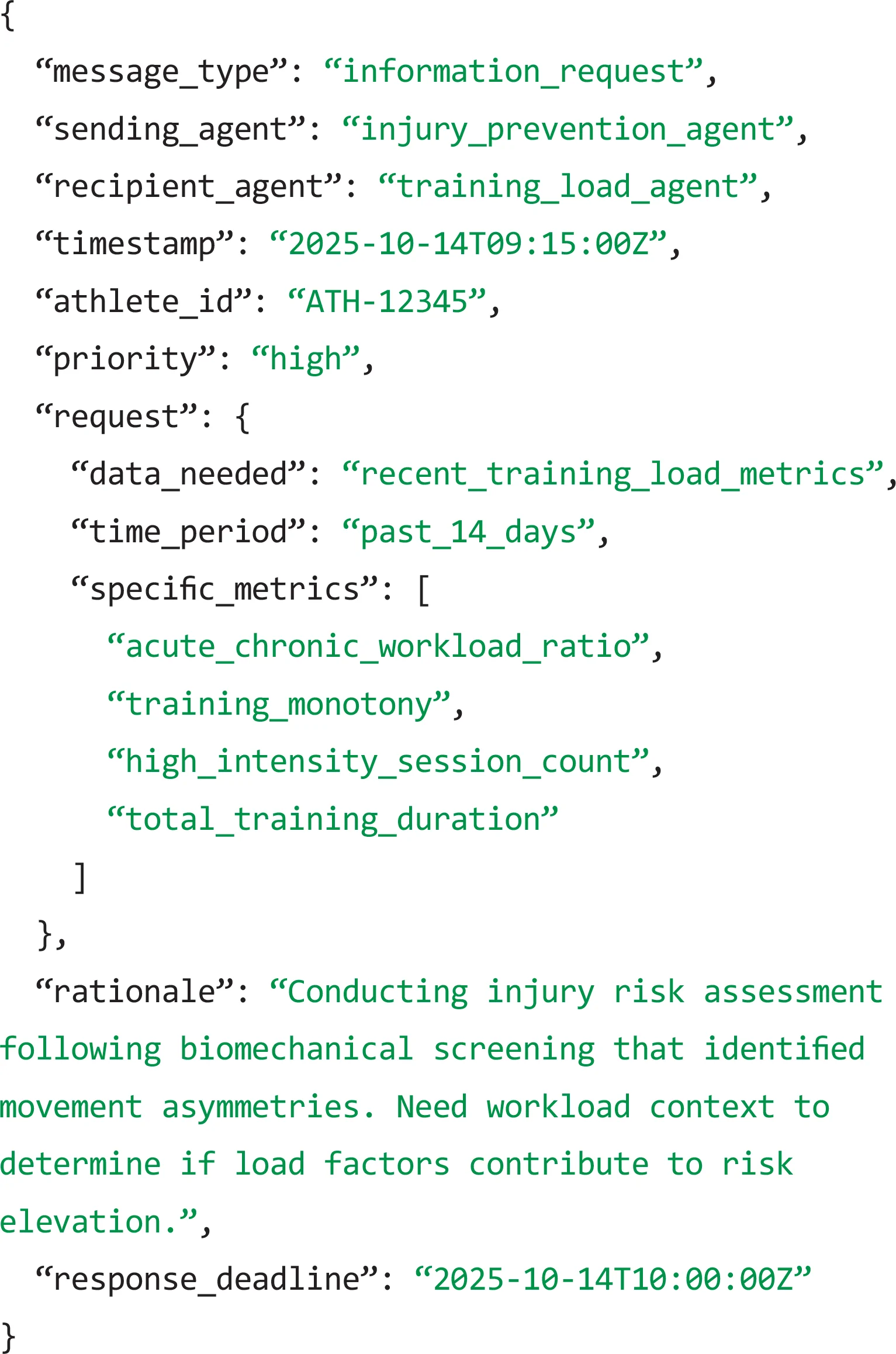


The TLMA receives this request, retrieves the requested data, and responds:

**Figure:**
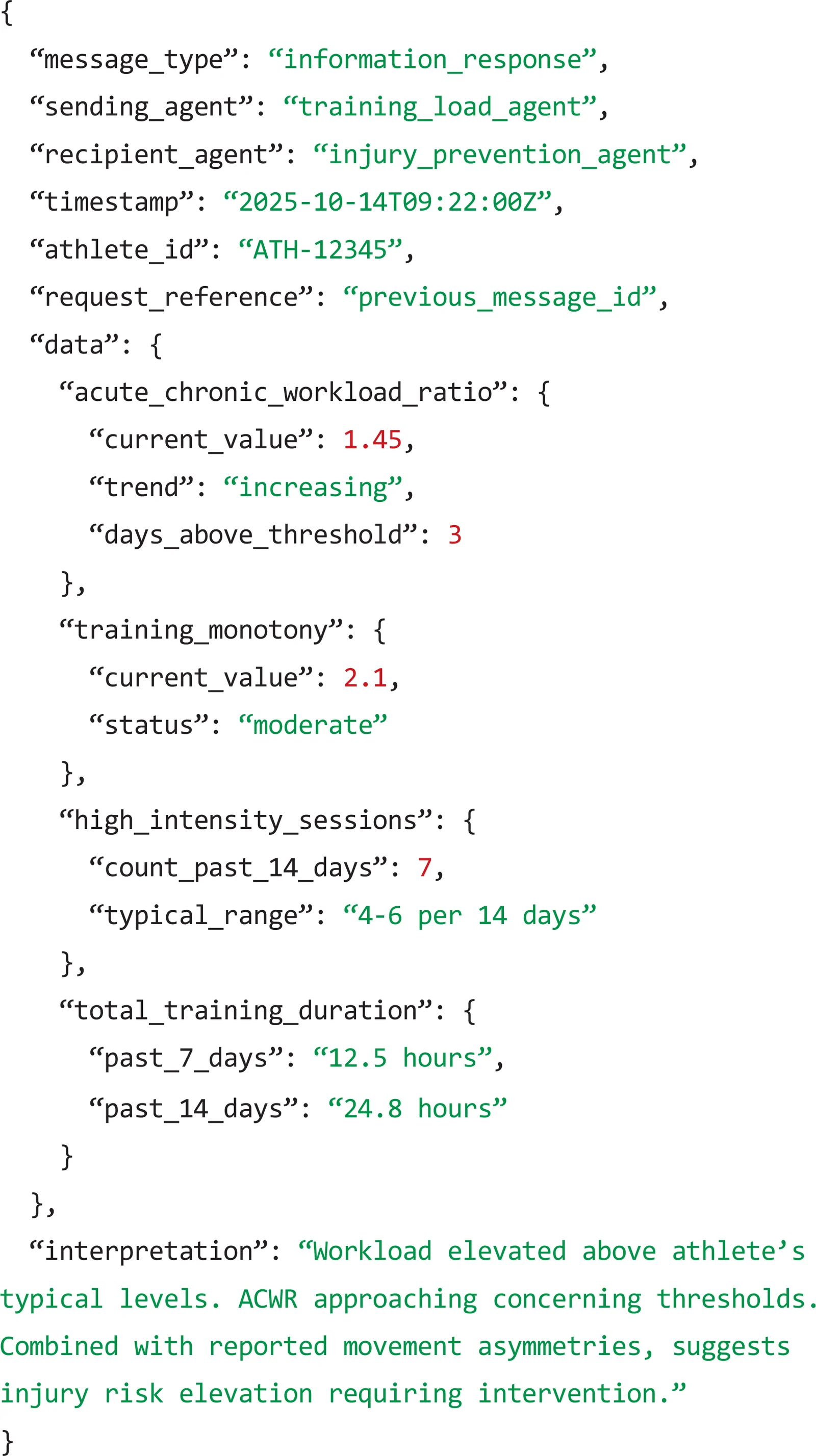


The injury prevention agent integrates this workload data with biomechanical assessment results, determining that converging risk factors warrant intervention. It initiates a coordination request message to multiple agents.

**Coordination request messages** occur when an agent’s proposed action might affect other domains and coordination proves beneficial. Message structure would include:

**Figure:**
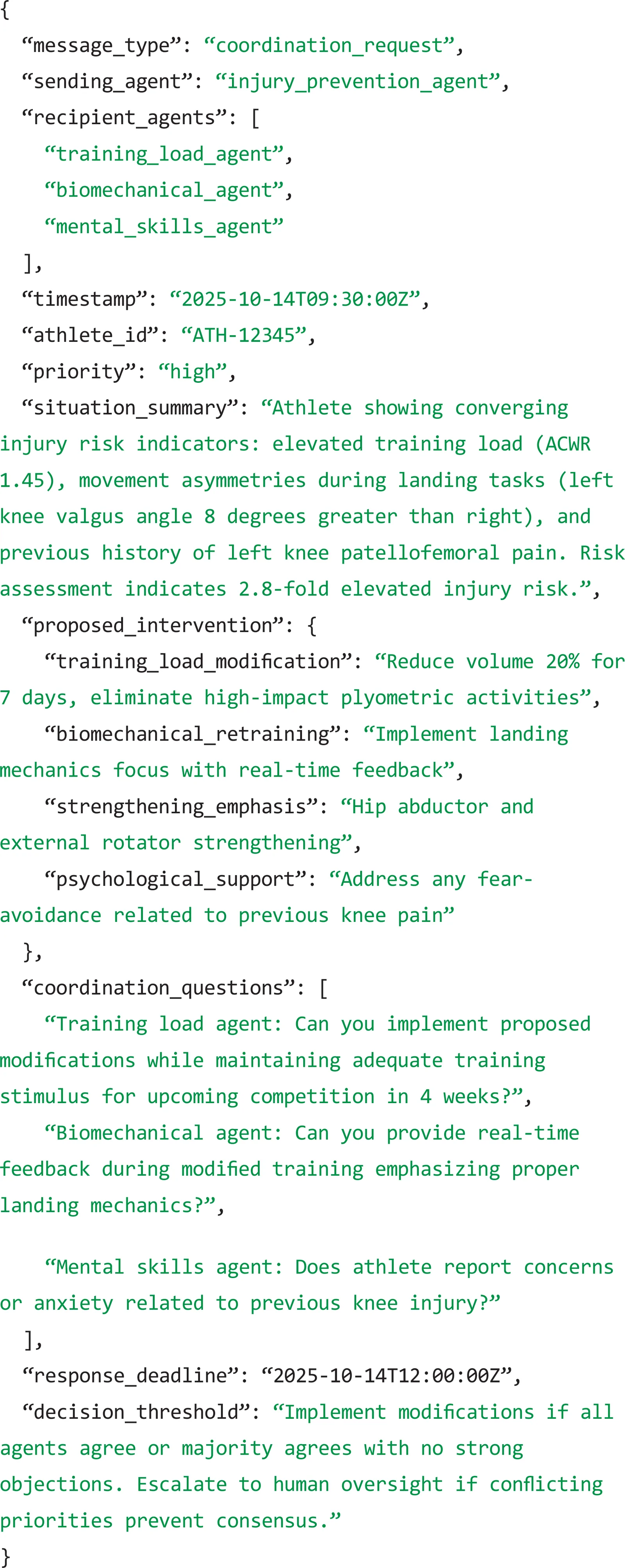


Each recipient agent analyzes the proposal and responds:

i) The TLMA responds as follows:

**Figure:**
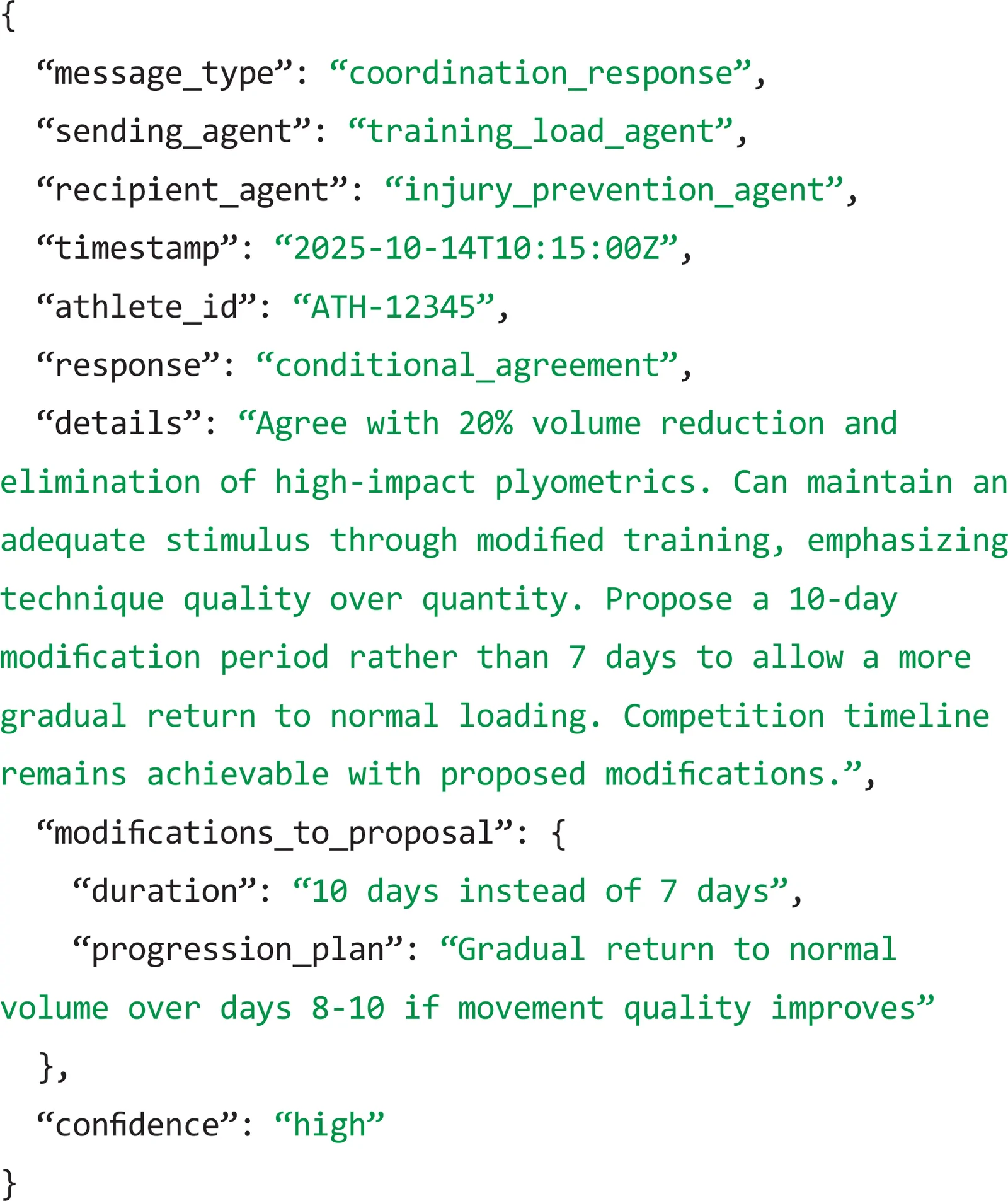


ii) The biomechanical agent responds as follows:

**Figure:**
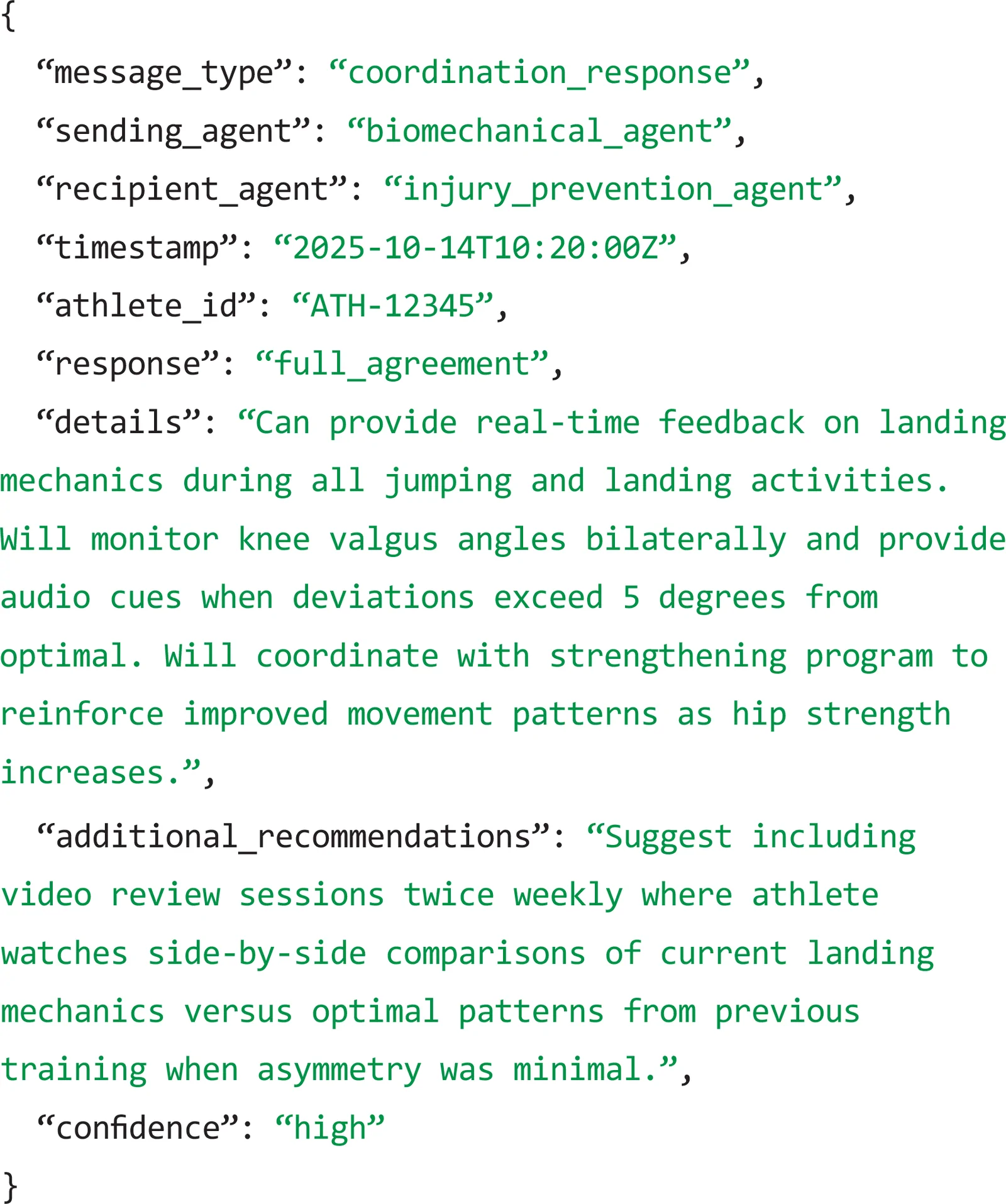


iii) The mental skills agent responds as follows:

**Figure:**
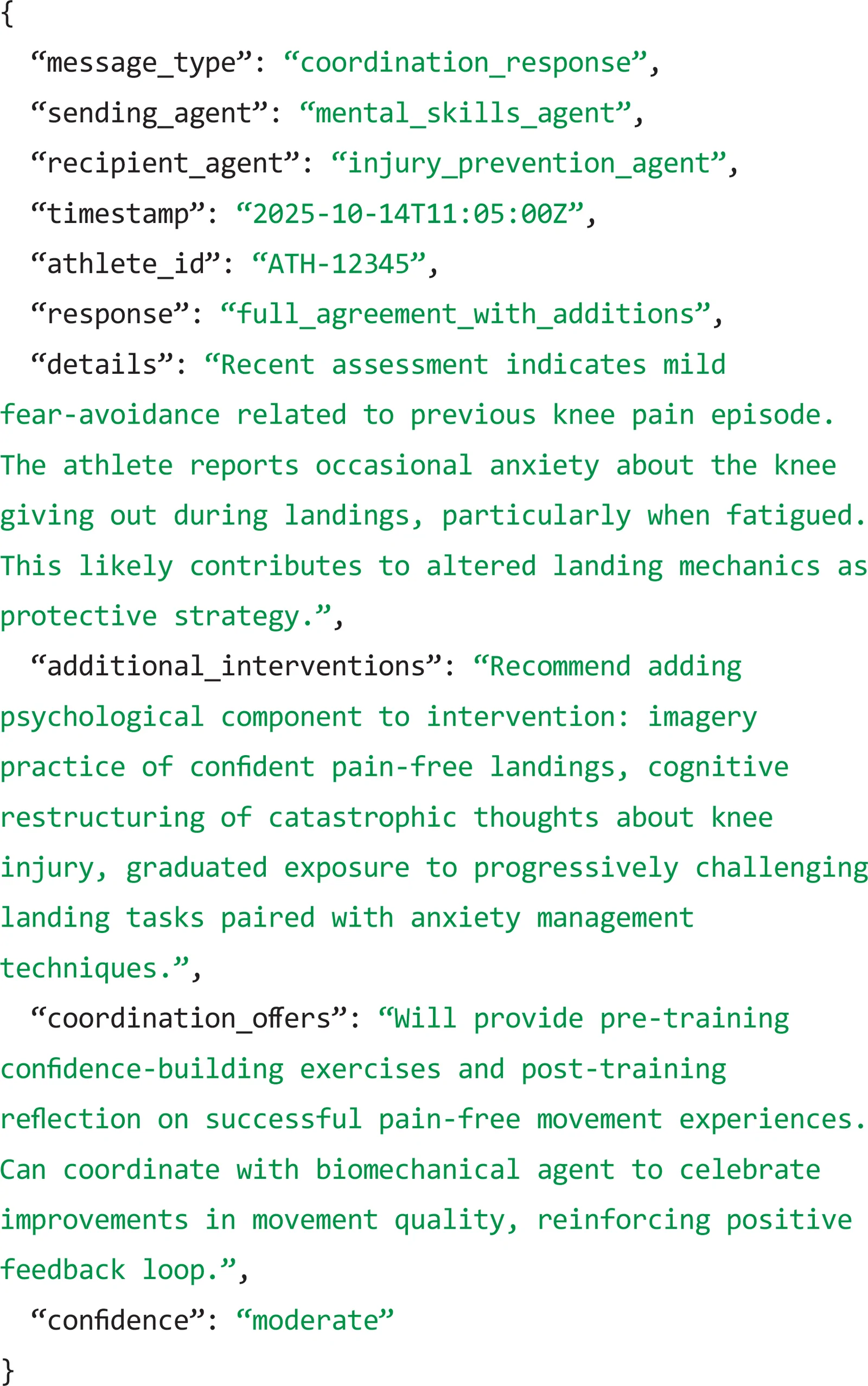


iv) The injury prevention agent compiles these responses, notes that all agents support the intervention with valuable additions, and synthesizes a comprehensive, coordinated plan. It sends the finalized plan to all agents and human supervisors.

**Figure:**
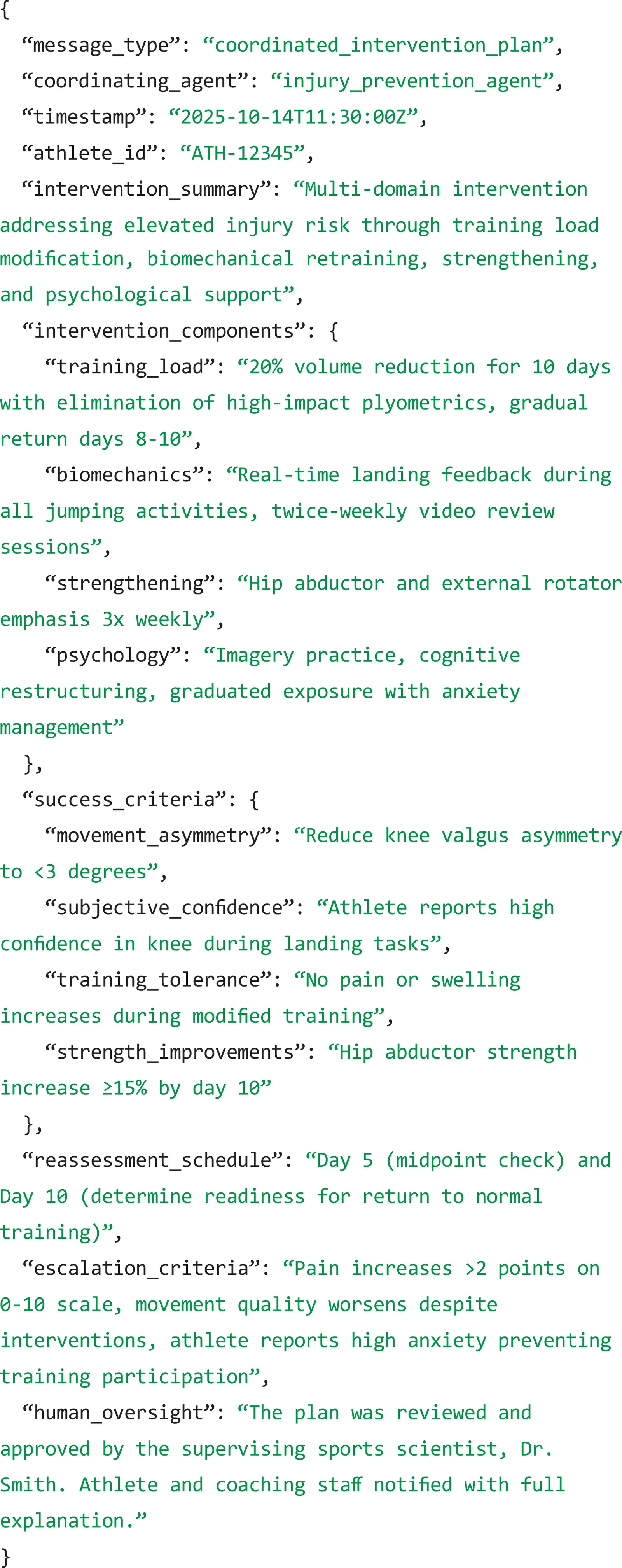


This coordination scenario demonstrates how multiple agents can collaborate to address complex situations requiring integrated solutions. No single agent possessed complete information regarding the situation. The injury prevention agent identified risk elevation but required workload context from the TLMA. The mental skills agent contributed to the psychological factors affecting movement patterns that the biomechanical agent had detected but not fully explained. Together, the agents developed a comprehensive intervention that no individual agent could have designed alone.

#### 3.14.3 Conflict Negotiation and Resolution Protocols

Not all coordination proceeds in a smooth manner. Agents sometimes recommend conflicting actions that reflect different domain priorities. The system requires robust conflict resolution protocols to determine how to proceed when agents disagree.

In a theoretical conflict scenario 12 weeks before a major competition, the TLMA would propose a 15% volume increase while the injury prevention agent would identify current hamstring asymmetry as a contraindication to increased sprint volume.

**Figure:**
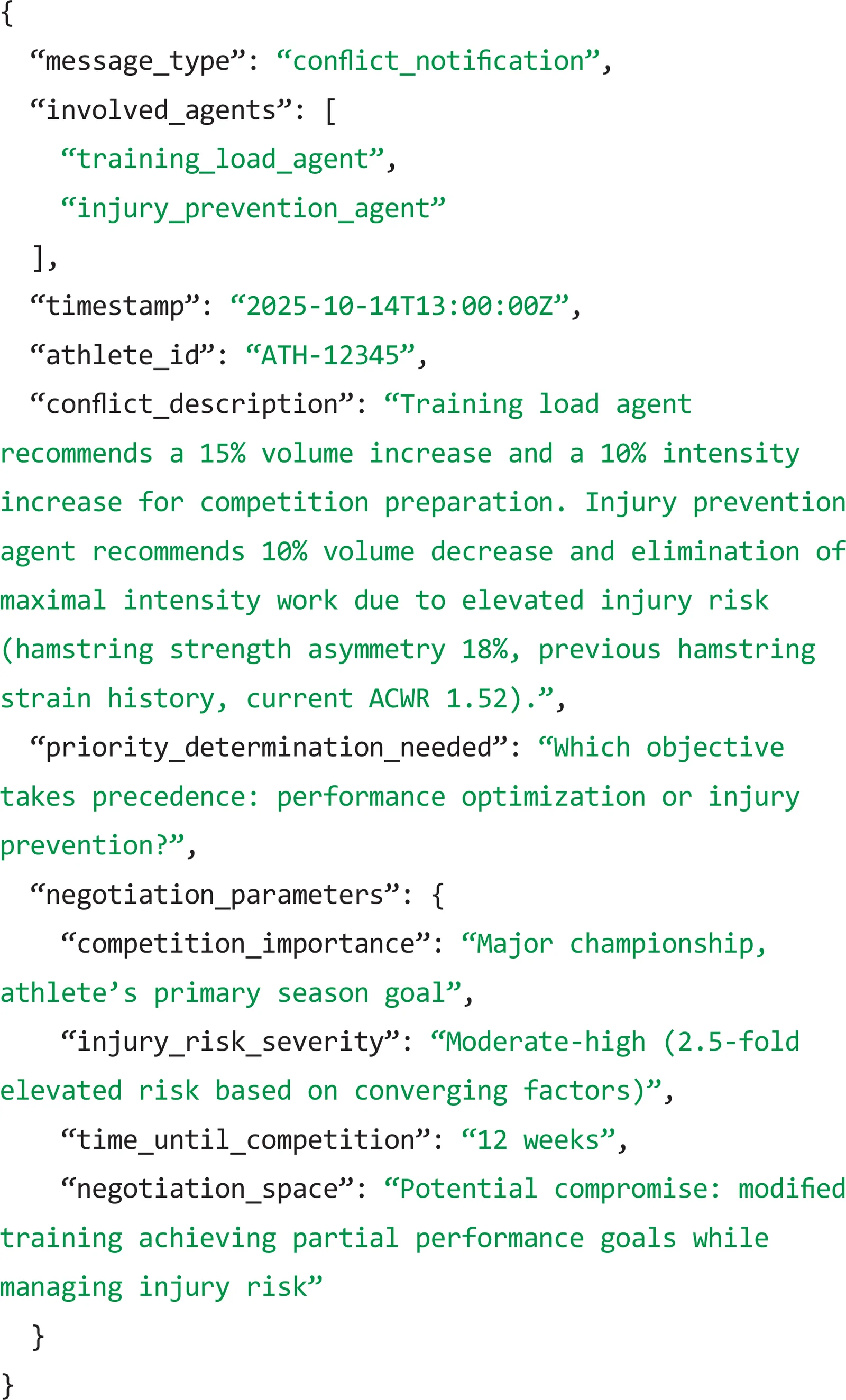


Autonomous negotiation would first be attempted: the TLMA would propose a compromise of 5% volume increase restricted to low-injury-risk activities (e.g., upper body, core, sport-specific skill work not involving maximal sprinting). The injury prevention agent would accept this conditionally, proposing an intensive hamstring strengthening protocol over three weeks, e.g., upper body, core, sport-specific skill work not involving maximal sprinting).

If asymmetry correction is achieved within that window, the TLMA would implement progressive volume and intensity increases over the remaining nine weeks.

If autonomous negotiation fails to reach agreement within preset parameters, the system would escalate to human oversight, transmitting a complete context package for practitioner adjudication.

**Figure:**
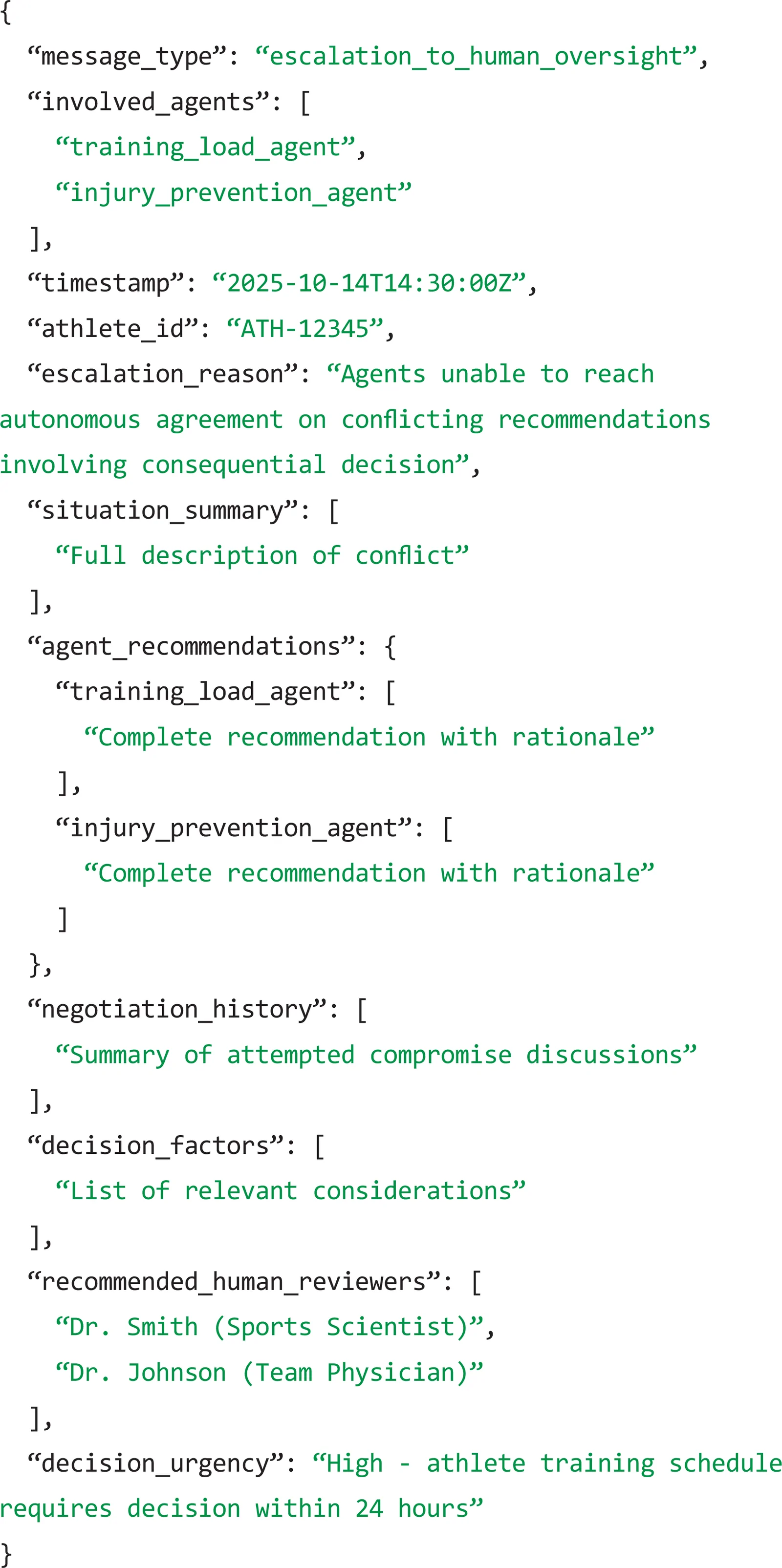


Human reviewers receive this comprehensive information package, discuss it with the athlete and coaching staff, and make final decisions considering factors beyond the agents’ data, including athlete preferences and values, organizational priorities, competitive context that may affect risk-benefit calculations, and medical considerations requiring clinical judgment.

#### 3.14.4 Priority Hierarchy for Safety-Critical Decisions

Certain decisions involve a clear prioritization hierarchy. We propose a safety-first hierarchy in which injury prevention and medical concerns override performance optimization goals. An injury prevention agent’s recommendation to reduce the training load takes priority over a TLMA’s recommendation to maintain intensity for adaptation stimulus. Medical contraindications identified by health-monitoring agents override all performance-focused recommendations.

This hierarchy reflects fundamental values: the health and safety of athletes in the long term take precedence over short-term performance gains. The system implements this hierarchy through hardcoded priority rules. When conflicts involve safety-critical decisions, the system automatically defers to the agent responsible for safety and health rather than attempting negotiation or escalating to human oversight. The decision is logged with an explanation, and human supervisors receive a notification; however, implementation proceeds immediately without awaiting human approval.

For example, if a HR monitoring agent detects potentially dangerous cardiac arrhythmias during training, it immediately terminates the training session, alerts medical personnel, and schedules urgent medical evaluation. The TLMA does not get input on this decision. Performance considerations become irrelevant when acute health concerns arise. This absolute priority ensures that safety never gets compromised through negotiation processes that might inappropriately balance safety against performance.

#### 3.14.5 Learning and Optimization through Outcome Observation

Multi-agent systems can improve coordination quality over time through outcome-based learning. The system tracks situations in which agents are coordinated, what decisions result from coordination, and what outcomes occur. ML algorithms analyze these logs identifying patterns.

For example, the system might observe that when the sleep agent reports poor sleep quality and the mental skills agent identifies elevated anxiety, subsequent training quality typically suffers regardless of planned training load. Learning this pattern, the system could proactively adjust expectations. When this combination of factors occurs, the TLMA automatically reduces intensity targets recognizing that physical capacity will be impaired by psychological and sleep factors.

However, this learning must occur within careful constraints. The system cannot modify fundamental safety parameters through autonomous learning. It can adjust how it weights various indicators within safe ranges and how it prioritizes different objectives in nonsafety-critical situations. All learning patterns undergo periodic human review to identify any concerning trends requiring correction.

#### 3.14.6 Proposed Validation Approach

Validating multi-agent coordination requires demonstrating that coordinated multi-agent systems produce better outcomes than independent single-agent systems or traditional human-managed approaches. Comparative effectiveness trials would contrast three conditions: athletes managed by coordinated multi-agent systems, athletes managed by independent single-domain agents without coordination, and athletes managed through traditional approaches. Primary outcomes would include comprehensive athlete health and performance measures encompassing injury rates across all types, training availability and consistency, performance improvements across multiple domains, and practitioner efficiency (e.g., time burden on sports science staff). Secondary outcomes would include coordination quality metrics assessing appropriateness of agent communication, effectiveness of conflict resolution, and efficiency of information sharing.

For example, a pragmatic trial might randomize collegiate athletic teams to three management approaches over an entire academic year. Outcomes would be assessed through comprehensive surveillance including injury tracking, performance testing, training session attendance and quality ratings, and practitioner time logs. The hypothesis predicts that coordinated multi-agent management will produce the lowest injury rates and highest performance improvements while reducing practitioner burden compared to other conditions. Qualitative research would explore stakeholder experiences with coordinated systems identifying which coordination features practitioners and athletes find most valuable, which aspects create confusion or concern, and how coordination quality could be improved. This information would guide iterative system refinements.

**FIG. 15:**
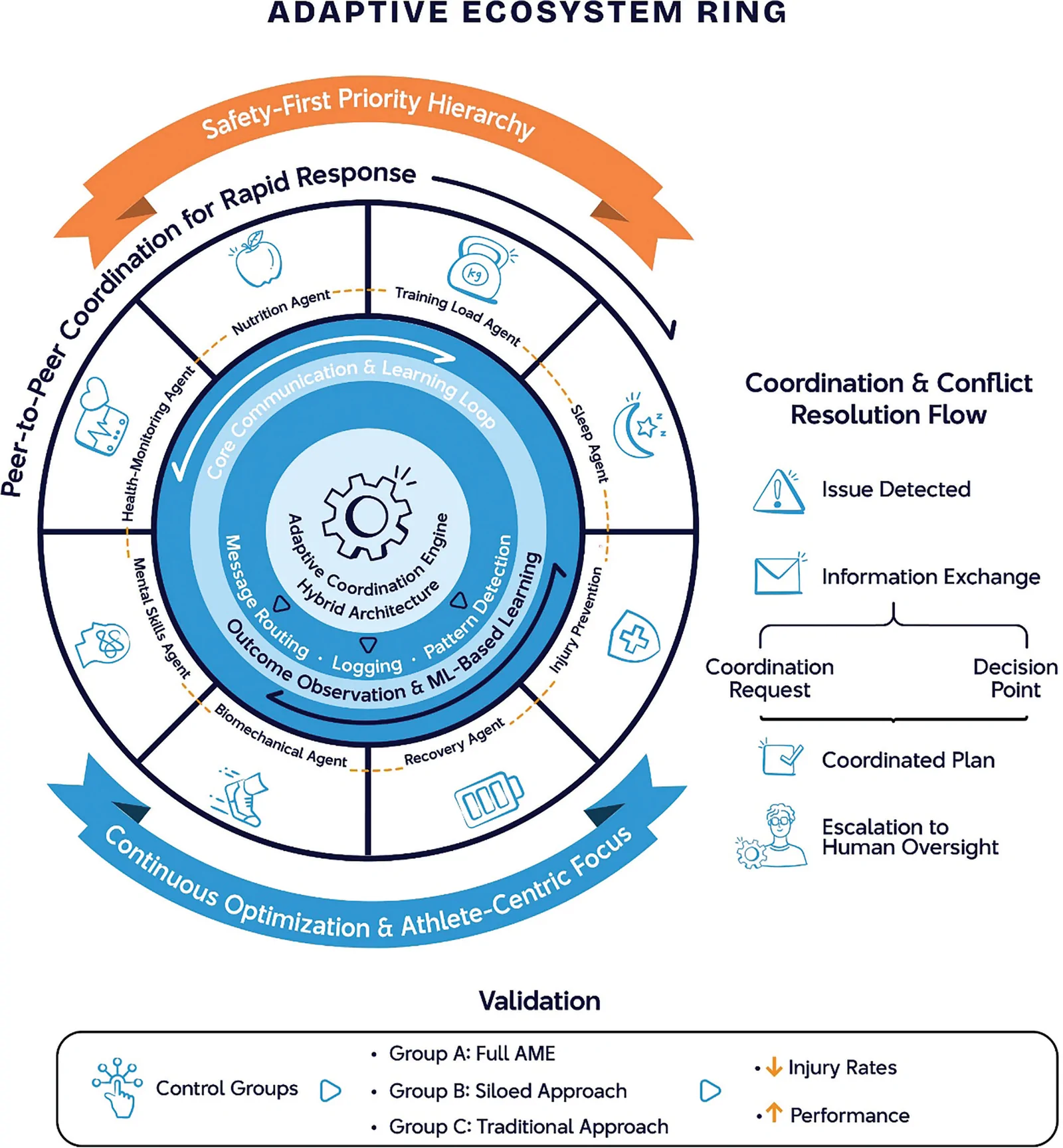
A Hybrid Multi-Agent System for Coordinated Athlete Management: The Adaptive Ecosystem Ring. AME: adaptive management entity. ML: machine learning.

Figure 15 represents a graphical abstract delineates the Adaptive Ecosystem Ring, a hybrid multi-agent system devised for the comprehensive management of athletes. Central to this system is the Adaptive Coordination Engine, which amalgamates centralized message routing with decentralized peer-to-peer coordination. This engine is encapsulated by a Core Communication & Learning Loop, symbolizing the perpetual flow of information, logging, pattern detection, and ML-based optimization that underpins the system’s intelligence. Encircling this core are Specialized Agent Modules, which are autonomous software agents tasked with specific domains of sports science, including training load, injury prevention, nutrition, sleep, recovery, biomechanics, mental skills, and health monitoring. These agents engage in Peer-to-Peer Coordination for Rapid Response through direct communication pathways. The entire system functions under a Safety-First Priority Hierarchy, ensuring that critical health and safety decisions are prioritized, and is guided by a principle of Continuous Optimization & Athlete-Centric Focus. A dedicated Coordination and Conflict Resolution Flow delineates the process from issue detection and information exchange to autonomous negotiation and, if necessary, escalation to human oversight. The Validation section proposes an experimental comparison across the full multi-agent ecosystem, individual unconnected agents, and traditional human coaching approaches, assessing impacts on injury rates and performance.

## 4 DISCUSSION

This narrative review constituted the first comprehensive examination of AI agent applications within sports science contexts, highlighting a notable scarcity of peer-reviewed academic research despite significant technological advancements and evident potential benefits. Our synthesis indicated that while conventional AI techniques have achieved substantial success in sports analytics, these implementations primarily serve as passive decision-support tools. It should be noted that these categories represent positions on a continuum; traditional AI systems vary considerably in their level of automation, and agent architectures range from simple reflex agents to sophisticated learning systems [15], as depicted in Figure 2, that depend on ongoing human intervention for execution. The evolution toward fully autonomous agent-based systems marks a transformative shift in the technological support available for optimizing athletic performance and managing athlete health.

### 4.1 The Critical Knowledge Gap and Its Implications

The lack of peer-reviewed research examining fully autonomous, continuously operating AI agent systems that integrate persistent monitoring, LLM-based reasoning, autonomous action execution, and coordinated multi-agent decision processes stands in marked contrast to rapid development in other sectors. As detailed in our *Introduction*, sectors such as healthcare [292, 293], manufacturing [294], and financial services [295] have successfully deployed agentic architectures for complex decision-making tasks. These implementations demonstrate that the technology exists and can be deployed effectively for continuous monitoring and autonomous intervention [19], including ML models achieving “area under the receiver operating characteristic curve” ^3^0.98 for early clinical deterioration detection [296], demonstrating that autonomous monitoring agents can operate with near-perfect precision in high-stakes clinical environments.

This knowledge gap creates both challenges and opportunities. The challenge involves lack of validated frameworks, implementation guidelines, and evidence regarding effectiveness. Practitioners interested in deploying AI agents find no established protocols, no comparative effectiveness data demonstrating superiority over current approaches, and no consensus regarding best practices for safe implementation. This absence increases implementation risk and may slow adoption among conservative practitioners, appropriately demanding empirical evidence before changing established workflows that, despite limitations, currently function adequately.

The opportunity involves positioning sports science researchers to define this emerging field from its inception. The first well-designed studies examining AI agent effectiveness will establish foundational knowledge shaping subsequent development trajectories for the coming years or decades. Researchers conducting these initial investigations will gain substantial influence over how the field evolves. Early adopters who document their implementation experiences will provide invaluable learning opportunities for others, potentially preventing costly mistakes and accelerating beneficial innovation diffusion. The absence of existing research means that careful, thoughtful work that will be conducted now, will have disproportionate impact on future directions compared to incremental contributions to mature research domains.

Several factors may explain why this knowledge gap exists despite clear technological potential and apparent applicability. First, sports science research traditionally focuses on physiological mechanisms, training methods, and injury prevention strategies rather than technology implementation and evaluation. The field’s cultural emphasis on biological and behavioral questions may have delayed attention to computational approaches even as these technologies matured. Academic incentive structures reward mechanistic understanding and theoretical contributions more than applied technology evaluation, potentially discouraging research in this domain. Second, AI agent technology itself remains relatively new. LLM-based agents capable of sophisticated reasoning, tool use, and autonomous decisionmaking only emerged prominently in 2023–2024 [297]. The lag between technological development and academic research publication typically spans 1–3 years through study design, data collection, analysis, manuscript preparation, peer review, and publication. Relevant studies may currently be in progress even if not yet published, and we may see rapid research expansion and output in the coming years. Third, industry may be developing AI agent applications without publishing findings due to competitive considerations. Professional sports organizations seeking technological advantages often guard their innovations closely to prevent competitors from accessing potentially valuable information. Commercial technology companies developing sports science applications may view their approaches as intellectual property requiring protection rather than open dissemination. This dynamic creates information asymmetry where substantial development occurs invisibly to the academic community. Fourth, regulatory uncertainty may inhibit both research and implementation. AI systems making health-related decisions may fall under medical device regulations in some jurisdictions; for example, the US Food and Drug Administration has developed a framework distinguishing clinical decision support software subject to regulatory oversight from exempt decision support tools [280], and analogous frameworks are under development in the European Union under the AI Act [298]. Liability concerns when autonomous systems make decisions affecting athlete health may discourage organizations from pioneering implementations until clearer legal frameworks exist.

Regardless of the reasons for the current gap, closing it rapidly would benefit the entire sports science community. We need empirical evidence regarding whether AI agents actually improve outcomes compared to traditional approaches rather than assuming benefits from technological sophistication. We need implementation science research identifying barriers and facilitators for successful adoption in real-world contexts with limited resources and competing priorities. We also need ethical analyses examining appropriate boundaries for autonomous decision-making in athlete management, where individual variability, unpredictable contexts, and value-laden tradeoffs characterize daily practice. Finally, we need cost-effectiveness studies determining whether benefits justify substantial development and implementation expenses that might alternatively fund additional human personnel or other resources.

### 4.2 Modular Development as Strategic Imperative

Our proposed three-phase implementation framework prioritizes modular development over comprehensive integration for several compelling reasons grounded in both technology adoption research and practical considerations specific to sports science contexts.

The modular approach reduces technical complexity to manageable levels at each phase. Developing a single specialized agent monitoring one single domain with well-defined inputs, decision rules, and outputs proves far simpler architecturally than building an integrated system managing multiple interacting domains simultaneously. This complexity reduction would accelerate development timelines by allowing focused effort on specific problems, reducing costs by requiring fewer developers with narrower expertise, decreasing probability of catastrophic failures during initial deployment by limiting system scope, and facilitating debugging when problems occur by providing clear boundaries for troubleshooting.

Early success with focused applications builds confidence and generates resources supporting more ambitious subsequent development. A TLMA that demonstrably reduces injury rates in its first validation study would create momentum for further investments. This would provide proof-of-concept evidence that autonomous agents can function safely and effectively in sports contexts. It would also generate publications establishing researchers’ expertise in this domain, producing revenues for commercial developers creating sustainability. The benefits of early modular success would create positive feedback loops accelerating subsequent development phases.

Modular implementation also facilitates efficient validation. Evaluating a TLMA’s effectiveness requires clear outcome measures such as injury rates, training availability, and performance markers. These metrics can be tracked straightforwardly in RCTs comparing agentmanaged versus traditionally-managed athletes. Evaluating a comprehensive integrated system’s performance proves far more challenging. How do we isolate which system components contribute to the observed outcomes? How do we determine whether observed benefits justify the entire system’s complexity and cost versus simpler alternatives? How do we identify which aspects function well versus poorly to guide refinements? These questions become tractable only after individual components have been validated independently, allowing subsequent integration studies to focus specifically on coordination benefits rather than confounding individual component effectiveness with coordination quality.

User acceptance increases when practitioners understand clearly, what each tool does and maintain control over complex decisions. A TLMA with well-defined authority parameters (e.g., can modify training volume up to 20% autonomously but must escalate larger modifications to human oversight as discussed earlier in our manuscript) and transparent decision-making processes (i.e., explaining why specific modifications occurred with reference to specific monitoring data) will likely encounter less resistance than a comprehensive system making opaque decisions across multiple domains through inscrutable algorithms. Practitioners can develop trust gradually, first accepting autonomous decisions for relatively simple situations before granting authority over increasingly complex scenarios requiring integration of multiple information sources.

The modular approach also accommodates heterogeneous implementation timelines across different sports science domains. Training load management benefits from mature data collection infrastructure through existing wearable devices deployed widely in professional and collegiate sports. Exercise prescription requires less sophisticated monitoring technology but depends on extensive exercise libraries with video demonstrations and progression algorithms. Nutrition optimization needs integration with food databases and meal planning systems. Mental skills training requires validated psychological assessment tools and intervention protocols. Each domain presents unique technical requirements and implementation challenges. Parallel development of specialized agents allows each domain to progress at its natural pace rather than forcing artificial synchronization that would delay some areas waiting for others to catch up or rushing some areas before adequate preparation.

However, the modular approach introduces its own challenges requiring attention and mitigation strategies. Practitioners may experience increased cognitive burden if multiple specialized agents operate independently without coordination, requiring manual information synthesis across platforms. Checking five separate agent dashboards and reconciling potentially conflicting recommendations could prove more burdensome than current fragmented systems, negating efficiency benefits the technology promises. This concern motivates *Phase 2* development of multi-agent coordination protocols. The coordination infrastructure should emerge naturally from *Phase 1* experiences identifying common coordination challenges and specific information exchange patterns that occur repeatedly.

Athletes may find interactions with multiple specialized agents confusing, preferring unified interfaces presenting integrated guidance. A collegiate athlete receiving separate notifications about training load modifications, nutrition adjustments, sleep recommendations, and mental skills practice assignments might feel overwhelmed by information volume even when each individual message provides value. User interface design must carefully consider information presentation and notification management to prevent end users from being overwhelmed.

Data redundancy can occur when multiple agents collect similar information through separate channels. The training load, nutrition, and sleep agents might prompt athletes to complete daily wellness questionnaires with overlapping questions. Consolidating data collection while ensuring that each agent receives the necessary information requires thoughtful database architecture and input coordination, even before formal multi-agent coordination protocols emerge.

Premature integration attempts risk-creating systems that are too complex to validate, debug, or maintain. Software engineering principles emphasize iterative development with frequent testing and user feedback [299]. Comprehensive integrated systems require everything to be right simultaneously, individual agent performance, inter-agent communication protocols, conflict resolution mechanisms, escalation pathways, user interfaces, and oversight systems must all function properly for the overall system to provide value. If any component fails, diagnosing the problem becomes challenging when all the components interact complexly. Modular systems allow the identification and correction of problems in isolated components before they propagate throughout the entire system, creating cascading failures that are difficult to troubleshoot.

### 4.3 Human Expertise Remains Indispensable

Throughout our review, we emphasized that AI agents must augment, rather than replace, human expertise. This principle stems from the fundamental limitations of AI that persist despite remarkable recent advances and the recognition that clinical judgment in sports science involves elements that resist computational formalization, including tacit knowledge [300] and context-sensitive ethical reasoning [31, 32].

AI systems excel in pattern recognition within their training distributions. Compared to human practitioners examining smaller personal experience samples, a TLMA trained on data from thousands of athletes may identify statistical patterns that predict injury risk more accurately. TLMAs maintain consistency across evaluations, avoiding the cognitive biases that affect human judgment. TLMAs continuously process information without fatigue or attention lapses. These capabilities represent genuine strengths that justify AI deployment.

Current sports science agent architectures lack established mechanisms for reliably quantifying their own uncertainty in novel situations, though uncertainty estimation methods such as Bayesian inference and conformal prediction are under active investigation in the broader AI literature [60]. It generates responses based on the patterns learned from its training data, even when these patterns provide poor guidance for novel situations.

Clinical judgment involves more than just pattern recognition. Expert practitioners integrate multiple information sources, including objective monitoring data, subjective athlete reports that may reveal concerns not captured by sensors, knowledge of individual history and context accumulated through extended relationships, understanding of current stressors (e.g., academic, occupational, relationship, financial) affecting capacity, and tacit knowledge gained through experience that practitioners struggle to articulate explicitly. They recognize when “something doesn’t seem right”; even when objective metrics fall within normal ranges, subtle changes in an athlete’s demeanor, voice tone, or movement quality that signal underlying issues before they manifest in measured data. AI agents are required to make decisions in situations involving competing values, yet lack the capacity for genuine ethical reasoning; such decisions must therefore involve human adjudication. Should an athlete with an elevated injury risk compete in an important competition, knowing that participation increases injury probability, but achieving performance goals matters deeply to the athlete/team? Should team interests regarding competition success take precedence over individual athlete interests regarding long-term health? These questions involve value judgments that AI systems cannot autonomously resolve. The «correct» answer depends on individual values, organizational philosophy, and contextual considerations that resist computational determination.

AI agents also adapt recommendations based on subtle communication cues, suggesting that an athlete may not adhere to the prescribed approaches. An athlete verbally agreeing to a training modification while displaying body language suggesting reluctance might require different communication strategies than an athlete enthusiastically embracing a change. Expert practitioners read these social cues and adjust their approach accordingly, thereby increasing adherence likelihood. AI systems currently lack embodied social cognition, which enables subtle interpersonal adaptation.

These capabilities emerge from (i) extensive deliberate practice, (ii) embodied experience working with athletes across diverse situations, (iii) supervised learning through mentorship, where novices observe experts’ reasoning processes, and (iv) reflective analysis of successes and failures, identifying principles guiding future decisions. This developmental process is not currently replicable by AI systems, irrespective of training data volume or computational scale [33, 34]. AI can access information and apply the learned patterns. It cannot replicate the judgment that characterizes human expertise in such matters.

Research documenting AI’s inability to replicate clinical reasoning is particularly relevant. Dergaa et al.’s [8] work examining ChatGPT’s exercise prescription capabilities revealed concerning deficiencies including inappropriate recommendations for individuals with medical contraindications, failure to recognize when referral to medical professionals was necessary, and provision of generic advice when individualized approaches accounting for complex factors were essential. While AI agents represent more sophisticated technology than conversational interfaces, such as ChatGPT, fundamental limitations regarding clinical judgment persist. Enhanced computational capability does not resolve the fundamental problem that machines lack genuine understanding and contextual reasoning. An agent recommending training load reduction would specify the ACWR value triggering the recommendation, the percentage decline in HRV from baseline, the athlete’s reported fatigue rating, and the basis for the chosen modification, enabling the practitioner to evaluate the reasoning critically.

Practitioners should maintain ultimate authority with easy-to-use override mechanisms that enable them to reject agent recommendations when their clinical judgment suggests superior alternatives. The agent should request reasoning when practitioners override suggestions, allowing the system to learn from disagreements and potentially revise its decision rules, but must not create barriers to prevent overrides. Practitioners might possess relevant contextual information that the agent cannot access (e.g., upcoming important competition, recent personal stressor affecting the athlete, injury history requiring conservative management), justifying different decisions than those recommended by the agent. The agent serves as a decision-support tool, not an autonomous authority. Human-in-the-loop interventions can create workflow friction within continuous autonomous systems. Agents should therefore operate on an Asynchronous Escalation Protocol. This protocol relies on algorithmic confidence thresholds. Agents execute routine, low-risk adjustments autonomously. They log these actions for later human review. Anomalous data or high-risk proposals trigger a Deferred Action State. The agent formulates a response but halts execution. It then sends a targeted summary to the practitioner’s dashboard. The practitioner must then authorize or modify the action. This workflow ensures clinical oversight. It also frees the practitioner from continuous screen monitoring.

Fourth, the agent must be configurable to accommodate different coaching philosophies and athlete population characteristics. Some coaches prefer conservative approaches prioritizing injury prevention even at modest performance cost. Others accept a higher injury risk to maximize performance gains, viewing injuries as inevitable costs of high-level sports rather than preventable outcomes. Neither philosophy is objectively “correct”; they reflect different value priorities. The agent should allow practitioners to adjust risk tolerance parameters within safe ranges, respecting philosophical diversity while preventing dangerous extremes. Youth athletes require different thresholds than elite professionals given developmental considerations. Agents must account for age, training maturity, and performance levels.

The two-tiered access model we propose reflects a pragmatic recognition that access to expert supervision varies dramatically across populations. Optimal implementation involves agents that support expert practitioners who retain decision-making authority. This supervised model maximizes the benefits while minimizing the risks through appropriate human oversight. Expert practitioners use agents to handle routine monitoring and documentation tasks, freeing up cognitive resources for complex cases that require nuanced judgment. They configure agent parameters based on individual athlete characteristics and coaching philosophy. They critically review agent recommendations, accepting appropriate suggestions and overriding inappropriate ones. They remain ultimately responsible for outcomes, with agents serving as sophisticated tools in their practice.

However, geographic isolation, financial constraints, and limited specialist availability create situations in which some individuals lack access to qualified supervision. Rural areas may not have certified strength and conditioning specialists within hundreds of miles. Developing nations may have very few sports scientists relative to their athletic populations. Recreational athletes typically cannot afford individualized guidance. For these underserved populations, despite inherent limitations, unsupervised agent access may provide better outcomes than the complete absence of structured guidance. The agent implements conservative approaches, prioritizes safety, provides extensive education about warning signs requiring medical attention, and encourages periodic professional evaluation when possible.

This pragmatic approach addresses health equity concerns while maintaining realistic expectations regarding the independent capabilities of AI. We should not pretend that unsupervised AI agent use equals human expert care. However, we should also recognize that perfect solutions are often unavailable, and imperfect options may still improve outcomes relative to current realities. An individual with T2DM in a rural area using an unsupervised exercise prescription agent may achieve better outcomes than receiving no structured exercise guidance at all, even though supervised care would prove superior if available.

### 4.4 Ethics and Risk Management

The deployment of autonomous AI agents in sports science contexts raises multiple ethical considerations that require explicit attention and proactive management strategies before widespread implementation.

#### 4.4.1 Autonomy and Informed Consent

When an AI agent makes decisions affecting an athlete’s training or health recommendations, the athlete deserves a clear understanding of what the system does, how it makes decisions, what authority it possesses, and what limitations it faces. Informed consent processes must explain these aspects in accessible language, avoiding technical jargon. Athletes should understand that agents make routine decisions autonomously within preset parameters but escalate complex situations for human oversight. They should know what data the system collects and how it uses this information. They should understand that agents have limitations and may make inappropriate recommendations in unusual situations.

Athletes should retain the right to decline agent-based management and receive traditional human-directed care. Organizations should not mandate agent use, recognizing that some individuals may prefer human-only guidance for personal, cultural, or philosophical reasons. Consent should be ongoing rather than one-time, with regular opportunities to reassess participation as athletes gain experience with how the system operates and its effects on their training and well-being.

#### 4.4.2 Data Privacy and Security

AI agents’ access and process sensitive personal information, which requires robust protection. Health data, performance metrics, injury history, and psychological assessments warrant confidentiality. The agent architecture must implement robust security measures. These include encrypted data transmission and storage, multi-factor authentication for system access, regular security audits to identify vulnerabilities, and predefined incident response protocols for potential breaches.

Elite athlete data is highly sensitive. Centralized cloud processing introduces severe privacy risks. Proprietary training strategies could easily leak through shared language models. The architecture must incorporate federated learning protocols. This approach trains the analytical model locally on the organization’s hardware. It only shares encrypted algorithmic updates with the central cloud system. The raw biometric data never leaves the team’s secure servers. This guarantees compliance with strict medical data regulations while allowing the global model to improve.

Athletes should understand what data the system collects, how it is used beyond immediate training decisions, who can access their data (e.g., coaching staff, medical personnel, administrators, and/or researchers), how long data will be retained, and procedures for data deletion when they discontinue agent use. They should have the right to access their own data, request corrections to inaccurate information, and control whether their data are used for research purposes or system improvement beyond individual care.

Particular attention should be paid to data sharing across organizational boundaries. When athletes transfer between teams, institutions, or countries, their data may need to be transferred with them. Clear policies should govern this process, ensuring that athletes control their information and that receiving organizations meet adequate security standards. When athletes retire or leave a sport entirely, their data should not remain accessible indefinitely without ongoing consent.

#### 4.4.3 Algorithmic Bias and Health Equity

Algorithmic bias represents a well-documented challenge in AI systems, with concerning implications for sports applications [301, 302]. If the training data over-represent certain demographic groups, the agent’s learned patterns may perform poorly for underrepresented populations. For example, an agent trained predominantly on male athlete data may generate suboptimal recommendations for female athletes. Key differences include menstrual cycle effects on performance and injury risk [303], greater relative incidence of ACL injuries [222], distinct optimal macronutrient distributions, and sexspecific psychological performance factors.

Similarly, agents trained on elite athlete populations may transfer poorly to recreational athletes or clinical populations with substantially different characteristics. Elite athletes tolerate training loads that can injure recreational athletes. They have access to resources (e.g., professional nutrition, physical therapy, recovery modalities) that algorithms might assume, but which typical users lack. Recommendations appropriate for elite contexts may prove inappropriate when these assumptions are not met.

Bias mitigation requires diverse training data to ensure that algorithms learn from representative samples across sex, race, ethnicity, age, ability levels, socioeconomic contexts, and geographic regions. Regular audits should assess performance across demographic subgroups to identify differential accuracy or effectiveness. When problems emerge, algorithms require adjustments through additional data collection targeting underrepresented groups, differential weighting of data from different sources, or explicit modeling of group differences when evidence supports this approach.

However, collecting demographic data to mitigate bias introduces privacy concerns. Athletes may question why systems collect information about race, ethnicity, or socioeconomic status. Transparent communication about bias mitigation helps address these concerns, explaining that diverse data improve algorithm performance for everyone rather than enabling discriminatory treatment.

#### 4.4.4 Accountability and Liability

When an AI agent makes a decision that leads to athlete injury, complex questions arise regarding responsibility. Who bears accountability? Which organization deployed the agent? Who are the developers who created it? Who are the practitioners who configured its parameters? The athlete who consented to its use. Clear accountability frameworks must be established before widespread adoption.

These frameworks should consider the agent’s level of autonomy, distinguishing between purely advisory systems that present recommendations that humans decide whether to implement and systems that execute decisions autonomously. They should account for the transparency of decision-making processes; opaque algorithms may warrant different liability distributions than explainable systems, where practitioners can evaluate reasoning. They should address the presence or absence of human oversight, supervised implementations where qualified personnel review decisions differ from unsupervised use. They should consider the nature of harm that occurred; minor inconveniences warrant different treatment than serious injuries with permanent consequences.

Legal scholars and policymakers should develop frameworks before controversies force reactive responses to them. Proactive attention allows for thoughtful consideration rather than rushed decisions following high-profile incidents. Sports organizations should consult legal experts before deploying agents to ensure appropriate liability insurance, contractual protections, and documentation practices.

#### 4.4.5 Over-Reliance and Deskilling Risks

The potential for over-reliance on AI agent recommendations poses risks, even in supervised implementation scenarios. Practitioners may defer to agent suggestions without engaging in independent critical analysis, consistent with automation complacency documented across complex human-machine systems [304]. This deference could lead to the gradual erosion of clinical judgment skills as practitioners lose opportunities for deliberate practice in developing their expertise. If practitioners routinely accept agent recommendations without question, they fail to engage in the reasoning processes essential for maintaining and developing their skills.

This concern is particularly salient for trainees. Student athletic trainers, novice strength and conditioning coaches, and early career sports scientists need extensive practice in making independent decisions with mentorship feedback to develop their expertise. If agents make most routine decisions, trainees may never develop the foundational skills. They might become dependent on technology, lacking the confidence and competence to practice effectively when technology fails or proves unavailable.

Recommended safeguards include mandatory practitioner review of randomly selected agent decisions, periodic audits comparing practitioner and agent recommendations to identify patterns of unquestioned acceptance, and training programs that develop independent decision-making competence before clinicians rely heavily on agent support.

### 4.5 Implementation Barriers and Success Factors

Understanding the factors that facilitate or impede AI agent adoption is essential for successful implementation. Technology adoption research and analogous implementations in healthcare provide insights into sports science contexts [305, 306].

#### 4.5.1 Technical Infrastructure Requirements

AI agents require integration with existing data collection systems, cloud-computing infrastructure for data processing and agent operation, secure data transmission and storage systems, and user interfaces that allow practitioners and athletes to interact with agents effectively. Organizations lacking a robust information technology (IT) infrastructure may struggle to implement agent systems, even if they perceive the value of the technology.

Professional sports organizations and well-resourced universities typically possess the necessary infrastructure or can develop it with reasonable investment. However, high school athletic programs, community sports clubs, and small colleges often lack the IT resources required for sophisticated technology implementation. Addressing this barrier may require the development of lower-cost implementation options that do not require substantial local infrastructure, cloudbased solutions where technology companies host systems reducing local infrastructure needs, partnerships where technology companies subsidize implementation costs in exchange for access to anonymized data for research purposes, or grants and philanthropic funding specifically supporting technology access for underserved organizations.

Phase 2 coordination requires seamless data exchange. However, the sports technology industry suffers from severe data silos. Manufacturers often restrict access to proprietary metrics. Autonomous agents cannot function without universal data integration. The field urgently needs an open-source data standard. Organizations must adopt frameworks similar to healthcare interoperability protocols. This requirement forces vendors to provide open Application Programming Interfaces. Without a standardized data architecture, crossdomain multi-agent negotiation remains impossible.

Continuous monitoring creates massive computational demands. Routing high-fidelity video or constant biometric data to cloud-based language models introduces latency. This delay makes real-time biomechanical feedback impossible. Frequent cloud queries also generate unsustainable financial costs for constant operation. Practical implementation requires edge computing. Systems must process routine data locally on the wearable device or local server. Developers should utilize Small Language Models for immediate, localized reasoning. The system only queries massive cloud models for complex, periodic schedule adjustments.

#### 4.5.2 Financial Considerations

Beyond initial implementation, recurring costs include software licensing or subscription fees, data storage and computing expenses that scale with user volume, technical maintenance, and periodic algorithm updates. Organizations should evaluate whether projected benefits justify these costs, given that cost-effectiveness data for sports science agent applications are not yet available. Organizations must evaluate whether the expected benefits justify these recurring costs.

Cost-effectiveness analyses comparing agent-based approaches to traditional alternatives can inform these decisions, although such analyses require effectiveness data that are not yet available. Preliminary analyses might use conservative benefit assumptions to determine the level of effectiveness that would justify the costs. For example, if an agent system costs $500 per athlete annually and traditional approaches cost $300 per athlete annually for similar monitoring through human personnel, the $200 difference is justified only if agents produce measurably better outcomes (e.g., reduced injury rates, improved performance) that are worth this investment.

For some applications, agents may reduce costs relative to traditional approaches. An exercise prescription agent providing individualized programming to clinical populations might cost less than an equivalent human personal trainer while maintaining effectiveness. If so, cost-effectiveness arguments support adoption, even in the absence of superiority, with effectiveness equivalence at a lower cost providing sufficient justification.

#### 4.5.3 Practitioner Acceptance

Practitioner acceptance likely varies based on several factors. Younger practitioners with greater comfort using technology may adopt agent systems more readily than practitioners trained when such tools were unavailable, who may view them with skepticism or feel threatened by automation. Practitioners with positive experiences using traditional AI analytics may view agents as natural extensions of existing tools. Conversely, those frustrated with previous technology implementations (e.g., clunky interfaces, unreliable outputs, increased rather than decreased workload) may resist new systems, regardless of their potential merits.

Organizational culture profoundly affects acceptance. Innovationoriented organizations that encourage experimentation and tolerate initial imperfections adopt novel approaches more readily. Risk-averse organizations that prioritize proven methods resist change until overwhelming evidence of superiority emerges. Top-down mandates to use agent systems without adequate practitioner input often encounter resistance, as practitioners feel that new tools are imposed upon them rather than selected collaboratively.

Involving practitioners in agent development and configuration increases acceptance. When practitioners help define agent authority parameters, contribute to decision rule development, provide feedback during pilot testing, and see their suggestions incorporated into system refinements, they develop ownership and understanding, improving adoption. In this regard, it has been shown that participatory design approaches, where end users actively shape technology development, produce superior outcomes compared to technology designed without user input [307].

#### 4.5.4 Athlete Acceptance

Athletes want evidence that agents improve outcomes, not just that they represent technological sophistication. Early implementations should clearly document and communicate their benefits. If, hypothetically, an agent-managed team experienced 40% fewer injuries than comparable teams using traditional approaches, such a result would constitute tangible evidence comprehensible to athletes; however, no empirical data currently support any specific effect size estimate. Performance improvements carry similar weight; athletes embrace tools that demonstrably help them achieve their goals.

Athletes also want assurance that agents respect their individual circumstances and preferences rather than applying rigid, standardized approaches. Agents must accommodate individual differences in training tolerance, recovery capacity, injury history, and personal preferences regarding training methods. Transparency helps athletes appreciate why recommendations occur with reference to their speci fic monitoring data rather than receiving unexplained directives from mysterious algorithms.

Finally, athletes need to be confident that their agents will operate safely and will not expose them to unnecessary risks. A transparent explanation of safety mechanisms builds this confidence. Explaining that agents automatically terminate sessions when dangerous patterns emerge, always escalate concerning medical symptoms to qualified clinicians, and maintain comprehensive logs allowing accountability and oversight helps athletes feel protected rather than vulnerable to technological failures.

#### 4.5.5 Regulatory and Professional Standards

Regulatory frameworks may facilitate or impede adoption, depending on their evolution. If professional organizations, such as the National Strength and Conditioning Association, National Athletic Trainers’ Association, or international sports medicine federations, establish standards endorsing agent use when properly validated and implemented, this legitimacy supports adoption. Position statements explaining appropriate agent applications, safety requirements, and practitioner oversight expectations provide guidance for encouraging responsible implementation.

If regulations create burdensome approval processes by treating agents as medical devices, even when used for general training optimization in healthy athletes, the implementation costs and timelines may increase substantially. Proactively engaging with regulatory bodies, helps ensure that frameworks appropriately balance innovation facilitation with safety protection. Regulations should prevent dangerous implementations without restricting beneficial applications.

#### 4.5.6 Operational Implementation Example: Agent-Based Conversational Workflow for Training Load and Recovery Synchronization

Addressing the implementation barriers outlined above requires concrete operational examples demonstrating how agent systems can be integrated into existing sports science practice.

**FIG. 16:**
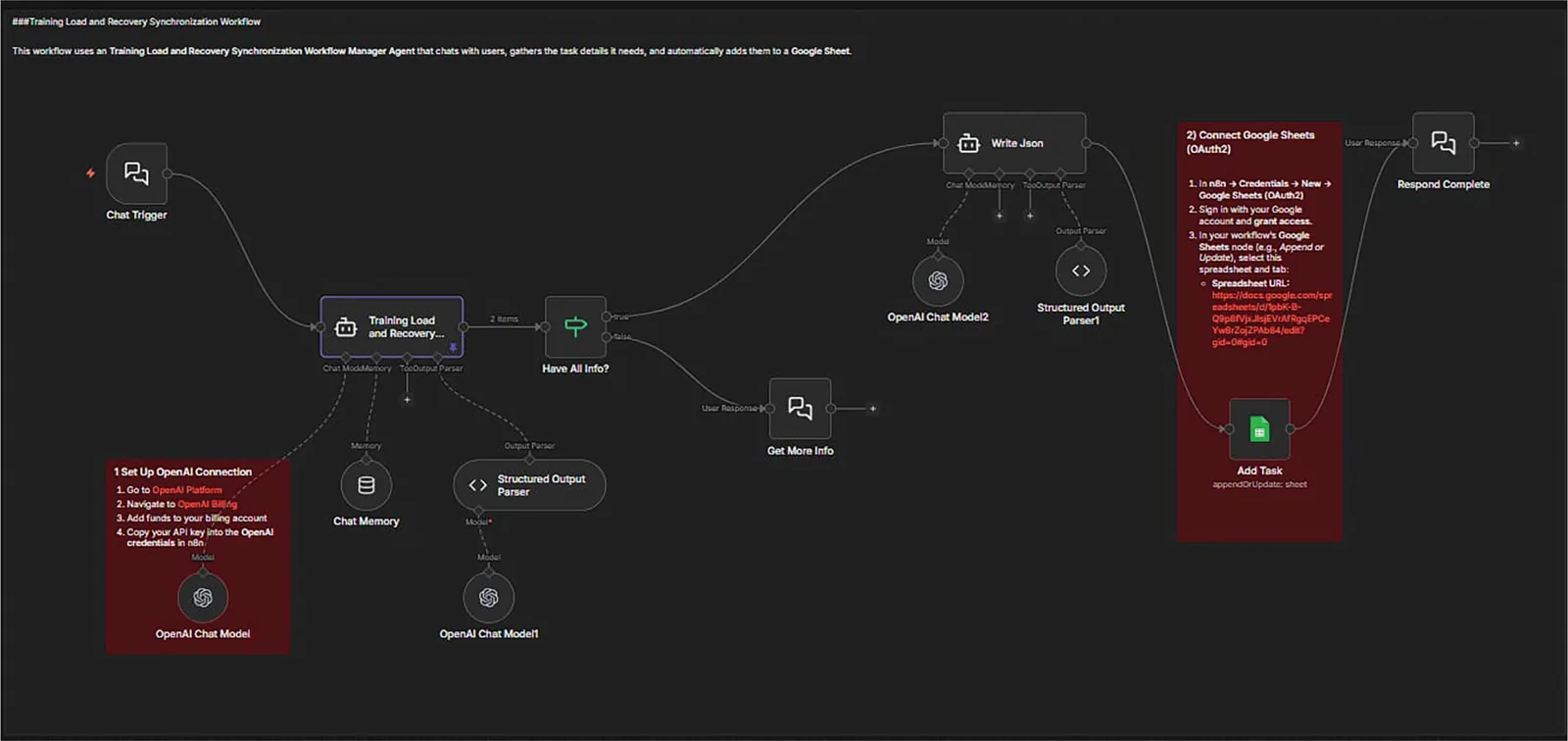
An n8n-Orchestrated Conversational Agent for training load and Recovery Synchronization.

Figure 16 represents practical illustration involves an agent-based conversational workflow designed to synchronize training load and recovery monitoring through structured human–AI interaction. This workflow operationalizes several success factors: it reduces technical infrastructure requirements by leveraging cloud-based solutions, demonstrates practitioner acceptance through participatory design principles (practitioners help configure decision thresholds and authority parameters), and builds athlete confidence through transparent data handling and explicit safety mechanisms.

The workflow is initiated through a chat-based interface enabling real-time conversational data collection. An OpenAI-powered conversational model collects training load indicators and recovery-related information. A structured output parser converts unstructured conversational data into a standardized JSON (for JavaScript Object Notation) schema, ensuring semantic consistency and interoperability across agent modules. A conditional validation node checks information completeness and re-engages the user through a targeted follow-up query where required. Once validated, a secondary model finalizes the structured data payload.

The validated dataset is then transmitted to a cloud-based persistence layer via OAuth2-authenticated integration with Google Sheets, where entries are appended or updated in a designated spreadsheet. This lightweight, auditable storage approach eliminates complex database infrastructure requirements while maintaining transparent logs that practitioners can audit, directly addressing safety-critical decision documentation and regulatory transparency expectations. The workflow concludes with a confirmation response to the user, signaling successful task completion. This architecture demonstrates how conversational AI, agent-based orchestration, conditional logic, and accessible cloud storage can operationalize athlete data capture while simultaneously addressing practitioner and athlete acceptance requirements, reducing financial and technical implementation barriers, and maintaining transparency necessary for responsible autonomous decision-making in sports science and digital health applications.

### 4.6 Research Priorities and Future Directions

The lack of sports science research concerning AI agents presents a broad research agenda encompassing various types of investigations. Prioritization of these areas should consider urgency and foundational significance, recognizing that limited research resources require strategic allocation. Key priorities include rigorous empirical validation of fully autonomous AI agent systems to establish their effectiveness and safety in real-world athlete management. Implementation science studies are essential to identify barriers and facilitators for adoption, integration, and sustained use within diverse sports settings. Ethical analyses must explore appropriate boundaries for autonomous decision-making, addressing concerns such as bias, accountability, and athlete autonomy. Additionally, cost-effectiveness evaluations are critical to determine the value proposition of deploying such systems compared to existing human-centered approaches. Addressing these priorities will guide the responsible development and deployment of AI agent architectures, ensuring they deliver meaningful benefits while mitigating risks.

#### 4.6.1 Immediate Priority: Validation Studies

Well-designed validation studies are urgently needed to examine whether specialized AI agents improve outcomes compared to traditional approaches. RCTs should compare TLMA with practitionermanaged training loads, agent-prescribed exercise programming with traditional prescription methods, and agent-supported injury prevention with conventional prevention approaches. These studies require adequate sample sizes for statistical power (power analyses based on expected effect sizes and variability), sufficient follow-up duration to detect meaningful outcome differences (likely requiring 3–12 months depending on outcomes), and comprehensive outcome assessment, including both benefits and potential harms.

Validation studies should be conducted on diverse populations. Most research will likely begin with elite athletes in well-resourced professional or collegiate settings, where infrastructure exists and research partnerships can be readily established. However, the findings may not be generalizable to recreational athletes with different motivations and resources, youth populations with developmental considerations requiring modified approaches, older adults with agerelated limitations, or clinical patients managing chronic diseases. Validation across multiple contexts ensures that claims about agent effectiveness rest are based on solid empirical foundations appropriate for the intended implementation settings.

Study designs should follow the CONSORT (for Consolidated Standards of Reporting Trials) guidelines for reporting randomized trials, ensuring methodological rigor and transparent reporting [308]. Primary outcomes should be registered prospectively to prevent selective reporting of favorable results. Intention-to-treat analyses should appropriately address missing data and non-adherence. Safety monitoring should identify potential harms early, with data safety monitoring boards for trials involving injuries or health outcomes.

#### 4.6.2 Implementation Science Research

Understanding how to implement AI agents successfully in real-world settings requires implementation science research examining barriers and facilitators to adoption, optimal training approaches for practitioners, effective change management strategies, and factors affecting their long-term sustainability. This research would employ qualitative methods: semi-structured interviews to explore stakeholder perspectives on acceptability and usability, focus groups to examine implementation challenges, ethnographic observation to document workflow integration, and organizational case studies of successful adoption.

Mixed-methods approaches that combine quantitative outcome data with qualitative process data explaining why implementations succeed or fail provide a comprehensive understanding. Frameworks such as RE-AIM (for Reach, Effectiveness, Adoption, Implementation, Maintenance) guide efficient evaluation across multiple implementation dimensions [309].

Implementation research should identify contextual factors affecting success, including organizational characteristics (e.g., size, resources, culture), practitioner characteristics (e.g., experience level, technology comfort, workload), and athlete characteristics (e.g., age, competitive level, motivation). This information allows tailoring implementation strategies to specific contexts rather than assuming a one-size-fits-all approach.

#### 4.6.3 Comparative Effectiveness Research

Multiple approaches to AI agent architecture exist, and empirical evidence should guide the selection. Do single-domain specialized agents produce better outcomes than general-purpose agents that attempt to manage multiple domains simultaneously? Does multiagent coordination improve outcomes compared to independent agents operating without communication, and at what point does the added system complexity outweigh the benefits? These questions require comparative effectiveness research that directly contrasts different architectural approaches.

For example, a trial might randomize athletic teams into three conditions: specialized single-domain agents without coordination, coordinated multi-agent systems, or general-purpose comprehensive agents managing all domains through unified reasoning. The primary outcomes included injury rates, performance improvements, practitioner efficiency, and system usability. Such research informs design decisions with empirical evidence rather than theoretical assumptions about what would work best.

Comparative effectiveness research should also examine different implementation models, including cloud-based versus locally hosted systems, subscription-based versus one-time purchase pricing models, and technology company-provided versus in-house developed solutions. Economic evaluations should accompany comparative effectiveness studies to ensure that decisions account for both clinical outcomes and financial sustainability.

#### 4.6.4 Ethical and Social Science Research

The ethical dimensions of autonomous decision-making in athlete management require scholarly attention beyond philosophical analyses. Empirical research should examine athlete and practitioner perspectives on appropriate boundaries for agent autonomy through surveys assessing comfort with different levels of autonomous decisionmaking, interviews exploring reasoning about when human oversight proves essential, and vignette studies presenting scenarios in which agents make autonomous decisions to assess ethical acceptability.

Future research should investigate the experiences of benefit and harm from agent use through longitudinal qualitative studies following athletes and practitioners using agents, critical incident analyses examining specific situations where agents helped or hindered, and comparative studies contrasting experiences across different populations and implementation contexts.

The factors affecting trust in AI agent recommendations warrant investigation. Future research should investigate the factors that promote or erode practitioner trust in agent recommendations, how trust develops or degrades over extended exposure, and the circumstances in which agents generate recommendations subsequently judged inappropriate, since these trust dynamics are essential for system design and implementation.

#### 4.6.5 Technical Computer Science Research

Computer science researchers should address several technical challenges specific to sports science applications. Developing explainable AI approaches that provide transparent reasoning is essential for appropriate human oversight. Current LLMs can generate post-hoc explanations, but these may not reflect actual decision processes. Developing methods in which reasoning processes remain interpretable while maintaining performance would substantially improve agent systems.

The development of robust methods for handling missing or unreliable data addresses practical implementation challenges. Wearable devices sometimes malfunction, or athletes forget to charge them. Athletes occasionally fail to complete subjective questionnaires or provide unreliable answers. Agents require methods for detecting data quality issues and making appropriate decisions despite incomplete information.

Designing agent architectures optimized for small data contexts is helpful, given that individual athlete datasets remain limited compared to large-scale medical databases. Transfer learning approaches, in which models trained on large general populations adapt to specific individuals with limited data, could improve personalization. Meta-learning methods where agents learn how to learn efficiently from small samples warrant exploration.

Advancing multi-agent coordination protocols that scale effectively as the number of specialized agents increases warrants attention. Current proposals involve relatively small numbers of agents (e.g., 5–10). Future systems may include dozens of specialized agents that address narrow domains. Coordination protocols must scale without communication overheads becoming unmanageable.

### 4.7 Limitations of this Review

Our narrative review has several limitations that require acknowledgment and consideration when interpreting our synthesis and proposals. Most fundamentally, our synthesis projects capabilities from other domains onto sports science applications without direct empirical validation in athletic context. We examined healthcare AI agent implementations and computer science demonstrations of agent capabilities and reasoned that similar approaches should prove effective for athlete management. This inferential approach introduces uncertainties. Sports performance involves unique characteristics that may present novel challenges for autonomous systems, including high individual variability in training responses that exceed medical populations, substantial psychological and motivational factors affecting outcomes that medical algorithms often ignore, and unpredictable competitive demands creating time pressures for recovery and performance that differ from patient care timelines. These characteristics may affect the agent’s effectiveness in ways that are not observable in healthcare implementations. The absence of sports science literature on AI agents meant that we could not compare multiple implementations, assess methodological quality across studies, synthesize quantitative outcomes through meta-analysis, or evaluate the consistency of findings across research groups. Our framework development relied on conceptual analysis and analogical reasoning, rather than empirical evidence. Specific claims about expected benefits (e.g., “agents will reduce practitioner cognitive burden by 30%”), optimal implementation approaches (e.g., “*Phase 1* should focus on these five specific domains”), and likely challenges (e.g., “practitioners will resist agents primarily due to autonomy concerns”) all require validation through deep research before they can be accepted with high confidence. Language restrictions to English publications potentially exclude relevant work from non-Englishspeaking research communities. Cultural differences may affect technology adoption preferences, human-AI interaction patterns, and ethical frameworks for autonomous systems. Coaching philosophies, athlete-coach relationships, and sports medicine practices vary across countries. Our synthesis primarily reflects perspectives from Englishspeaking countries (e.g., North America, United Kingdom, and Australia), which may not be generalizable to other cultural contexts. Rapid AI advancement means portions of this review may become outdated quickly. We conducted our analysis in October 2025, a period of extraordinarily fast development of foundation models and agent capabilities. Technologies emerging after our analysis period may expand or constrain the possibilities we envision. For example, if the capabilities of LLMs plateau rather than continuing to improve, some proposed agent applications requiring sophisticated reasoning may prove infeasible. Conversely, breakthrough advances in areas such as multimodal learning (e.g., integrating video, audio, and physiological signals) might enable applications that we did not anticipate. Future readers should consider this review as capturing the state of knowledge at a specific historical moment, rather than providing timeless insights. The authors’ perspectives inevitably influenced our analytical choices, despite efforts to achieve a balanced synthesis. Our backgrounds in sports science and exercise physiology shaped the agent applications we emphasized (e.g., training load, exercise prescription, biomechanics), how we framed implementation priorities (e.g., favoring applications we know well), and which concerns we highlighted regarding limitations and risks (e.g., reflecting our professional experience with athlete care). Computer scientists, physicians, and coaches may generate different frameworks emphasizing alternative considerations. Data scientists may emphasize the development of ML algorithms. Physicians may prioritize medical safety considerations. Athletes may focus more on autonomy and personal control. Acknowledging these perspectives will help readers critically evaluate our proposals.

Our proposed validation approaches remain theoretical and have not been field-tested. We suggest specific study designs, outcome measures, and methodological approaches that seem appropriate, but researchers conducting these studies may discover practical challenges that we did not anticipate. Recruitment may be more difficult than expected. The outcome measures may perform poorly. In such cases, statistical assumptions may fail. Experienced researchers may identify superior methodological approaches that we have overlooked. Our proposals should guide initial research planning but expect refinement through actual implementation.

Despite these limitations, we contend that this review provides valuable conceptual groundwork for an important emerging research domain. Our transparent documentation of the assumptions, limitations, and reasoning processes allows readers to critically evaluate the claims and identify specific aspects that require empirical validation. We clearly distinguish between established facts (e.g., traditional AI applications exist and function passively), evidence-based inferences (e.g., continuous monitoring enables faster interventions than periodic checking), and speculative proposals requiring validation (e.g., agent-managed training will significantly reduce injury rates). This transparency facilitates appropriate interpretation and constructive criticism, which will improve subsequent work.

## 5 CONCLUSIONS

Sports science currently relies on periodic observation and human decision-making when managing athletes. Our review explored how autonomous AI agents could enable continuous monitoring and proactive intervention in enhancing training outcomes and competition performance in elite sport. Over the past two decades (i.e., 2005–2025), advancements have primarily focused on data collection, visualization, and analysis. However, we are in a new era with emerging technologies profiling a future where real-time, contextaware decision-making supported by continuous monitoring will assume greater significance.

Many existing tools are predominantly passive, relying on dashboards that require practitioners to actively monitor and interpret data. This methodology can result in delays, particularly when significant physiological or behavioral changes occur in-between scheduled assessments. Theoretically, autonomous agent systems can mitigate this limitation by facilitating continuous monitoring, pattern recognition, and predefined responses. Such systems have the potential to decrease the practitioner’s workload while supporting more individualized interventions. However, these concepts remain largely theoretical rather than reflective of established practices.

Given the inherent complexity, variability, and ethical considerations associated with human performance and physiology, full automation is neither feasible nor desirable in this context. Consequently, a modular and incremental development strategy is deemed to be the most appropriate. Domains such as training load management and exercise prescription may serve as logical initial focal points, as they are relatively data-rich and involve more clearly defined decision boundaries than other domains. Demonstrating progress in these areas could facilitate trust-building, support validation efforts, and establish governance frameworks prior to broader implementation of AI in healthcare.

It is crucial to recognize that the advancement of automation should not be construed as a replacement for human practitioners. Rather, it signifies an evolution in professional roles. Sports scientists may increasingly assume the role of supervisors of intelligent systems, tasked with defining constraints, reviewing exceptions, and applying contextual judgment when automated logic is insufficient. Ethical reasoning, responsibility, and nuanced decision-making remain inherently human responsibilities, and the ultimate authority must always reside with qualified professionals who can be accountable while technology is not.

Beyond elite sports, these technologies may have significant implications for access and equity. Although expert oversight is typically available in high-performance environments, many recreational athletes and patients often lack structured guidance. Carefully designed non-clinical decision support systems, distinct from medical care, could provide a structured alternative in these contexts. However, this potential remains speculative and necessitates rigorous ethical and regulatory scrutiny.

However, substantial challenges remain. It is imperative to establish clear accountability frameworks, implement robust data protection protocols, devise strategies to mitigate bias, and institute safeguards against an excessive reliance on automated systems. Furthermore, there is a potential risk that overdependence on intelligent agents may impede skill development among early career practitioners, underscoring the necessity for educational models that emphasize critical thinking in conjunction with technological proficiency.

Our review highlighted a significant gap in the current body of evidence. Although conceptual promise is evident, it does not necessarily translate into real-world effectiveness. Future research should advance beyond mere predictions and rigorously assess outcomes through randomized pragmatic trials. This approach is essential to ascertain whether agent-supported interventions truly enhance health, performance, or injury prevention, compared to current methodologies.

In brief, the field of sports science has achieved significant advancements in observing and describing human performance. Agentbased systems present the potential for a transition toward a new era with more responsive and adaptive support mechanisms. The realization of meaningful benefits from this transition will be contingent on thoughtful design, rigorous empirical validation, and continuous human oversight.

## Data Availability

All data extracted and analyzed during this review are included in this article and its reference list. No additional unpublished material have been used.

## AI usage statement

In preparing this manuscript, the authors used ChatGPT-5.1 in October 2025 to assist with literature synthesis organization, manuscript structure development, and language refinement for academic style and clarity. Additionally, the accompanying video abstract was produced in April 2026. The script was authored by the researchers, while the visual content was generated using Google Gemini Veo and subsequently edited using CapCut.Following the use of these tools, the authors thoroughly reviewed, edited, and validated all generated text and visual content. The authors take full responsibility for the manuscript’s content, the accuracy of the video abstract, and all final conclusions [310].
